# Genetic diversity, population structure, and relationships in a collection of pepper (*Capsicum* spp.) landraces from the Spanish centre of diversity revealed by genotyping-by-sequencing (GBS)

**DOI:** 10.1038/s41438-019-0132-8

**Published:** 2019-05-01

**Authors:** Leandro Pereira-Dias, Santiago Vilanova, Ana Fita, Jaime Prohens, Adrián Rodríguez-Burruezo

**Affiliations:** Instituto de Conservación y Mejora de la Agrodiversidad Valenciana, Universitat Politècnica de València, 46022 Valencia, Spain

**Keywords:** Plant domestication, Plant breeding

## Abstract

Pepper (*Capsicum* spp.) is one of the most important vegetable crops; however, pepper genomic studies lag behind those of other important *Solanaceae*. Here we present the results of a high-throughput genotyping-by-sequencing (GBS) study of a collection of 190 *Capsicum* spp. accessions, including 183 of five cultivated species (*C. annuum*, *C. chinense*, *C. frutescens*, *C. baccatum*, and *C. pubescens*) and seven of the wild form *C. annuum* var. *glabriusculum*. Sequencing generated 6,766,231 high-quality read tags, of which 40.7% were successfully aligned to the reference genome. SNP calling yielded 4083 highly informative segregating SNPs. Genetic diversity and relationships of a subset of 148 accessions, of which a complete passport information was available, was studied using principal components analysis (PCA), discriminant analysis of principal components (DAPC), and phylogeny approaches. *C. annuum*, *C. baccatum*, and *C. chinense* were successfully separated by all methods. Our population was divided into seven clusters by DAPC, where *C. frutescens* accessions were clustered together with *C. chinense*. *C. annuum* var. *glabriusculum* accessions were spread into two distinct genetic pools, while European accessions were admixed and closely related. Separation of accessions was mainly associated to differences in fruit characteristics and origin. Phylogeny studies showed a close relation between Spanish and Mexican accessions, supporting the hypothesis that the first arose from a main genetic flow from the latter. Tajima’s D statistic values were consistent with positive selection in the *C. annuum* clusters, possibly related to domestication or selection towards traits of interest. This work provides comprehensive and relevant information on the origin and relationships of Spanish landraces and for future association mapping studies in pepper.

## Introduction

Peppers, chilies, and ajís, among other terms, refer to different forms of *Capsicum* spp., one of the most important cultivated vegetables in the world^1^. Thirty-one species are recognised in the genus, of which 26 are wild and five are cultivated^2^. The latter are: (i) *C. annuum* L., which is the most diverse, economically relevant, and studied species, and includes ‘bell’, ‘jalapenos’, ‘numex’, and ‘ancho’ types, among others; (ii) *C. chinense* Jacq., which includes very pungent peppers like the ‘habanero’ type; (iii) *C. frutescens* L. in which most known form is ‘tabasco’; (iv) *C. baccatum* L. or ‘ají’, which contains the ‘lemon drop’ and ‘ají escabeche’ as some of its most common forms; and finally; (v) *C. pubescens* Ruiz et Pav., which contains ‘rocoto’ and ‘manzano’ types^3–5^. At present, three complexes containing the cultivated peppers are distinguished based on the ability to cross-pollinate: (i) *C. annuum* complex, which comprises *C. annuum*, *C. chinense*, *C. frutescens*, their wild relatives, and *C. galapagoensis* Hunziker^6^, (ii) *C. baccatum* complex, which contains *C. baccatum, C. praetermissum* Heiser et Smith, and *C. tovarii* Eshbaugh, Smith et Nickrent^7,8^, and (iii) *C. pubescens* complex with *C. pubescens* and its wild relatives *C. cardenasii* Heiser et Smith and *C. eximium* Hunziker. Despite the fact that there are strong incompatibility barriers for hybridisation among these complexes, the development of viable hybrids, including hybrids between *C. annuum* and *C. baccatum*, has been reported in several works^9–11^.

The cultivated *Capsicum* species encompass a broad diversity as a result of evolution, domestication, and artificial and natural selection in agricultural environments in different primary and secondary centres of diversity^3,12^. In this regard, Spain is considered a secondary centre of diversity for peppers, especially for *C. annuum* which was brought mainly from Mexico just after the discovery of America^13^. Introduced as an alternative to Asian black pepper (*Piper nigrum* L.), capsicum peppers rapidly spread across Europe, Africa, and Asia^4^. In Spain, a process of more than 500 years of selection performed by generations of farmers created a plethora of ecotypes adapted to local conditions, many of which are still cultivated. This is more evident for the fleshy, big-fruited, bell-peppers called ‘Morrón’, named for their similarity to the nose of a sheep or a cow (i.e. *morro* in Spanish). Amazingly, *C. annuum* fruits and their derivatives have the largest number of EU Protected Designations of Origin (PDO) and Protected Geographical Indications (PGI) in Spain, such as ‘Arnoia’, ‘Pimiento Asado del Bierzo’, ‘Couto’, ‘Gernika’, ‘Morrón de Fresno’, ‘Riojano’ (PGI), and ‘Bola—Pimentón de Murcia’, ‘Padrón’, ‘Jaranda’, ‘Pimentón de la Vera’, or ‘Piquillo de Lodosa’ as registered PDO^4,14^. Despite that, most peppers production in Spain is based on F1 hybrids from ‘California Wonder’, ‘Lamuyo’, and ‘Dulce Italiano’ types, which have displaced the traditional and ancient materials mentioned earlier. However, the interest for the “taste of the past” by consumers and the challenge of adapting to climate change are contributing to the enhancement and reintroduction of landraces^14–16^. In addition, the studies on diversity of these materials are of importance in terms of: (i) genetic fingerprinting of varietal types, (ii) registration of materials, and farmers and communities rights preservation, (iii) genetic relationships in order to provide breeders with information about the available materials for breeding programmes, (iv) conservation of genetic resources, (v) development of non-redundant core collections, and (vi) revert the variability loss due to genetic erosion.

Despite its economical relevance, the development of *Capsicum* molecular tools lags behind other economically important *Solanaceae* crops, such as tomato or potato^17,18^. Its unusually large genome and repetitiveness may be the reasons for this delayed development^18–20^. In this respect, germplasm diversity analysis is one of the key elements for plant breeding and biodiversity conservation of *Capsicum* species^10,21^. However, it is highly dependent on the availability of informative genetic tools such as molecular markers^8,22^.

Throughout the last decade, high-throughput sequencing technologies development was stimulated by the need for low cost data and by the availability of faster analysis tools^23,24^. High-throughput genotyping-by-sequencing (GBS) has had an important impact on the scientific community due to its versatile application^23,25–27^. This approach can provide accurate results independently of the target species or population, and does not require having previous available genomic information^23,25,28^. GBS has been successfully used in pepper in recent years and an important amount of highly informative genome-wide SNPs were generated in each experiment. Germplasm diversity, population structure, and genomic selective sweeps analysis, as a result of domestication or local adaptation, are common to these works as it is an important first step for latter Genome-Wide Association Studies (GWAS)^29,30^. GBS-generated SNPs have been proven useful in the detection of trait-associated QTLs for both *C. annuum* and *C. baccatum* paving the way for further association studies and for a better understanding of pepper’s evolution^31,32^. To our knowledge, our work is the first to use GBS to analyse population structure and diversity and to assess selective genomic sweeps in a collection of Spanish landraces.

Herein we present a diversity study of a collection of *Capsicum* spp., encompassing four cultivated species and the *C. annuum* wild ancestor *C. annuum* var. *glabriusculum* using GBS. Our goals are genotyping a representative collection of Spanish heirlooms and ecotypes encompassing most varietal types of pepper, to shed light into the Spanish landraces phylogenetic relationships, among them and with other materials, to evaluate their molecular diversity and population structure.

## Material and methods

### Plant material

A diverse collection of 190 *Capsicum* spp. accessions encompassing five cultivated species, *Capsicum annuum* var. *annuum* (from now on *C. annuum*; 137 accessions), *C. chinense* (14 accessions), *C. frutescens* (2 accessions), *C. baccatum* (28 accessions), *C. pubescens* (2 accessions), and the wild form *C. annuum* var. *glabriusculum* (7 accessions), commonly known as ‘chiltepín’, was considered for GBS sequencing.

We report here the sequencing results for the collection mentioned above, although, since for 42 of the 190 accessions we did not possess a complete passport information those were excluded for downstream analysis. Hence, the 148 accessions subset was provided by the Universitat Politècnica de València Germplasm Bank, the COMAV Capsicum breeding group (112 accessions), several other research institutions (e.g. Institut National de la Recherche Agronomique (INRA-GEVES), Maritsa Vegetable Crops Research Institute (MVCRI), Mexico Chile breeding programme of Universidad Autónoma de Aguascalientes (UAA), Penn State University and United States Department of Agriculture (USDA); 23 accessions) and several seed companies (e.g. Batlle, Franchi Simenti, Intersemillas, Mascarell, Ramiro Arnedo, Reimer Seeds, and Zeraim Ibérica; 13 hybrids and heirloom lines) (Supplementary Data: Table 1).

The considered subset encompassed *C. annuum* (118) accessions, *C. annuum* var. *glabriusculum* (7), *C. chinense* (12), *C. frutescens* (2), and *C. baccatum* (9) (Supplementary Data: Table 1). Most of the considered accessions from *C. annuum* correspond to sweet, red, bell-shaped Spanish landraces, although a considerable amount of variability for pungency/sweetness, colour, fruit shape, resistances, origin, and varietal types was also included for a better evaluation of the diversity and phylogeny of the *Capsicum* genus (Supplementary Data: Table 1). Spanish *C. annuum* heirlooms and traditional materials have been prospected for more than 35 years in all regions of the country and, therefore, they can be considered highly representative of the variation of this centre of diversity in this species.

### DNA extraction, library preparation, and sequencing

DNA was extracted from young leaves using a modified CTAB protocol^33^. Raw and restriction enzyme *Hind*III (Thermo Fisher Scientific, Wilmington, NC, USA) digested DNA electrophoresis was run on 0.8% agarose gel to assure DNA integrity. Purity was assessed using Nanodrop® (ND-1000, Thermo Fisher Scientific, Wilmington, NC, USA) and quantity was assessed with Qubit™ (2.0 Fluorometer, Invitrogen, Carlsbad, CA, USA). High molecular weight DNA aliquots with 230/260 and 260/280 ratios ranging between 1.8–2.0 and 1.8–2.2, respectively, were then sent to Cornell University sequencing facilities (Ithaka, NY, USA). *Ape*KI methylation-sensitive restriction enzyme was used for library preparation as described by Elshire et al.^28^. Illumina sequencing adaptors and sample-specific barcodes were then ligated to the resulting fragments sticky ends and samples were pooled together for multiplexing. Polymerase chain reaction (PCR) was performed for library construction and Illumina HiSeq2500 (Illumina, Inc., San Diego, CA, USA) single-end technology was used for library sequencing. Generated good barcoded reads were captured, collapsed by similarity, and stored into a FASTQ file to generate unique tags. Only tags occurring at minimal count (≥3) were retained to generate a MasterTag file (FASTQ) as described by Elshire et al.^28^ and Glaubitz et al.^24^.

### Mapping and SNP calling

MasterTag file was aligned against the reference genome CM334 (Criollo de Morellos version 1.55)^20^ using Burrows–Wheeler Aligner (BWA version 0.7.8-r455)^34^ set to default settings. Aligned sequence tags were stored into TOPM (TagsOnPhysicallMap; SAM/BAM format^35^) file. SNP calling was then performed applying TASSEL-GBS Pipeline (version 3.0.173)^24,36^. Low-quality SNPs were filtered out by minimum minor allele frequency (mnMAF < 0.01) and missing data per site (MDpS > 10%), and finally converted into Variant Call Format file (VCF).

### Sequencing and SNP calling statistics

Sequencing, alignment, SNP calling, and population statistics were performed for a better understanding of results quality. SAMtools (version 1.8)^35^ was used to calculate the number of reads that passed the quality control and aligned successfully against reference. BEDtools (version 2.25.0)^37^ was used to assess the percentage of sequence tags that overlap with genic regions. Transitions/transversions ratio was obtained by BCFtools (version 1.8)^35^. And finally, heterozygosity was obtained by VCFtools (version 0.1.14)^38^.

### Population structure analysis

Evolutionary relationships and population structure were analysed implementing Rstudio (version 1.1.383)^39^. As a first step, VCF file resulting from SNP calling was subjected to another filtering process by SNPRelate package (version 1.12.2)^40^. Steps included: (1) removing multi-allelic, monomorphic, and low-quality positions; and (2) filter SNPs with a linkage disequilibrium (LD) threshold of 0.2. Initial analysis of population structure was carried out by principal components analysis (PCA) using the SNPRelate package^40^ and plotted using ggplot2 package (version 2.2.1)^41^.

To better understand and describe the genetic structure, discriminant analysis of principal components (DAPC) was applied using DAPC function from adegenet package (version 2.1.1)^42–44^. Most informative SNPs obtained by SNPRelate^40^ were used as input. DAPC workflow consisted of: (1) dataset transformation based on PCA, (2) determination of the optimal number of clusters by Bayesian information criterion (BIC) for *K* = 1–20 by *k*-means clustering with 100,000 iterations and 1000 randomly chosen starting centroids, (3) selection of *K* with lowest BIC as optimal number of clusters, selection of optimal number of PCs, and discriminant analysis (DA) functions to retain, and lastly (4) DAPC computation and plotting using ggplot2^41^.

For further elucidation of genetic distance between samples and clusters, a phylogenetic tree was constructed. For that, the aboot function from Poppr package (version 2.7.1)^45^ was run with the parameters bitewise.distance tree with neighbour-joining algorithm^46^ and 1000 bootstrap replicates. Plot.phylo function loaded from ape package (version 5.0)^47^ plotted the generated tree.

### Genetic diversity and selective sweeps

Population genetic diversity was calculated through Weir and Cockerham’s *F*_st_ index^48^ between all the clusters detected by DAPC. Tajima’s D statistic^49^ with a bin size of 500 kb was used to identify selective sweeps from our data for all the established clusters and plotted with ggplot2 package^41^. The mentioned statistics were obtained by VCFTools^38^.

## Results and discussion

### Sequencing and SNP calling

A collection of 190 *Capsicum* spp. accessions was successfully sequenced using the Illumina HiSeq2500 platform and yielding a total of 568,964,449 raw reads of which 524,450,716 (92.18%) represented good barcode reads. FASTQ file containing only collapsed and filtered reads was then aligned against the CM334 reference genome^20^. From the 6,766,231 unique read tags present in the file, 40.8% uniquely aligned, 7.4% multiply aligned, and 51.8% did not align successfully to the reference genome. Only 40.8% of sequences uniquely aligned to the reference genome, considerably fewer than the ones that do not align. These results could be due to (1) lack of reference for some positions in the reference genome, (2) the repetitiveness inherent to pepper genome contributing to two or more aligning sites for some sequences, (3) several accessions from species other than *C. annuum* probably could not align correctly, (4) some of the produced sequences could be mitochondrial or chloroplast DNA. The results are consistent with other recent works with this genus. Taranto et al.^29^ aligned uniquely 43.4% of tags and 9.8% aligned to multiple sites; and Ahn et al.^50^ obtained 45.5% of uniquely aligned sequence tags for a *C. baccatum* accession and 39.2% for a *C. annuum* accession.

**Fig. 1:**
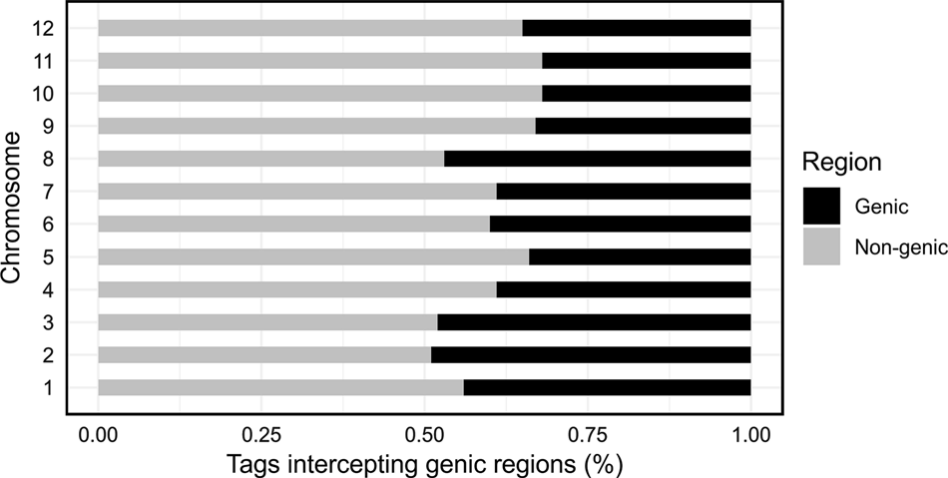
Distribution of filtered Illumina sequencing tags intercepting genic (black) and non-genic (grey) regions per chromosome

*Ape*KI was selected for genome complexity reduction due to its sensitivity to methylation and consequent frequent cut in gene-rich regions^28,51^. BAM file containing only mapped reads was compared against the previously available CM334 annotated genes gff3 (General Feature File) file in order to calculate the number of intersected genic regions. From a total of 3,260,848 tags, 39.3% overlapped genic regions. Our results indicate that chromosomes 2 (49%), 3 (48%), and 8 (47%) have the greater percentage of tags on genic regions (Fig. 1). Contrastingly, chromosomes 9 (33%), 10 (32%), and 11 (32%) presented the lowest values of tags overlapping genes, with only a third of total number of read tags being located inside these regions (Fig. 1). Taranto et al.^29^ using GBS technology and a widely diverse collection reported similar results. These results could be due to the GBS protocol, which targets preferably genic regions and has a lower genomic coverage especially for repetitive regions^51,52^.

Variant calling was performed by TASSEL-GBS Pipeline^24,36^ on the TOPM (BAM) file containing only uniquely aligned sequences producing 640,377 raw SNPs. Finally, after removing low-quality positions by MAF and MDpS, 531,680 SNPs, distributed along the entire genome, were selected (Supplementary Data: Fig. 1).

Transitions were found in greater proportion (59.7%). Transitions/transversions ratios are in agreement to other previous reports for pepper^29^. This phenomenon could be explained as an evolutionary advantage in case of mis-pairing because they are more likely to preserve protein structure than transvertions^53^.

The levels of observed heterozygosity for the 531,680 called SNPs ranged between 2.35% and 6.50% for these pepper accessions and averaged 3.16%. Regarding species, *C. annuum* var. *glabriusculum* (2.87%) displayed the lowest mean value, while *C. baccatum* (5.20%) the highest. And finally, experimental lines (3.10%) presented a lower mean value of observed heterozygote positions, while commercial hybrids (4.15%) presented the highest (Supplementary Data: Table 2). Values as low as the ones found here are not unusual for autogamous species such as pepper^54,55^. Lower mean values for observed heterozygosity were reported by Taranto et al.^29^ for a collection of 397 accessions from 8 different species (2.40%). However, Cheng et al.^56^ and Lee et al.^57^ reported higher mean values for bigger and more diverse populations than ours, 17.00% (ranging from 1.00% to 23.00%) and 15.00% (ranging from 9.00% to 21.00%), respectively. In another study, Nimmakayala et al.^32^ reported values in between the ones mentioned above (6.00%, ranging from 3.00% to 18.00%) for a diverse *C. annuum* population. The literature seems to support the perception that wild accessions have higher heterozygosity values than cultivated^57^. In this way, Ibiza et al.^22^ found a higher level of heterozygosity for *C. baccatum*, possibly indicating a higher level of allogamy than the others (Supplementary Data: Table 2).

### SNP filtering

A previous step to remove low quality and monomorphic positions by mnMAF, MDpS, and LD was performed. Original VCF with 531,680 positions was filtered by SNPRelate package^40^ resulting in a significant decrease to 4083 highly informative and well distributed across genome variants (Supplementary Data—Fig. 1; Supplementary Data—Table 3). Most pepper population diversity and structure analyses have relied on just a dozen to a few dozens of markers due to lack of data resolution of the peppers genome^12,57,58^. Fortunately, with the NGS technologies, thousands of markers are now easily available for researchers^28^. Thus, the most recent works used a similar number of markers to our study in order to assess genetic structure^30^ and for GWAS^32^.

### Population genetic relationships

**Fig. 2:**
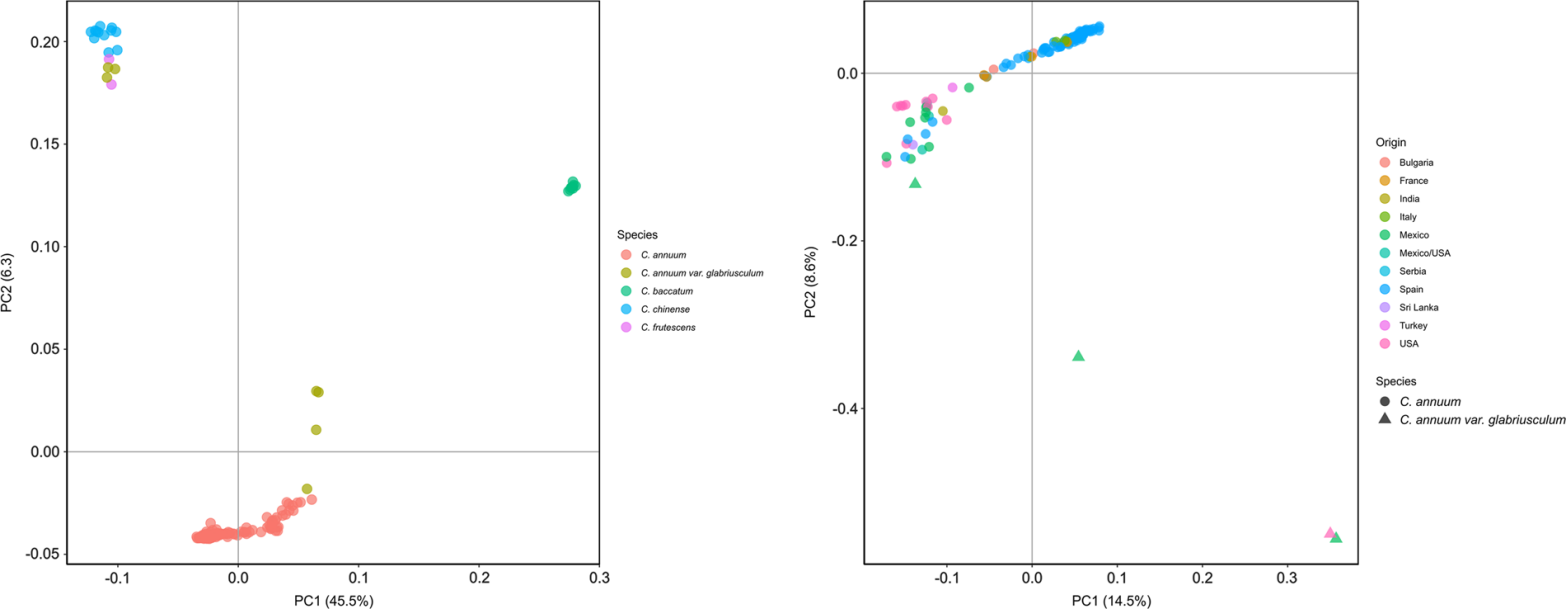
First and second principal components for both 148 accessions (left) and 118 *C. annuum* and closely related four *C. annuum* var. *glabriusculum* (right) based on 4083 filtered SNPs. Accession corresponding species (left) and origin (right) information is provided in a colour palette in each graph

The set of 4083 SNPs and SNPRelate package^47^ were used for PCA analysis. The first two principal components (PC) accounted for 45.5% and 6.3% of total variability, respectively (Fig. 2). The population can be divided into three different clusters: (i) one comprising all *C. annuum* plus four *C. annuum* var. *glabriusculum* accessions (mex_v1196, mex_q1078, mex_s1120, and usa_a1003), (ii) a second composed exclusively of *C. baccatum* accessions, and (iii) a plurispecific cluster formed by *C. chinense*, *C. frutescens*, and the remaining three *C. annuum* var. *glabriusculum* accessions. The first PC (PC1) separated species by complexes. In this way, the *C. annuum* complex (*C. annuum, C. chinense*, and *C. frutescens*) accessions displayed low and negative values for the PC1 whereas the *C. baccatum* complex accessions showed positive values. The second PC (PC2) differentiated *C. annuum* from the other species. Several studies using SSR^12,58^ and SNP markers^29,57^ described a similar separation of species. As in our case, Nicolai et al.^58^ and González-Pérez et al.^12^ reported a close relationship between *C. chinense* and *C. frutescens*. *C. annuum* var. *glabriusculum* peculiar distribution was also mentioned in Nicolai et al.^58^ and Taranto et al.^29^, where it was positioned near *C. annuum*, *C. chinense*, and *C. frutescens*.

In order to visualise in detail the relationships within the *C. annuum* cluster, a PCA was performed with only those accessions and the four closest *C. annuum* var. *glabriusculum* accessions, comprising 122 accessions. The PC1 (14.5%) and PC2 (8.6%) separated the accessions based on origin and fruit traits (Fig. 2). Sweet large-fruited Spanish and other European accessions (Bulgaria, France, Italy, and Serbia) clustered together, whereas pungent small-fruited North American (Mexico and USA) and Indian, Spanish, Turkish, and Sri Lankan accessions represented a much more diverse cluster of accessions. *C. annuum* var. *glabriusculum* mex_s1120 and usa_a1003 accessions clustered together and far away from the *C. annuum* group, mex_q1078 was at mid-distance from groups, and mex_v1196 was the closest to the cluster formed by the pungent *C. annuum* varieties (Fig. 2). Nicolai et al.^58^, Lee et al.^57^, and Taranto et al.^29^ reported a similar distribution based on fruit traits or geographic origin inside the *C. annuum* cluster.

### Population genetic structure

For further elucidation of the genetic structure DAPC was pursued. *K* values and lineal components to be retained were pre-determined using the find.clusters function. *K* = 7 was determined to be the most likely as indicated by BIC value (Supplementary Data—Table 4). The first 100 PCs and the first two DA functions were retained for the analysis, representing more than 90% of total variability. Notwithstanding, both *K* = 6 and *K* = 8 showed similar BIC values and cluster formation meaning that could also represent a good fit to our collection (Supplementary Data—Table 4).

**Fig. 3:**
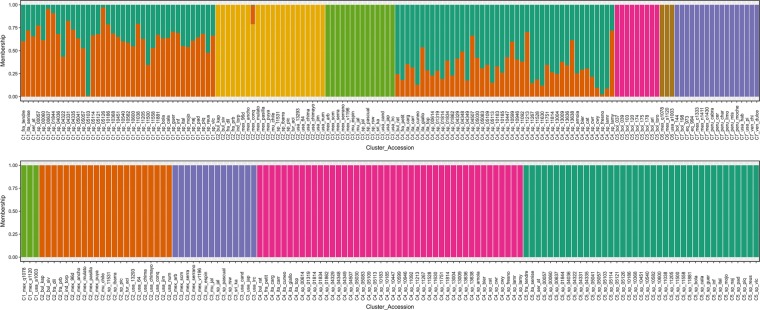
Population structure for both 148 considered accessions (top) and subset of 122 *C. annuum* accessions (bottom) given by DAPC with 4083 informative sites. Colours represent different assigned clusters. The *x*-axis provides accession names and respective assigned cluster whereas the *y*-axis provides the probability of each accession belonging to the assigned cluster

DAPC results were similar to those obtained by PCA, although with much more detail. Our population seems to be separated into seven clusters (Fig. 3). Cluster 1 comprised a set of 39 accessions, most of them Spanish landraces and three other European accessions (France, Italy, and Serbia) classified as *C. annuum* and sharing similar fruits traits: sweet, red, blocky peppers with variable fruit size and flesh thickness. Cluster 4 includes a diverse group of accessions composed of 44 European accessions, mostly *C. annuum* Spanish landraces and others with European origins (Bulgaria, France, and Italy) representing sweet, big sized, blocky peppers. Both Clusters 1 and 4 showed admixture and a narrow range of diversity between them. This was also observed by Nicolai et al.^58^, Lee et al.^57^, and Taranto et al.^29^ and the explanation probably resides on the fact that most Spanish varieties probably descend from a restricted number of individuals brought since Columbus journeys to the Americas and then spread across several countries which through selection and adaptation to local conditions originated a new range of forms^3,4,59^. Another reason why it is so difficult to differentiate European accessions may be the introduction of the same commercial lines in many different areas and this might be changing the genetic structure by cross pollinate with local varieties^22^. Cluster 2 was formed by 22 accessions, all of them *C. annuum* and encompassing a great diversity of places of origin. Most accessions in this cluster were collected in North American territories (Mexico and USA) with several fruit shapes. Europe was also represented with seven accessions from Bulgaria, France, and Spain, and finally one accession from India and another from Turkey. Bulgarian and French accessions were the only ones non-pungent; however, its fruit shape suggested that they could be improved lines developed from Mexican materials. Besides geographic origin, pungency could be a defining trait for population structure^12,29^. A group of 14 pungent accessions formed Cluster 3, including 13 *C. annuum* and one *C. annuum* var. *glabriusculum*. Most of them are Mexican varieties with cayenne and jalapeno shaped fruits and is completed by three Spanish accessions, one from Sri Lanka and one from USA, all pungent and with the same fruit shape. *C. annuum* var. *glabriusculum* is thought to be an ancestor to the Mexican *C. annuum*^2,60^ so its presence in this cluster is plausible and could be affected by the gene flow between domesticated forms and this botanical variety. Another hypothesis is that it could be a misclassification since *C. annuum* has an important range of phenotypes that could lead to an error during classification.

As in the PCA, all nine accessions identified as *C. baccatum* clustered together into a clearly differentiated group (Cluster 5; Fig. 3). This set of accessions seems to have a particular genetic print that makes them different from the rest of considered species. *C. baccatum* was domesticated independently from the other species in South America and it is still cultivated in isolated mountain areas that difficult the gene flow with other populations^22,61,62^. In addition, its crossability with species outside of its cytogenetic complex is difficult^63^. Ibiza et al. ^22^ reported a separation between Bolivian and Peru/Ecuador *C. baccatum* accessions corroborating that geographic isolation is an important factor for genetic structure.

Cluster 6 was composed of only three *C. annuum* var. *glabriusculum* accessions (mex_q1078, mex_s1120 and usa_a1003) that seemed to have a similar genetic print and were separated from the rest of accessions classified as the same species (Fig. 3). Nicolai et al.^58^ and Taranto et al.^29^ were not able to allocate this species into any group. The former reports three distinct genetic pools for this botanical group.

Finally, *C. chinense* and *C. frutescens*, as well as three *C. annuum* var. *glabriusculum* accessions (mex_c1333, mex_n1411, and mex_o1430), appeared to be indistinguishable accessions and were assigned to Cluster 7 as also seen in the PCA plot. The group includes 17 accessions from several regions of South and North America. Fruits are typically pungent, small sized, and with several fruit shapes. Many authors agree that *C. chinense* and *C. frutescens* should be considered as a single species^63,64^ and our results may support this hypothesis. However, *C. frutescens* was represented by only two accessions so solid conclusions could not be drawn. It is believed that a common ancestor through domestication in different locations gave place to *C. annuum*, *C. chinense*, and *C. frutescens* and the close relation seen in this work and others indicates that *C. annuum* var. *glabriusculum* could be that link^2,58^.

DAPC was also performed for *C. annuum* and the four *C. annuum* var. *glabriusculum* closest accessions. *K* = 5 was determined as the optimal number of clusters and the first 100 PCs and the first two DA functions were retained for the analysis (Fig. 3). Other *K*s presented a possible good fit as both *K* = 4 and *K* = 6 showed slightly higher BIC values (Supplementary Data—Table 4). Results seem to be in agreement with the ones observed in the DAPC with all the collection and the formed clusters were homologous between analyses (Fig. 3). DAPC clusters 1–5 for the 122 accessions corresponded to the DAPC clusters 6, 2, 3, 4, 1 for 148 accessions, respectively, the only difference being that admixture samples from the first plot are now two clearly distinct groups. The clusters 1–5 of *C. annuum* are now composed of 3, 22, 14, 44, and 39 accessions, respectively (Supplementary Data—Table 4).

### Population phylogenetic relationship

**Fig. 4:**
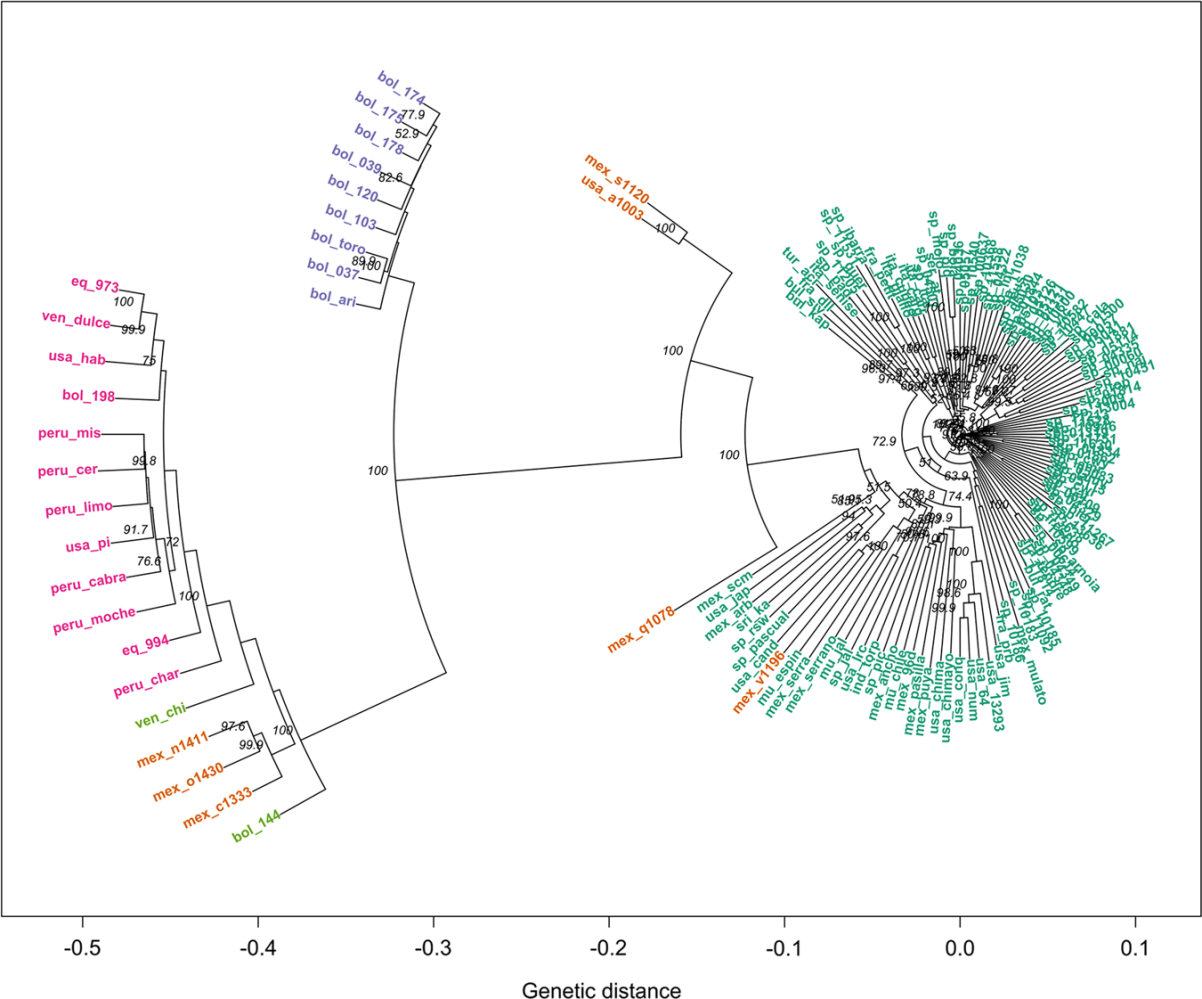
Clustering tree created from 1000 bootstrap replicates for the 148 accessions considering 4083 filtered SNPs. Dark green indicates *C. annuum* accessions, whereas *C. annuum* var. *glabriusculum*, *C. baccatum*, *C. chinense*, and *C. frutescens* accessions are represented in orange, purple, pink, and light green, respectively. Node values correspond to bootstrap values

**Fig. 5:**
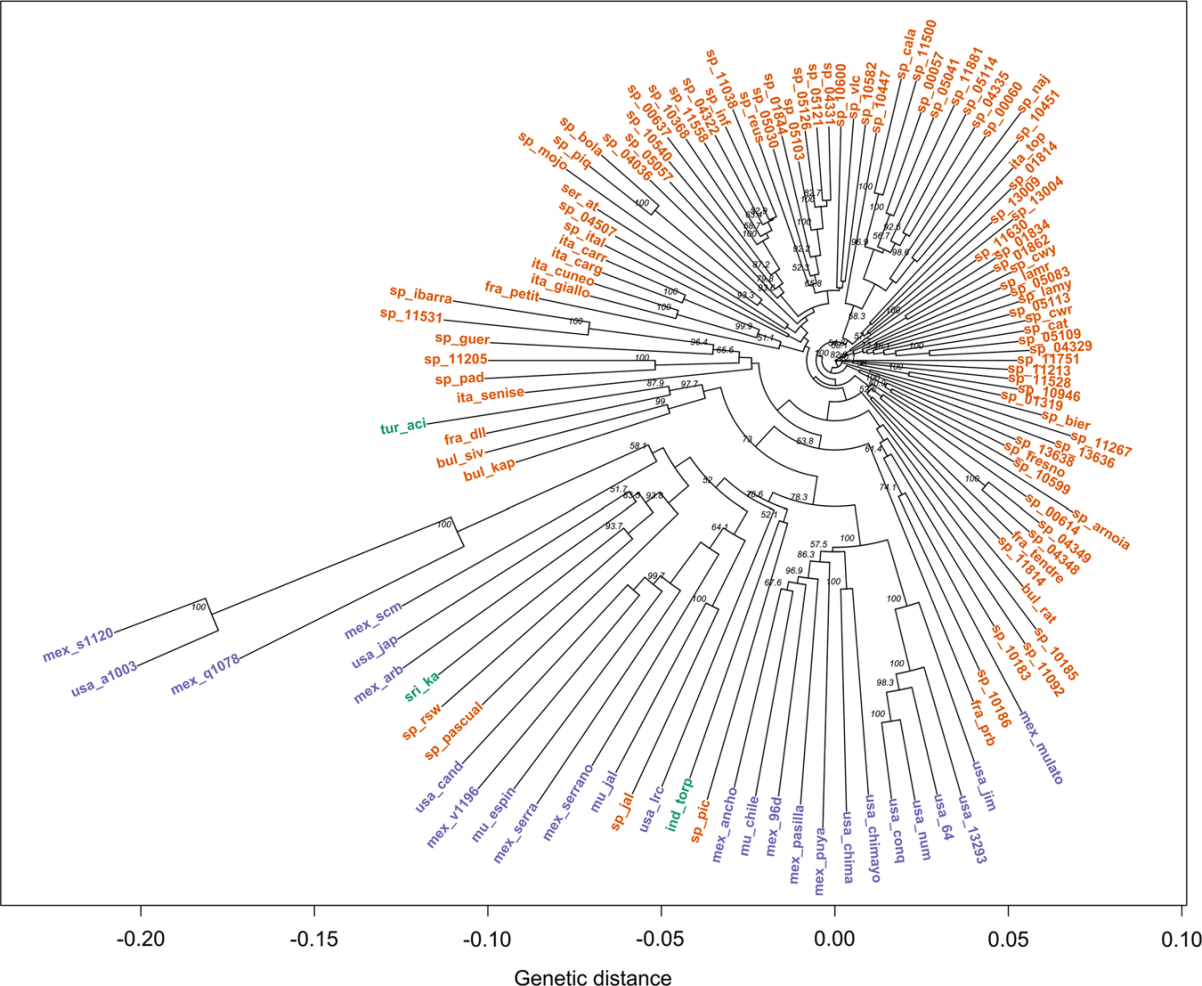
Clustering tree created from 1000 bootstrap replicates for the 118 *C. annuum* and closest four *C. annuum* var. *glabriusculum* accessions. Green indicates Asian accessions, orange the European, and purple the North American accessions. Node values correspond to bootstrap values

A phylogenetic tree for all the 148 accessions and another one only for the 122 *C. annuum* accessions were constructed for a better visualisation of sample distribution and relationships (Figs. 4 and 5).

*C. annuum* clustered separately from the rest of species, forming a large cluster. This is in agreement with previous results^22,57,58^. Genetic distance increased from European to North American accessions. As said before, Spanish varieties arose from the ones brought from Mexico since Columbus journeys and were bred into a range of different forms^3,4,59^. This explains the genetic closeness of many Spanish accessions to Mexican materials. From Spain it spread across Europe and therefore the difficulty to cut apart European lines^29,58^.

*C. annuum* var. *glabriusculum* accessions appeared scattered in the dendrogram. Four out of seven accessions appeared along the *annuum*–*chinense*–*frutescens* complex, of which mex_v1196 and mex_q1078 accessions were especially close, whereas mex_s1120 and usa_a1003 located further to the *C. annuum* clade. The remaining three accessions (mex_c1333, mex_n1411, and mex_o1430) located more closely to *C. chinense* and *C. frutescens* accessions (Fig. 4), hence indicating a possible link between those species and a possible common ancestry^2,58^.

*C. chinense* and *C. frutescens* grouped together into a clearly separated cluster (Fig. 4). In our case, the sample size was not large enough to make strong assumptions, given that *C. frutescens* was represented by only two accessions, but our findings seem to reinforce that *C. chinense* and *C. frutescens* should be considered the same species^63,64^.

Finally, the *C. baccatum* cluster located between the *C. annuum* main cluster and the *C. chinense–C. frutescens* group, although considerably closer to the second (Fig. 4). At first sight, these findings disagree with the works from González-Pérez et al.^12^ and Nicolai et al.^58^, who reported *C. frutescens* as the closest species of *C. annuum* followed by *C. chinense* and finally *C. baccatum*, or alternatively, Lee et al. (2016)^57^, who reported a slightly closer relation between *C. annuum* and *C. chinense*. Both groups of species, i.e. *C. baccatum* and *C. chinense–C. frutescens*, were domesticated independently but in relatively close regions, i.e. *C. baccatum* ancestors migrated to the south of Bolivia while the ancestral genetic flow of *C. chinense* and *C. frutescens* migrated towards the Amazonian basin where these species were domesticated^2,62^. By contrast, *C. annuum* arose farther away, in Mexico^65^. Thus, this geographical compartmentalisation might explain why our *C. chinense* and *C. frutescens* are closer to *C. baccatum* than *C. annuum*^2,62^.

Considering the tree containing only *C. annuum*, two groups can be identified: one mainly grouping Mexican and USA accessions, including the four chiltepíns (mex_s1120, usa_a1003, mex_q1078, and mex_v1196) and another including mostly *C. annuum* materials from Spain (Fig. 5).

In the first cluster, we found that ‘Chile Japones’ (usa_jap), several ‘serrano’ forms (mex_scm, mex_serra and mex_serrano), ‘jalapenos’ (usa_cand, mu_espin, mu_jal and sp_jal), and ‘Chile de Arbol’ (mex_arb) grouped the most closely to (wild) chiltepíns, as well as some pungent, thin-flesh cayennes like the Spanish ‘Guindilla Pascual’ (sp_pascual), ‘Picante Largo’ (sp_pic), the breeding line ‘RSW’, and the Indian ‘Torpedo of Bangalore’ (ind_torp) (Fig. 5). Our results seem to agree that ‘serrano’ peppers are close to the ancestral forms within the cultivated *C. annuum*. In fact they share some wild traits such as pubescence and soft flesh deciduous fruits^66^ and ‘Jalapeno’ peppers were mainly bred from serrano gene pools, thus the closeness.

In the second cluster, the closest materials to the North American accessions were the Turkish pungent ‘Aci Sivri’ (tur_aci) and sweet numex-like Bulgarian accessions (bul_siv and bul_kap), followed by another small cluster which grouped Basque ‘Guindillas’ (cayennes; sp_11531 and ‘sp_ibarra’), ‘Guernika’ (sp_guer), ‘Padron’ (sp_11205 and sp_pad), and ‘Peperone di Senise’ (ita_senise). These results show a close relationship among Turkish and Balkan materials, suggesting common genetic pool or historic exchange of materials between both countries. The phylogenetic proximity of some cayenne peppers like ‘Aci Sivri’ or ‘Guindilla de Ibarra’ to North American peppers like ‘Chile de Arbol’ was supported by their similarity in the pattern of volatiles^67^. In addition, these findings are in agreement with the history of Padron peppers, which were brought to Galicia (Spain) by the Franciscans from Mexico in the XVIIth century. This flow of materials from Mexico might have included also the ancestors of many ecotypes from other Northern Spanish regions like ‘Guernika’, with a similar fruit appearance to Padron peppers^14^.

Triangular-shaped Spanish materials from northern Spain (sp_10183, sp_10185, sp_10186, and sp_11092) and the Mexican ‘Mulato’ (mex_mulato) clustered closely to North American accessions, suggesting that Ancho/Poblano peppers from Mexico might be the ancestors of Piquillo peppers as suggested by Rodriguez-Burruezo et al.^67^ (Fig. 5).

The rest of *C. annuum* materials, mainly bell peppers, grouped in several subclusters. ‘Cuneo’ (ita_cuneo and ita_giallo) and ‘Carmagnola’ peppers (ita_carr and ita_carg), from the Italian Piedmont and characterised by large and slightly flattened ‘Morron’ (blocky) peppers, grouped with the French ‘Petit Marsellais’ (fra_petit) (Fig. 5). At first sight this is very surprising because ‘Petit Marsellais’ is a yellow-orange thin-fleshed small-fruited heirloom from the French Provence. However, its fruits look like small blocky peppers and, therefore, a few mutations relative to the fruit size and flesh thickness^68^ and/or the geographical proximity between the Provence and Piedmont might have enabled some genetic exchange.

Close to the subcluster of Italian-Piedmont peppers we also found other two groups: a small subcluster which includes ‘Pimiento de Mojo’ (sp_mojo), ‘Piquillo’ PGI (sp_piq), and ‘Bola’ PDO (sp_bola), and another larger group of Spanish ‘Morron’ peppers which encompasses materials from accession sp_05057 to accession sp_10582 (Fig. 5). Accessions in the first subcluster shared thin flesh and high dry matter content, useful for their culinary uses: the mojo picon (hot sauce) in Canary Islands, roasted and canned to be stuffed and ground to obtain pepper powder, respectively^4,14^. The second subcluster can be divided further into two groups of ‘Morron’ peppers. One group (from accessions sp_5057 to sp_11038) of large ‘Morron’ peppers (most fruits ≥150 g) from several Spanish regions; and a second group which comprises most ‘Valenciano’ accessions from Valencia and Murcia regions (sp_4331, sp_5030, sp_05103, sp_5121, sp_5126, sp_vlc, and sp_10582) as well as ‘Largo de Reus’ peppers from Catalonia (sp_reus, sp_01844, and sp_10600) (Fig. 5). Thus, despite a few accessions using these names can be found in other clusters (e.g. accessions sp_01862, sp_05113), these findings suggest a common genetic pool linked to such varieties along the Mediterranean coast of Spain (from Murcia to Catalonia), which can offer the opportunity of finding genetic fingerprints associated to the ‘Valenciano’ or ‘Largo de Reus’ denominations in the next future.

Finally, other four interesting subclusters are worth mentioning: (i) one ranging from accessions sp_cala to sp_10451, (ii) from accessions sp_11630 to sp_04329, (iii) from sp_11751 to sp_01319, (iv) from sp_bier (PGI Pimiento Asado del Bierzo) to sp_10599 (Fig. 5). The first encompasses most accessions from a particular type of ‘Morron’ peppers, commonly called ‘Morron de Conserva’ or ‘Morron de Bola’, with characteristic round/heart-shaped fruits (‘bola’ in Spanish) (Supplementary Data: Table 1). These varieties have been selected for being roasted and canned (‘conserva’ in Spanish) and, despite the lack of nose-shape of the true Morron peppers, they keep the term morron because of their thick flesh^14^. Calahorra peppers from La Rioja are considered the ancestors of this kind of Spanish peppers, which can be found throughout the country (Fig. 5). The second includes a miscellany of ‘Morron’ peppers from very different origins like breeding lines from seed companies and our research institute from Valencia, i.e. ‘California Wonder’ and ‘Lamuyo’ (modern ‘Morron’), and some ‘Valenciano’, ‘Largo de Reus’ and ‘Trompa de Vaca’ peppers (Fig. 5). With the only exception of sp_11603, all of them are from the Mediterranean coast of Spain, which suggests that at least two different lineages of ‘Morron’ peppers, the one with most ‘Valenciano’ and ‘Largo de Reus’ peppers and this one, are present in this region. The third and the fourth subclusters include ‘Morron’ peppers from the North of the country, but the former from Pyrenees to Cantabric Sea regions, while the latter from Leon and Zamora including two current PGI and ancient related ecotypes: PGI ‘Fresno de la Vega y Benavente’ (sp_fresno) and PGI ‘Pimiento Asado del Bierzo’ (sp_bier) (Supplementary Data: Table 1). Both PGI belong to different Pochard´s types, which suggests that genetic exchanges might have occurred among both varietal types in this region.

The three methods considered were able to detect, although with different levels of detail, the complex relations among the collection, and seemed to be coherent with each other. *C. annuum*, *C. baccatum*, and *C. chinense* separation was observed in all, as well as the incorporation of *C. frutescens* accessions into *C. chinense* cluster, and *C. annuum* var. *glabriusculum* distribution into two distinct genetic pools. Both PCA (Fig. 2) and DAPC (Fig. 3) offered an idea of the genetic structure behind the collection, however, phylogenetic tree (Figs. 4 and 5) gave a greater level of details on the relations among species and even among closely related accessions.

### Genetic diversity among clusters

The average weighed *F*_st_ value between the seven clusters was 0.486. The highest *F*_st_ value was observed between clusters 4 and 7 (0.739) and the lowest between 1 and 4 (0.069) (Table 1). Low values indicate a larger genetic difference among accessions intra-population than between populations, suggesting genetic flow between populations. High values suggest low genetic flow between populations and many genetic differences^48,69^.

**Table 1:** Weighed pairwise *F*_st_ values for the seven previously determined clusters. The increasing number of genetic differences between clusters are indicated with bold (low), bold italic (medium), and italic (high)

Spanish and other European accessions (Clusters 1 and 4) presented a lower level of genetic differentiation, suggesting a genetic flow between populations (Table 1). Similar results were reported by Nicolai et al.^58^, Lee et al. ^57^, and Taranto et al.^29^. This is probably due to the founder effect by a narrow diversity brought from the New World^3,4,59^.

Clusters 4 and 7 had the highest *F*_st_ value among all cluster combinations (Table 1). The first is mainly composed of European *C. annuum* accessions with low genetic variability whereas the second is an interspecific cluster composed of *C. chinense*, *C. frutescens* and *C. annuum* var. *glabriusculum*. Both clusters are geographically separated and the accessions have no genetic flow so the high *F*_st_ value is consistent with that scenario.

Cluster 5 also presented high *F*_st_ values regarding all combinations, which reveals that the population is isolated and has none or low genetic flow with other clusters, as expected for its geographic origin (Supplementary Data: Table 1). *C. baccatum* cultivation usually takes place in isolated areas that difficult crosspollinations. Furthermore, crosses outside its botanical complex are extremely difficult^22,63,64^.

Our data is consistent with previous published data. Taitano et al.^30^ reported a mean *F*_st_ of 0.821, ranging from 0.199 to 0.952, between 5 *C. annuum* landraces and *C. frutescens*. Nimmakayala et al.^31^ presented a fixation index of 0.780 between cultivated *C. annuum* and *C. baccatum* and 0.660 between wild accessions of those same species. Nimmakayala et al.^32^ reported for an only exclusively *C. annuum* with distinct levels of pungency and fruit weight an *F*_st_ between 0.020 and 0.150.

### Scans for selective sweeps

Tajima’s D statistic was used to assess possible genomic sweeps associated with selection for each cluster formed. Genomic regions with low or negative Tajima’s D values indicate an unusually high number of high-frequency variants due to a balanced selection. On the other hand, high positive values are due to an excess of rare variants which can be result of a positive selection^49^. Cluster 1 displayed the highest weighed mean Tajima’s D value (0.854), while cluster 5 presented the lowest value (0.356) (Supplementary Data: Fig. 2).

Clusters 1–4, composed mostly by cultivated *C. annuum* accessions presented several regions with positive Tajima’s D values indicating a positive selection, possibly related to domestication and/or the pressure to achieve a specific phenotype resulting in an accumulation of trait related mutations^31,32,58^. High Tajima’s D values for a region spanning 7.5 Mb in the final part of the chromosome 1 for clusters 1 to 4 was found. QTLs implied in fruit weight, length and diameter, and pedicel length were described for this region so this could be an indication of selection for such traits^70–72^. Chromosome 5 showed a possibly purified region in the last positions for clusters 1 and 4, spanning 6 Mb and possibly linked to fruit diameter^72,73^. Finally, Clusters 2–4 showed high Tajima’s D values for a 1 Mb region at the end of chromosome 6. This region is linked to the control of several fruit traits such as weight, diameter, pericarp thickness, length, and shape^72–75^. Bear in mind that the resolution is insufficient to make strong assumptions however it sheds light into future association mapping studies.

Contrarily, clusters comprising only *C. annuum* wild ancestor *C. annuum* var. *glabriusculum* and closely related *C. chinense* and *C. frutescens* species presented a Tajima’s D distribution closer to the neutral or balanced selection (Supplementary Data: Fig. 2). This was expected due to the biological status of these accessions (Supplementary Data: Table 1). They are not as exploited as the first four clusters and are often cultivated in open-pollination conditions^60,76^. Therefore rare alleles are maintained at low frequencies.

*C. baccatum* cluster (Cluster 5) presented the lowest values, indicating a patron of none or little positive selection as *F*_st_ had predicted (Supplementary Data: Fig. 2). Our data is in agreement with previous results by other authors^22,77^. As mentioned before, *C. baccatum* is usually cultivated in isolated areas of South America which makes genetic exchanges rare and it has not been subjected to intensive breeding programmes as *C. annuum*. Other works used this statistical tool to successfully detect genomic selective sweeps in *Capsicum* spp. Taitano et al.^30^ reported a purified region on chromosome 6 possibly due to positive selection that confers the phenotype of the Chile de Agua ecotype. Nimmakayala et al.^31^ detected a positive selection for chromosome 4 of *C. baccatum* and low values for the other 11 chromosomes suggesting a neutral selection. Same authors, for *C. annuum*, observed positive values across the entire genome, except for chromosome 8^31^.

## Conclusions

Our study confirms the utility of genomic tools, such as GBS in the identification of highly informative SNPs and its application in the study of the genetic relations between *Capsicum* germplasm accessions. Availability of these tools is of great relevance to *Capsicum* breeders. Here we explore the genetic diversity, genetic structure, and genetic relationships of a collection of Spanish landraces and several foreign controls using a set of 4083 genome-wide SNPs. Population structure seems to be defined mainly by geographic origin and fruit traits. Sweet bell-shaped, blocky or thick-fleshed Spanish landraces and other European varieties located separately from pungent small American accessions. *C. annuum* var. *glabriusculum* seems to have two genetic pools, one closer to *C. annuum* and another closer to *C. chinense* and *C. frutescens*. *C. annuum* accessions have a higher level of positive selection and purified genomic regions according to Tajima’s D statistics and lower diversity among them. This study sheds light on the origin of Spanish landraces origins and their genetic structure and genetic relations, and provides important information for future association studies and for breeding programmes that contribute to the enhancement and protection of these materials.

## Supplementary information

Supplemetary Data - Figures

Readme Supplemenatary information

Supplementary Data: Table 1

Supplementary Data: Table 2

Supplementary Data: Table 3

Supplementary Data: Table 4
