# Supplementary material for: Genetic diversity, population structure, and relationships in a collection of pepper (Capsicum spp.) landraces from the Spanish centre of diversity revealed by genotyping-by-sequencing (GBS)
Source: Hortic Res. 2019 May 1;6:54. doi: 10.1038/s41438-019-0132-8 (PMC6491490; doi:10.1038/s41438-019-0132-8)
Supplement: Supplementary file 1 — Supplemetary Data - Figures [file 41438_2019_132_MOESM1_ESM.pdf]

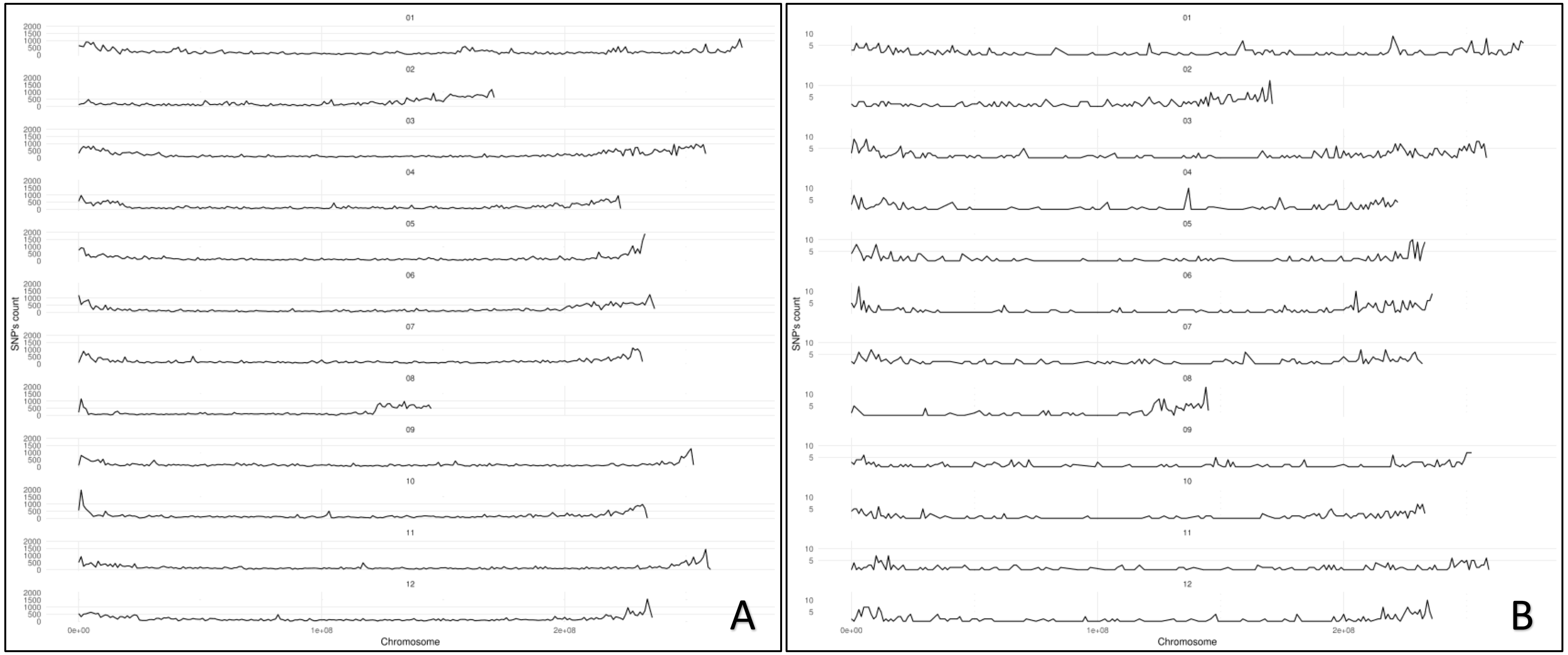

Chromosome	Raw VCF	Filtered VCF
1	56.057	446
2	44.746	332
3	58.705	474
4	42.066	293
5	41.999	320
6	49.735	369
7	41.546	327
8	29.595	232
9	39.779	304
10	41.922	305
11	43.477	333
12	42.053	348
**Total SNP's**	**531.680**	**4.083**

C

Supplementary Data - Figure 1: A) SNPs distribution along the 12 pepper chromosomes for raw (unfiltered) VCF with a total of 531.680 SNPs. **B) S**NPs distribution along the 12 pepper chromosomes for SNPRelate filtered VCF with a total of 4083 SNPs. **C)** Number of SNPs selected per chromosome for both raw (unfiltered) and filtered VCFs.

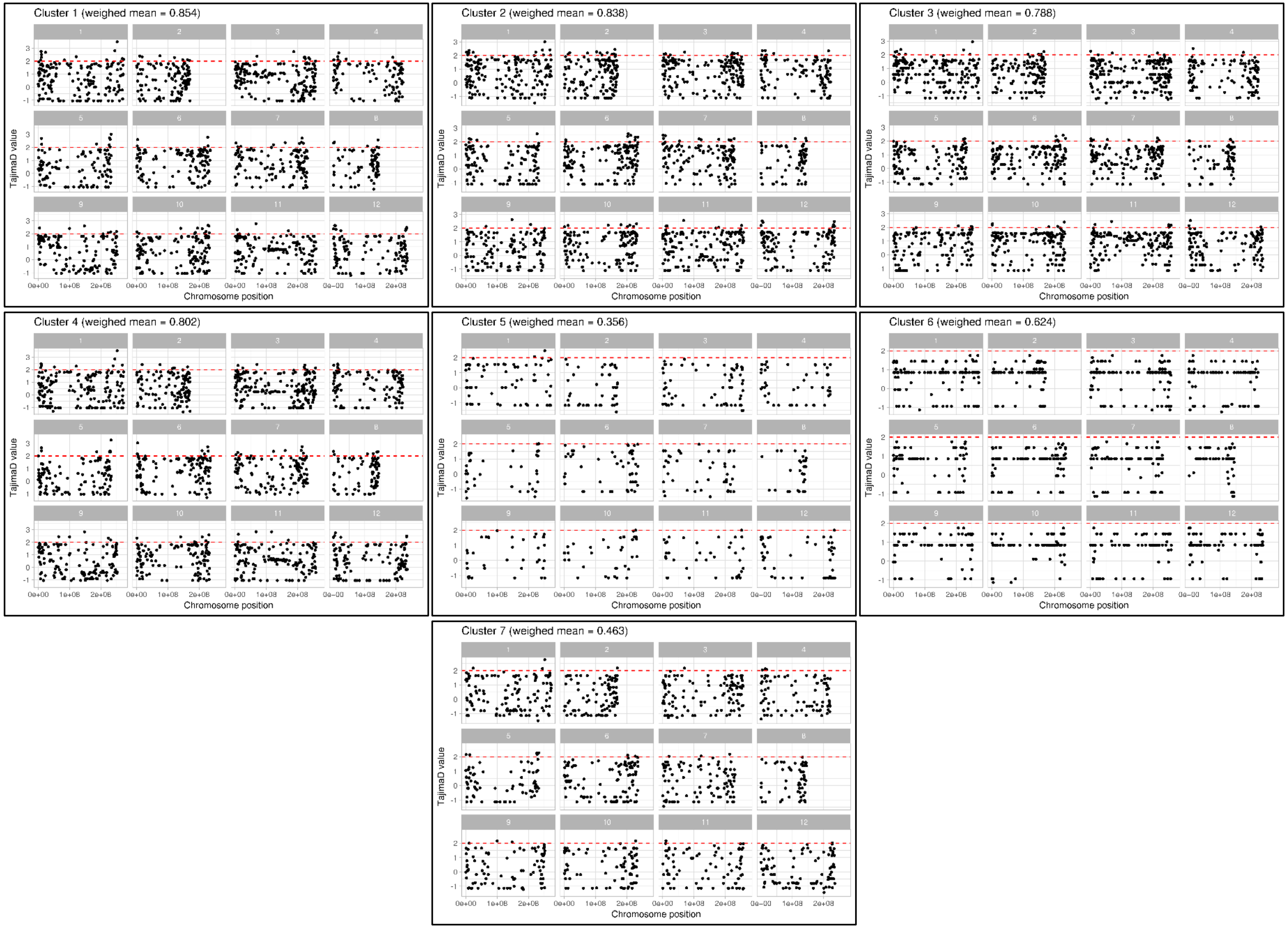

Supplementary Data - Figure 2: Tajima's D values along 12 pepper chromosomes for each DAPC-determined 7 clusters for 4083 segregating sites and correspondent weighed mean. Red dashed line indicates high probability of positive selection.
