# Supplementary material for: Genetic diversity, population structure, and relationships in a collection of pepper (Capsicum spp.) landraces from the Spanish centre of diversity revealed by genotyping-by-sequencing (GBS)
Source: Hortic Res. 2019 May 1;6:54. doi: 10.1038/s41438-019-0132-8 (PMC6491490; doi:10.1038/s41438-019-0132-8)
Supplement: Supplementary file 2 — Readme Supplemenatary information [file 41438_2019_132_MOESM2_ESM.docx]

**Supplementary information**

Supplementary information accompanies the manuscript on the Horticulture Research website <http://www.nature.com/hortres>. It consists of five separate files that are presented as tables and figures that were not included in the original manuscript but provide relevant information to the reader.

The files named as “Supplementary Data – Table X” contain the following information:

**Supplementary Data: Table 1** - List of accessions used in this work and their respective local name, seedbank code, figures abbreviation, origin, provider, cultivar type, fruit type, fruit weight, taste and fruit colour.

**Supplementary Data: Table 2** - Heterozygosity values per accession, species and cultivar type.

**Supplementary Data: Table 3** - List of 4083 highly informative SNPs used for diversity and structure analysis.

**Supplementary Data: Table 4** - BIC values for 148 and 122 accessions subsets and respective DAPC-determined Clusters composition.

The file named “Supplementary Data – Figures” contains the following information:

**Supplementary Data: Figure 1** - **A) S**NPs distribution along the 12 pepper chromosomes for raw (unfiltered) VCF with a total of 531.680 SNPs. **B)** **S**NPs distribution along the 12 pepper chromosomes for SNPRelate filtered VCF with a total of 4083 SNPs. **C)** Number of SNPs selected per chromosome for both raw (unfiltered) and filtered VCFs.

**Supplementary Data: Figure 2** - Tajima’s D values along 12 pepper chromosomes for each DAPC-determined 7 clusters for 4083 segregating sites and correspondent weighed mean. Red dashed line indicates high probability of positive selection.
