# Supplementary material for: Genetic diversity, population structure, and relationships in a collection of pepper (Capsicum spp.) landraces from the Spanish centre of diversity revealed by genotyping-by-sequencing (GBS)
Source: Hortic Res. 2019 May 1;6:54. doi: 10.1038/s41438-019-0132-8 (PMC6491490; doi:10.1038/s41438-019-0132-8)
Supplement: Supplementary file 3 — Supplementary Data: Table 1 [file 41438_2019_132_MOESM3_ESM.pdf]

Abbreviation	Local name (UPV Seedbank code)	Origin (provider)	Cultivar type	Fruit type	Fruit weight (g)	Taste	Fruit colour
	Capsicum annuum var. *annuum*						
bul_kap	Kapiya UV	Bulgaria (Maritsa VCRI)	Traditional	Elongated, Pochard's C2 type	43	Sweet	Red
bul_rat	Bulgarski Ratund	Bulgaria (Maritsa VCRI)	Traditional	Round, Pochard's F type	68	Sweet	Red
bul_siv	Sivriya 600	Bulgaria (Maritsa VCRI)	Traditional	Elongated, Pochard's C2 type	65	Sweet	Red
fra_dll	Doux Long des Landes	France (INRA-GEVES, F. Jourdan)	Traditional	Cayenne, long-sized	23	Sweet	Red
fra_petit	Petit Marseillais	France (INRA-GEVES, F. Jourdan)	Traditional	Blocky, Pochard's A4 type	36	Sweet	Orange
fra_prb	Poivre Rouge de Bresse	France (INRA-GEVES, F. Jourdan)	Traditional	Blocky, small-sized, Pochard's B4 type	9	Sweet	Red
fra_tendre	Tendre de Châteaurenard	France (INRA-GEVES, F. Jourdan)	Traditional	Blocky, Pochard's B1 type	78	Sweet	Red
ind_torp	Torpedo Bangalore	India, Bangalore	Traditional	Cayenne, long-sized	4-5	Hot	Red
ita_carg	Carmagnola Giallo	Italy, Carmagnola, Piedmont	Traditional	Blocky, Pochard's A type	245	Sweet	Yellow
ita_carr	Carmagnola Rosso	Italy, Carmagnola, Piedmont	Traditional	Blocky, Pochard's A type	269	Sweet	Red
ita_cuneo	Peperone Cuneo	Italy, Cuneo	Traditional	Blocky, Pochard's A type	149	Sweet	Yellow
ita_giallo	Cuneo Giallo	Italy (Franchi Sementi)	Commercial heirloom	Blocky, Pochard's A type	171	Sweet	Yellow
ita_senise	di Senise, P.G.I. Peperone di Senise	Italy, Senise, Potenza	Traditional	Elongated, Pochard's C2 type	42	Sweet	Red
ita_top	Topepo Rosso	Italy (Franchi Sementi)	Commercial heirloom	Round, Pochard's F type	58	Sweet	Red
mex_96d	Chile Ancho 1	Mexico, Aguascalientes (UAA)	Traditional	Triangular, Pochard's C4 type	69	Hot	Red
mex_ancho	Chile Ancho 2	Mexico, Aguascalientes (UAA)	Traditional	Triangular, Pochard's C4 type	45	Hot	Red
mex_arb	Chile de Árbol	Mexico	Traditional	Cayenne, short-sized	2 - 3	Hot	Red
mex_mulato	Chile Ancho Mulato	Mexico	Traditional	Triangular, Pochard's C4 type	39	Hot	Brown
mex_pasilla	Pasilla Bajio	Mexico	Traditional	Cayenne, long-sized	23	Hot	Brown
mex_puya	Guacilla Pulla	Mexico, Aguascalientes (UAA)	Traditional	Cayenne, short-sized	3 - 4	Hot	Red
mex_scm	Serrano criollo de Morellos	Mexico	Experimental line	Serrano	4 - 5	Hot	Red
mex_serra	Chile Serrano 1	Mexico	Traditional	Serrano	2 - 3	Hot	Red
mex_serrano	Chile Serrano 2	Mexico	Traditional	Serrano	3	Hot	Red
mu_chile	Chile Ancho 101	Mexico/USA (Reimer Seeds)	Commercial heirloom	Triangular, Pochard's C4 type	45	Hot	Red
mu_espin	Jalapeno Espinalteco	Mexico/USA (P. W. Bosland)	Traditional	Jalapeno	16	Hot	Red
mu_jal	Jalapeno M	Mexico/USA (Reimer Seeds)	Commercial heirloom	Jalapeno	11	Hot	Red
ser_at	Atina	Serbia	Commercial heirloom	Blocky, Pochard's A type	97	Sweet	Red
sp_00057	Pimiento morro de vaca (BGV-57)	Spain, Huesca	Traditional	Blocky, Pochard's A type	90	Sweet	Red
sp_00060	Pimiento morrón de bola (BGV-60)	Spain, Zamora	Traditional	Round, Pochard's F type	114	Sweet	Red
sp_00614	Pimiento morrón de conserva (BGV-614)	Spain, Jaén	Traditional	Round, Pochard's F type	78	Sweet	Red
sp_00637	Pimiento cuatro cascos (BGV-637)	Spain, Galicia	Traditional	Blocky, Pochard's A type	142	Sweet	Red
sp_01319	Pimiento morrón (BGV-1319)	Spain, Asturias	Traditional	Blocky, Pochard's A type	99	Sweet	Red
sp_01814	Pimiento morrón (BGV-1814)	Spain, Tarragona	Traditional	Blocky, Pochard's A type	114	Sweet	Red
sp_01834	Cuatro morros (BGV-1834)	Spain, Barcelona	Traditional	Blocky, Pochard's B type	128	Sweet	Red
sp_01844	Morro de Vedella (BGV-1844)	Spain, Cataluña	Traditional	Blocky, Pochard's B type	152	Sweet	Red
sp_01862	Largo de Reus (BGV-1862)	Spain, Barcelona	Traditional	Blocky, Pochard's B type	120	Sweet	Red
sp_04036	Pimiento gordo de asar (BGV-4036)	Spain, Cáceres	Traditional	Blocky, Pochard's A type	130	Sweet	Red
sp_04322	Pimiento grueso de Murcia (BGV-4322)	Spain, Murcia	Traditional	Blocky, Pochard's A type	163	Sweet	Red
sp_04329	Morro de vaca (BGV-4329)	Spain, Murcia	Traditional	Blocky, Pochard's A type	131	Sweet	Red

sp_04331	Valenciano (BGV-4331)	Spain, Murcia	Traditional	Blocky, Pochard's B type	165	Sweet	Red
sp_04335	Morrón de conserva (BGV-4335)	Spain, Murcia	Traditional	Round, Pochard's F type	108	Sweet	Red
sp_04348	Pimiento trompa de vaca (BGV-4348)	Spain, Murcia	Traditional	Blocky, Pochard's B type	76	Sweet	Red
sp_04349	Pimiento morro de vaca (BGV-4349)	Spain, Murcia	Traditional	Blocky, Pochard's B type	107	Sweet	Red
sp_04507	Pimiento grueso del país (BGV-4507)	Spain, Cantabria	Traditional	Thick flesh, Pochard's C3 type	92	Sweet	Red
sp_05030	Valenciano (BGV-5030)	Spain, Valencia	Traditional	Blocky, Pochard's B type	153	Sweet	Red
sp_05041	Morrón de conserva (BGV-5041)	Spain, Valencia	Traditional	Round, Pochard's F type	130	Sweet	Red
sp_05057	Pimiento cuatro cantos (BGV-5057)	Spain, Castellón	Traditional	Blocky, Pochard's A type	148	Sweet	Red
sp_05083	Pimiento gordo (BGV-5083)	Spain, Castellón	Traditional	Blocky, Pochard's A type	172	Sweet	Red
sp_05103	Valenciano (BGV-5103)	Spain, Valencia	Traditional	Blocky, Pochard's B type	192	Sweet	Red
sp_05109	Trompa de vaca (BGV-5109)	Spain, Alicante	Traditional	Blocky, Pochard's A type	149	Sweet	Red
sp_05113	Valenciano (BGV-5113)	Spain, Alicante	Traditional	Blocky, Pochard's B type	286	Sweet	Red
sp_05114	Morrón de conserva (BGV-5114)	Spain, Alicante	Traditional	Round, Pochard's F type	74	Sweet	Red
sp_05121	Valenciano (BGV-5121)	Spain, Alicante	Traditional	Blocky, Pochard's B type	285	Sweet	Red
sp_05126	Valenciano (BGV-5126)	Spain, Alicante	Traditional	Blocky, Pochard's B type	116	Sweet	Red
sp_10183	Pimiento de Pico (BGV-10183)	Spain, Navarra	Traditional	Triangular, Pochard's C4 type	53	Sweet	Red
sp_10185	Pimiento de Padrón (BGV-10185)	Spain, La Coruña	Traditional	Blocky small-sized, Pochard's B4 type	13	Hot	Red
sp_10186	Pimiento del Piquillo (BGV-10186)	Spain, Navarra	Traditional	Triangular, Pochard's C4 type	32	Sweet	Red
sp_10368	Pimiento de Infantes (BGV-10368)	Spain, Ciudad Real	Traditional	Blocky, Pochard's A type	195	Sweet	Red
sp_10447	Pimiento del País (BGV-10447)	Spain, Islas Baleares	Traditional	Round, Pochard's F type	118	Sweet	Red
sp_10451	Pimiento Najerano gordo (BGV-10451)	Spain, La Rioja	Traditional	Thick flesh, Pochard's C3 type	144	Sweet	Red
sp_10540	Pimiento de casco (BGV-10540)	Spain, Albacete	Traditional	Blocky, Pochard's A type	113	Sweet	Red
sp_10582	Valenciano (BGV-10582)	Spain, Valencia	Traditional	Blocky, Pochard's A type	147	Sweet	Red
sp_10599	Pimiento cuatro morros (BGV-10599)	Spain, León	Traditional	Blocky, Pochard's A type	112	Sweet	Red
sp_10600	Largo de Reus (BGV-10600)	Spain, Tarragona	Traditional	Blocky, Pochard's B type	102	Sweet	Red
sp_10946	Morrón de cuatro Picos (BGV-10946)	Spain, Asturias	Traditional	Blocky, Pochard's A type	127	Sweet	Red
sp_11038	Pimiento morro de vaca (BGV-11038)	Spain, Albacete	Traditional	Blocky, Pochard's A type	180	Sweet	Red
sp_11092	Pimiento gordo najerano (BGV-11092)	Spain, La Rioja	Traditional	Thick flesh, Pochard's C3 type	99	Sweet	Red
sp_11205	Pimiento de Padrón (BGV-11205)	Spain, Navarra	Traditional	Blocky small-sized, Pochard's B4 type	20	Sweet	Red
sp_11213	Pimiento de cuatro morros (BGV-11213)	Spain, Cantabria	Traditional	Blocky, Pochard's A type	137	Sweet	Red
sp_11267	Pimiento morrón largo (BGV-11267)	Spain, León	Traditional	Blocky, Pochard's A type	116	Sweet	Red
sp_11500	Lora (BGV-11500)	Spain, León	Traditional	Round, Pochard's F type	103	Sweet	Red
sp_11528	Morrón de Loyola cuatro cantos (BGV-11528)	Spain, Guipúzcoa	Traditional	Blocky, Pochard's A type	140	Sweet	Red
sp_11531	Guindilla (BGV-11531)	Spain, Guipúzcoa	Traditional	Cayenne, long-sized	8	Hot	Red
sp_11558	Pimiento gordo de ensalada (BGV-11558)	Spain, Cáceres	Traditional	Blocky, Pochard's A type	141	Sweet	Red
sp_11630	Pimiento morrón gordo (BGV-11630)	Spain, Vizcaya	Traditional	Blocky, Pochard's A type	125	Sweet	Red
sp_11751	Pimiento gordo morro de vaca (BGV-11751)	Spain, Huesca	Traditional	Blocky, Pochard's B type	138	Sweet	Red
sp_11814	Dulce italiano (BGV-11814)	Spain, León	Traditional	Elongated, Pochard's C2 type	85	Sweet	Red
sp_11881	Pimiento morrón de conserva (BGV-11881)	Spain, Guadalajara	Traditional	Round, Pochard's F type	112	Sweet	Red
sp_13004	Pimiento de asar gordo najerano (BGV-13004)	Spain, Vizcaya	Traditional	Thick flesh, Pochard's C3 type	123	Sweet	Red
sp_13009	Pimiento de asar mucha carne (BGV-13009)	Spain, Vizcaya	Traditional	Thick flesh, Pochard's C3 type	112	Sweet	Red

sp_13636	Pimiento gordo (BGV-13636)	Spain, Salamanca	Traditional	Blocky, Pochard's A type	115	Sweet	Red
sp_13638	Pimiento gordo (BGV-13638)	Spain, Zamora	Traditional	Blocky, Pochard's B type	140	Sweet	Red
sp_arnoia	Arnoia, P.G.I. Pemento da Arnoia	Spain, Galicia, Orense	Traditional	Blocky, Pochard's A type	62	Sweet	Red
sp_bier	Bierzo, Cons. Reg. P.G.I. Pimiento Asado Bierzo	Spain, León	Traditional	Thick flesh, Pochard's C3 type	109	Sweet	Red
sp_bola	Pimiento de Bola, Cons. Reg. P.D.O. Pimentón Murcia	Spain, Murcia	Traditional	Round, Pochard's N type	18	Sweet	Red
sp_cala	Calahorra	Spain, La Rioja	Traditional	Round, Pochard's F type	96	Sweet	Red
sp_cat	California Wonder Catedral	Spain (Zeraim Ibérica)	Commercial F1	Blocky, Pochard's A type	120	Sweet	Red
sp_cwr	California Wonder red	Spain (COMAV)	Experimental line	Blocky, Pochard's A type	115	Sweet	Red
sp_cwy	California Wonder yellow	Spain (COMAV)	Experimental line	Blocky, Pochard's A type	133	Sweet	Yellow
sp_fresno	Morrón de Fresno de la Vega y Benavente P.G.I.	Spain, León and Zamora	Traditional	Blocky, Pochard's A type	125	Sweet	Red
sp_guer	Guernika cv. Derio, P.G.I. Gernikako Piperra	Spain, País Vasco (S. Larregla)	Traditional	Elongated, Pochard's C2 type	19	Sweet	Red
sp_ibarra	Guindilla de Ibarra	Spain, País Vasco (S. Larregla)	Traditional	Cayenne, long-sized	6	Hot	Red
sp_inf	De Infantes	Spain (Mascarell Seeds)	Commercial heirloom	Blocky, Pochard's A type	201	Sweet	Red
sp_ital	Italiano	Spain (Intersemillas)	Commercial F1	Elongated, Pochard's C1 type	85	Sweet	Red
sp_jal	Jalapeno	Spain, Valencia (COMAV)	Experimental line	Jalapeno	110	Hot	Red
sp_lamr	Lamuyo	Spain (Intersemillas)	Commercial F1	Blocky, Pochard's B type	118	Sweet	Red
sp_lamy	Lamuyo	Spain (Intersemillas)	Commercial F1	Blocky, Pochard's B type	124	Sweet	Yellow
sp_mojo	Mojo Palmero	Spain, La Palma	Traditional	Triangular, Pochard's C4 type	18	Hot	Red
sp_naj	Najerano	Spain (Ramiro Arnedo)	Commercial heirloom	Thick flesh, Pochard's C3 type	111	Sweet	Red
sp_pad	Pimiento de Padrón	Spain, Galicia	Traditional	Blocky small-sized, Pochard's B4 type	14	Sweet	Red
sp_pascual	Guindilla Pascual	Spain, Valencia	Traditional	Cayenne, long-sized	7	Hot	Red
sp_pic	Picante Largo	Spain (Intersemillas)	Commercial F1	Elongated, Pochard's C1 type	7	Hot	Red
sp_piq	Pimiento del Piquillo, Cons. Reg. P.D.O. Pimiento Piquillo de Lodosa	Spain, Navarra	Traditional	Triangular, Pochard's C4 type	29	Sweet	Red
sp_reus	Largo de Reus	Spain (Batlle)	Commercial heirloom	Blocky, Pochard's B type	238	Sweet	Red
sp_rsw	RSW	Spain, Valencia (COMAV)	Traditional	Cayenne, short-sized	4 - 5	Hot	Red
sp_vlc	Valenciano	Spain, Valencia	Traditional	Blocky, Pochard's B type	151	Sweet	Red
sri_ka	Ka 2	Sri Lanka	Commercial heirloom	Cayenne, short-sized	3	Hot	Red
tur_aci	Ací Sivri	Turkey	Traditional	Cayenne, long-sized	13	Hot	Red
usa_13293	Numex Sandia (BGV-13293)	USA, New Mexico	Traditional	Elongated, Pochard's C2 type	62	Hot	Red
usa_64	Numex 6-4	USA, New Mexico	Traditional	Elongated, Pochard's C2 type	64	Hot	Red
usa_cand	Jalapeno Candelaria	USA, New Mexico (P. W. Bosland)	Traditional	Jalapeno	21	Hot	Red
usa_chima	Chimayó 1	USA, New Mexico	Traditional	Blocky small-sized, Pochard's B4 type	18	Hot	Red
usa_chimayo	Chimayó 2	USA, New Mexico (P. W. Bosland)	Traditional	Blocky small-sized, Pochard's B4 type	19	Hot	Red
usa_conq	Numex Conquistador	USA, New Mexico	Traditional	Elongated, Pochard's C2 type	53	Sweet	Red
usa_jap	Chile Japonés	USA, New Mexico	Traditional	Cayenne, very short-sized	1 - 2	Hot	Red
usa_jim	Numex Big Jim	USA, New Mexico (P. W. Bosland)	Traditional	Elongated, Pochard's C2 type	53	Hot	Red
usa_lrc	Louisiana Red Cayenne	USA, Louisiana (Reimer Seeds)	Commercial heirloom	Cayenne, long-sized	16	Hot	Red
usa_num	Numex	USA, New Mexico	Traditional	Elongated, Pochard's C2 type	45	Hot	Red

	Capsicum annuum var. *glabriusculum*						
mex_c1333	C1333	Mexico, Chiapas (UAA)	Wild type	Round, small-sized, Pochard's N type	< 1	Hot	Red
mex_n1411	N1411	Mexico, Nayanit (UAA)	Wild type	Cayenne, very short-sized	< 1	Hot	Red
mex_o1430	O1430	Mexico, Oaxaca (UAA)	Wild type	Round, small-sized, Pochard's N type	< 1	Hot	Red
mex_q1078	Q1078	Mexico, Queretaro (UAA)	Wild type	Round, small-sized, Pochard's N type	< 1	Hot	Red
mex_s1120	S1120	Mexico, Sonora (UAA)	Wild type	Round, small-sized, Pochard's N type	< 1	Hot	Red
mex_v1196	V1196	Mexico, Veracruz (UAA)	Wild type	Cayenne, very short-sized	< 1	Hot	Brown
usa_a1003	A1003	USA, Arizona (UAA)	Wild type	Round, small-sized, Pochard's N type	< 1	Hot	Red
	Capsicum baccatum						
bol_ari	Arivivi (Bol - 154)	Bolivia, Santa Cruz	Wild type	Cayenne, very short-sized	1 - 2	Hot	Orange
bol_toro	Asta de Toro (Bol - 058)	Bolivia, Cochabamba	Traditional	Triangular, small-sized	5	Hot	Red
bol_037	Bol - 037	Bolivia, Chuquisaca	Traditional	Triangular, small-sized, thin flesh	3 - 4	Hot	Yellow
bol_039	Bol - 039	Bolivia, Chuquisaca	Traditional	Triangular, small-sized, thin flesh	27	Hot	Red
bol_103	Bol - 103	Bolivia, Santa Cruz	Wild type	Cayenne, short-sized	1 - 2	Hot	Red
bol_120	Bol - 120	Bolivia, Santa Cruz	Traditional	Round, small-sized, Pochard's N type	4 - 5	Hot	Red
bol_174	Bol - 174	Bolivia, Chuquisaca	Traditional	Cayenne, short-sized	22	Hot	Red
bol_175	Bol - 175	Bolivia, Chuquisaca	Traditional	Cayenne, short-sized	9	Hot	Red
bol_178	Bol - 178	Bolivia, Chuquisaca	Traditional	Cayenne, long-sized	13	Hot	Red
	Capsicum chinense						
bol_198	Bol - 198	Bolivia, Santa Cruz	Traditional	Round, small-sized, Pochard's N type	13	Hot	Orange
eq_973	ECU - 973	Equador, Napo	Traditional	Triangular, small-sized, thin flesh	2 - 3	Hot	Red
eq_994	ECU - 994	Equador	Traditional	Triangular, small-sized	3 - 4	Hot	Red
peru_cabra	Cacho de Cabra	Peru, Lambayeque	Traditional	Triangular, small-sized, thin flesh	12	Hot	Orange
peru_cer	Ají Cerezo	Peru, Lambayeque	Traditional	Flat round, small-sized	16	Hot	Red
peru_char	Charapita	Peru, Amazonía	Traditional	Round, small-sized, Pochard's N type	< 1	Hot	Orange
peru_limo	Ají Limo	Peru, Piura	Traditional	Triangular, small-sized, thin flesh	10	Hot	Orange
peru_mis	Aji Miscucho	Peru, La Libertad	Traditional	Triangular, small-sized, thin flesh	8	Hot	Yellow
peru_moche	Ají Mochero	Peru, Valle de Moche	Traditional	Cayenne, short-sized	4 - 5	Hot	Yellow
usa_hab	Habanero	USA (Penn State University)	Traditional	Triangular, small-sized, thin flesh	10	Hot	Red
usa_pi	PI - 152225	USA (USDA)	Experimental line	Triangular, small-sized, thin flesh	6	Hot	Brown
ven_dulce	Ají Dulce	Venezuela	Traditional	Flat round, small-sized	3 - 4	Hot	Red
	Capsicum frutescens						
bol_144	Bol - 144	Bolivia, Santa Cruz	Traditional	Cayenne, very short-sized	<1	Hot	Red
ven_chi	Ají Chirere	Venezuela	Traditional	Cayenne, very short-sized	< 1	Hot	Red
