# Supplementary material for: Genetic diversity, population structure, and relationships in a collection of pepper (Capsicum spp.) landraces from the Spanish centre of diversity revealed by genotyping-by-sequencing (GBS)
Source: Hortic Res. 2019 May 1;6:54. doi: 10.1038/s41438-019-0132-8 (PMC6491490; doi:10.1038/s41438-019-0132-8)
Supplement: Supplementary file 4 — Supplementary Data: Table 2 [file 41438_2019_132_MOESM4_ESM.pdf]

Abbreviation	Local name (UPV Seedbank code)	Cultivar type	O(Hom)	E(Hom)	Number of sites	F	Hom (%)	Het (%)
	Capsicum annuum var. *annuum*							
bul_kap	Kapiya UV	Traditional	265907	237011,3	272973	0,80	97,41	2,59
bul_rat	Bulgarski Ratund	Traditional	280992	250107,2	288788	0,80	97,30	2,70
bul_siv	Sivriya 600	Traditional	267554	238417,7	274835	0,80	97,35	2,65
fra_dll	Doux Long des Landes	Traditional	278785	248239,6	286612	0,80	97,27	2,73
fra_petit	Petit Marseillais	Traditional	248036	221853,3	254925	0,79	97,30	2,70
fra_prb	Poivre Rouge de Bresse	Traditional	254627	227006,8	261594	0,80	97,34	2,66
fra_tendre	Tendre de Châteaurenard	Traditional	259137	231541,4	266391	0,79	97,28	2,72
ind_torp	Torpedo Bangalore	Traditional	245287	220225,2	253416	0,76	96,79	3,21
ita_carg	Carmagnola Giallo	Traditional	299336	265721,2	308235	0,79	97,11	2,89
ita_carr	Carmagnola Rosso	Traditional	280678	249630,4	288484	0,80	97,29	2,71
ita_cuneo	Peperone Cuneo	Traditional	257472	229905,4	264679	0,79	97,28	2,72
ita_giallo	Cuneo Giallo	Commercial heirloom	299521	266722,4	308909	0,78	96,96	3,04
ita_senise	di Senise, P.G.I. Peperone di Senise	Traditional	260193	231827,5	267204	0,80	97,38	2,62
ita_top	Topepo Rosso	Commercial heirloom	259619	232360,7	267243	0,78	97,15	2,85
mex_96d	Chile Ancho 1	Traditional	227987	204563,1	235385	0,76	96,86	3,14
mex_ancho	Chile Ancho 2	Traditional	306957	275871,2	320860	0,69	95,67	4,33
mex_arb	Chile de Árbol	Traditional	255119	228324,4	263831	0,75	96,70	3,30
mex_mulato	Chile Ancho Mulato	Traditional	261507	235465,6	271192	0,73	96,43	3,57
mex_pasilla	Pasilla Bajio	Traditional	281343	250210,4	289456	0,79	97,20	2,80
mex_puya	Guacilla Pulla	Traditional	296579	264567,2	307424	0,75	96,47	3,53
mex_scm	Serrano criollo de Morellos	Experimental line	267010	236872,9	273887	0,81	97,49	2,51
mex_serra	Chile Serrano 1	Traditional	256454	228382,2	263532	0,80	97,31	2,69
mex_serrano	Chile Serrano 2	Traditional	293450	260949,1	302989	0,77	96,85	3,15
mu_chile	Chile Ancho 101	Commercial heirloom	245963	219832,3	252865	0,79	97,27	2,73
mu_espin	Jalapeno Espinalteco	Traditional	260522	232069,6	267997	0,79	97,21	2,79
mu_jal	Jalapeno M	Commercial heirloom	262011	234425,3	270470	0,77	96,87	3,13
ser_at	Atina	Commercial heirloom	284397	252735,5	292217	0,80	97,32	2,68
sp_00057	Pimiento morro de vaca (BGV-57)	Traditional	312320	277283,7	322065	0,78	96,97	3,03
sp_00060	Pimiento morrón de bola (BGV-60)	Traditional	294779	262032,3	303661	0,79	97,08	2,92
sp_00614	Pimiento morrón de conserva (BGV-614)	Traditional	275930	246152,1	283834	0,79	97,22	2,78
sp_00637	Pimiento cuatro cascos (BGV-637)	Traditional	271846	245726,4	283442	0,69	95,91	4,09
sp_01319	Pimiento morrón (BGV-1319)	Traditional	292213	260322,5	301184	0,78	97,02	2,98
sp_01814	Pimiento morrón (BGV-1814)	Traditional	274721	246173,1	284056	0,75	96,71	3,29
sp_01834	Cuatro morros (BGV-1834)	Traditional	276200	246346,6	284524	0,78	97,07	2,93
sp_01844	Morro de Vedella (BGV-1844)	Traditional	266484	238849,4	274923	0,77	96,93	3,07
sp_01862	Largo de Reus (BGV-1862)	Traditional	302748	269429,5	312325	0,78	96,93	3,07
sp_04036	Pimiento gordo de asar (BGV-4036)	Traditional	247537	221617,2	254535	0,79	97,25	2,75
sp_04322	Pimiento grueso de Murcia (BGV-4322)	Traditional	266880	237981	274549	0,79	97,21	2,79
sp_04329	Morro de vaca (BGV-4329)	Traditional	261847	234569,3	269860	0,77	97,03	2,97
sp_04331	Valenciano (BGV-4331)	Traditional	275257	245359,3	283306	0,79	97,16	2,84
sp_04335	Morrón de conserva (BGV-4335)	Traditional	305412	270931,4	314440	0,79	97,13	2,87
sp_04348	Pimiento trompa de vaca (BGV-4348)	Traditional	246602	220760,7	253525	0,79	97,27	2,73
sp_04349	Pimiento morro de vaca (BGV-4349)	Traditional	280945	250232,1	289097	0,79	97,18	2,82
sp_04507	Pimiento grueso del país (BGV-4507)	Traditional	244437	221253,3	254268	0,70	96,13	3,87
sp_05030	Valenciano (BGV-5030)	Traditional	282600	251816,1	290618	0,79	97,24	2,76
sp_05041	Morrón de conserva (BGV-5041)	Traditional	275480	245921,9	283731	0,78	97,09	2,91
sp_05057	Pimiento cuatro cantos (BGV-5057)	Traditional	285208	254379,3	294345	0,77	96,90	3,10
sp_05083	Pimiento gordo (BGV-5083)	Traditional	271507	243124,6	280175	0,77	96,91	3,09
sp_05103	Valenciano (BGV-5103)	Traditional	163862	147276,9	167800	0,81	97,65	2,35
sp_05109	Trompa de vaca (BGV-5109)	Traditional	299963	266723,4	309178	0,78	97,02	2,98
sp_05113	Valenciano (BGV-5113)	Traditional	299455	269500,3	312277	0,70	95,89	4,11
sp_05114	Morrón de conserva (BGV-5114)	Traditional	287846	257215,9	297038	0,77	96,91	3,09
sp_05121	Valenciano (BGV-5121)	Traditional	270086	241274,6	278070	0,78	97,13	2,87
sp_05126	Valenciano (BGV-5126)	Traditional	290068	258216	298471	0,79	97,18	2,82
sp_10183	Pimiento de Pico (BGV-10183)	Traditional	257715	230306,8	265117	0,79	97,21	2,79
sp_10185	Pimiento de Padrón (BGV-10185)	Traditional	283489	252595,9	292046	0,78	97,07	2,93
sp_10186	Pimiento del Piquillo (BGV-10186)	Traditional	284530	253120,6	292791	0,79	97,18	2,82

sp_10368	Pimiento de Infantes (BGV-10368)	Traditional	250944	224574	258521	0,78	97,07	2,93
sp_10447	Pimiento del País (BGV-10447)	Traditional	273299	244181,7	281355	0,78	97,14	2,86
sp_10451	Pimiento Najerano gordo (BGV-10451)	Traditional	281264	251164,9	289837	0,78	97,04	2,96
sp_10540	Pimiento de casco (BGV-10540)	Traditional	261955	234885	270276	0,76	96,92	3,08
sp_10582	Valenciano (BGV-10582)	Traditional	264816	237368,6	273053	0,77	96,98	3,02
sp_10599	Pimiento cuatro morros (BGV-10599)	Traditional	269918	241515,1	278385	0,77	96,96	3,04
sp_10600	Largo de Reus (BGV-10600)	Traditional	264719	236334,3	272292	0,79	97,22	2,78
sp_10946	Morrón de cuatro Picos (BGV-10946)	Traditional	273216	244218,9	281742	0,77	96,97	3,03
sp_11038	Pimiento morro de vaca (BGV-11038)	Traditional	256010	228414,3	263086	0,80	97,31	2,69
sp_11092	Pimiento gordo najerano (BGV-11092)	Traditional	262008	234437,3	269893	0,78	97,08	2,92
sp_11205	Pimiento de Padrón (BGV-11205)	Traditional	305861	271520,3	315299	0,78	97,01	2,99
sp_11213	Pimiento de cuatro morros (BGV-11213)	Traditional	289789	257580,1	298095	0,79	97,21	2,79
sp_11267	Pimiento morrón largo (BGV-11267)	Traditional	209923	188619,2	215849	0,78	97,25	2,75
sp_11500	Lora (BGV-11500)	Traditional	301334	267756,6	310555	0,78	97,03	2,97
sp_11528	Morrón de Loyola cuatro cantos (BGV-11528)	Traditional	303751	270168,8	313058	0,78	97,03	2,97
sp_11531	Guindilla (BGV-11531)	Traditional	272508	243084,2	280710	0,78	97,08	2,92
sp_11558	Pimiento gordo de ensalada (BGV-11558)	Traditional	217364	194784,2	223188	0,79	97,39	2,61
sp_11630	Pimiento morrón gordo (BGV-11630)	Traditional	277584	248146,7	286292	0,77	96,96	3,04
sp_11751	Pimiento gordo morro de vaca (BGV-11751)	Traditional	243672	218580,1	250975	0,77	97,09	2,91
sp_11814	Dulce italiano (BGV-11814)	Traditional	232524	208286,7	238714	0,80	97,41	2,59
sp_11881	Pimiento morrón de conserva (BGV-11881)	Traditional	287818	256229,6	296718	0,78	97,00	3,00
sp_13004	Pimiento de asar gordo najerano (BGV-13004)	Traditional	229599	206084,3	236276	0,78	97,17	2,83
sp_13009	Pimiento de asar mucha carne (BGV-13009)	Traditional	275339	246024	283696	0,78	97,05	2,95
sp_13636	Pimiento gordo (BGV-13636)	Traditional	272878	243400,1	280696	0,79	97,21	2,79
sp_13638	Pimiento gordo (BGV-13638)	Traditional	297162	264627,6	306403	0,78	96,98	3,02
sp_arnoia	Arnoia, P.G.I. Pemento da Arnoia	Traditional	261518	233740,7	269195	0,78	97,15	2,85
sp_bier	Bierzo, Cons. Reg. P.G.I. Pimiento Asado Bierzo	Traditional	244358	218942,1	251334	0,78	97,22	2,78
sp_bola	Pimiento de Bola, Cons. Reg. P.D.O. Pimentón Murcia	Traditional	262756	233808,5	269691	0,81	97,43	2,57
sp_cala	Calahorra	Traditional	278109	247869,6	285946	0,79	97,26	2,74
sp_cat	California Wonder Catedral	Commercial F1	231597	208027,8	238773	0,77	96,99	3,01
sp_cwr	California Wonder red	Experimental line	280601	250289,7	289192	0,78	97,03	2,97
sp_cwy	California Wonder yellow	Experimental line	283479	252205,6	291461	0,80	97,26	2,74
sp_fresno	Morrón de Fresno de la Vega y Benavente P.G.I.	Traditional	274308	244854,7	282329	0,79	97,16	2,84
sp_guer	Guernika cv. Derio, P.G.I. Gernikako Piperra	Traditional	290581	258183,4	299056	0,79	97,17	2,83
sp_ibarra	Guindilla de Ibarra	Traditional	267129	237990,1	274302	0,80	97,38	2,62
sp_inf	De Infantes	Commercial heirloom	260306	232767,6	267856	0,78	97,18	2,82
sp_ital	Italiano	Commercial F1	279784	253309,6	292303	0,68	95,72	4,28
sp_jal	Jalapeno	Experimental line	275171	246967,8	285715	0,73	96,31	3,69
sp_lamr	Lamuyo	Commercial F1	314289	282617,5	328259	0,69	95,74	4,26
sp_lamy	Lamuyo	Commercial F1	261314	235785,5	271333	0,72	96,31	3,69
sp_mojo	Mojo Palmero	Traditional	298491	264596,9	307001	0,80	97,23	2,77
sp_naj	Najerano	Commercial heirloom	291272	259279	300459	0,78	96,94	3,06
sp_pad	Pimiento de Padrón	Traditional	270128	240725,6	277606	0,80	97,31	2,69
sp_pascual	Guindilla Pascual	Traditional	255620	227887,2	262769	0,80	97,28	2,72
sp_pic	Picante Largo	Commercial F1	273207	250176,5	289123	0,59	94,50	5,50
sp_piq	Pimiento del Piquillo, Cons. Reg. P.D.O. Pimiento Piquillo de Lodosa	Traditional	282167	250354,3	289960	0,80	97,31	2,69
sp_reus	Largo de Reus	Commercial heirloom	244216	218803,8	251000	0,79	97,30	2,70
sp_rsw	RSW	Traditional	235776	210793	242499	0,79	97,23	2,77
sp_vlc	Valenciano	Traditional	293355	260889,1	301858	0,79	97,18	2,82
sri_ka	Ka 2	Commercial heirloom	256582	228240,3	263412	0,81	97,41	2,59
tur_aci	Ací Sivri	Traditional	280148	249098,7	288246	0,79	97,19	2,81
usa_13293	Numex Sandia (BGV-13293)	Traditional	257413	229226,5	264291	0,80	97,40	2,60
usa_64	Numex 6-4	Traditional	277092	246174,9	284807	0,80	97,29	2,71
usa_cand	Jalapeno Candelaria	Traditional	254410	227201,4	262075	0,78	97,08	2,92
usa_chima	Chimayó 1	Traditional	303447	268805,4	312438	0,79	97,12	2,88
usa_chimayo	Chimayó 2	Traditional	245189	219366,3	252454	0,78	97,12	2,88
usa_conq	Numex Conquistador	Traditional	240054	213964,5	246226	0,81	97,49	2,51
usa_jap	Chile Japonés	Traditional	283030	250623,6	290876	0,81	97,30	2,70
usa_jim	Numex Big Jim	Traditional	234975	209848,7	241104	0,80	97,46	2,54
usa_lrc	Louisiana Red Cayenne	Commercial heirloom	259347	234132	270213	0,70	95,98	4,02

usa_num	Numex	Traditional	278672	247328,3	286089	0,81	97,41	2,59
	Capsicum annuum var. *glabriusculum*							
mex_c1333	C1333	Wild type	207825	184284	214676	0,77	96,81	3,19
mex_n1411	N1411	Wild type	230063	203739,2	237963	0,77	96,68	3,32
mex_o1430	O1430	Wild type	243197	214907,6	251447	0,77	96,72	3,28
mex_q1078	Q1078	Wild type	252037	223508,5	258838	0,81	97,37	2,63
mex_s1120	S1120	Wild type	255637	226083,7	262450	0,81	97,40	2,60
mex_v1196	V1196	Wild type	214102	191040,7	219702	0,80	97,45	2,55
usa_a1003	A1003	Wild type	242189	214819,2	248512	0,81	97,46	2,54
	Capsicum baccatum							
bol_ari	Arivivi (Bol - 154)	Wild type	195546	174580,5	205854	0,67	94,99	5,01
bol_toro	Asta de Toro (Bol - 058)	Traditional	193368	173561,6	204562	0,64	94,53	5,47
bol_037	Bol - 037	Traditional	194939	173883,2	204783	0,68	95,19	4,81
bol_039	Bol - 039	Traditional	210119	188549,7	222696	0,63	94,35	5,65
bol_103	Bol - 103	Wild type	194735	173740,2	204858	0,67	95,06	4,94
bol_120	Bol - 120	Traditional	198889	178106,5	210172	0,65	94,63	5,37
bol_174	Bol - 174	Traditional	201619	180081,3	212532	0,66	94,87	5,13
bol_175	Bol - 175	Traditional	211455	188984,7	223489	0,65	94,62	5,38
bol_178	Bol - 178	Traditional	195260	174336,2	205613	0,67	94,96	5,04
	Capsicum chinense							
bol_198	Bol - 198	Traditional	204423	180820,8	211091	0,78	96,84	3,16
eq_973	ECU - 973	Traditional	225302	199144,1	233341	0,76	96,55	3,45
eq_994	ECU - 994	Traditional	196455	173877,2	203032	0,77	96,76	3,24
peru_cabra	Cacho de Cabra	Traditional	208122	184453,6	215523	0,76	96,57	3,43
peru_cer	Ají Cerezo	Traditional	232219	204977,9	240461	0,77	96,57	3,43
peru_char	Charapita	Traditional	191840	170312,2	198467	0,76	96,66	3,34
peru_limo	Ají Limo	Traditional	208128	184706	215795	0,75	96,45	3,55
peru_mis	Aji Miscucho	Traditional	220046	194859,5	228317	0,75	96,38	3,62
peru_moche	Ají Mochero	Traditional	207100	183956,6	214884	0,75	96,38	3,62
usa_hab	Habanero	Traditional	219517	193933,8	226951	0,77	96,72	3,28
usa_pi	PI - 152225	Experimental line	220713	195206	228902	0,76	96,42	3,58
ven_dulce	Ají Dulce	Traditional	231738	204402	239722	0,77	96,67	3,33
	Capsicum frutescens							
bol_144	Bol - 144	Traditional	202848	180156,7	209422	0,78	96,86	3,14
ven_chi	Ají Chirere	Traditional	210684	193188,5	225330	0,54	93,50	6,50

Species	Number of Individuals	Minimum	Maximum	Mean
Capsicum annuum var. annuum	118	2,35	5,50	2,97
Capsicum annuum var. glabriusculum	7	2,54	3,32	2,87
Capsicum baccatum	9	4,81	5,65	5,20
Capsicum chinense	12	3,16	3,62	3,42
Capsicum frutescens	2	3,14	6,50	4,82
**Cultivar type**				
Commercial F1	5	3,01	5,50	4,15
Commercial heirloom	10	2,59	4,02	2,96
Experimental line	5	2,51	3,69	3,10
Traditional	119	2,35	6,50	3,12
Wild type	9	2,54	5,01	3,34
**Overall values**	148	2,35	6,50	3,16
