# Supplementary material for: Genetic diversity, population structure, and relationships in a collection of pepper (Capsicum spp.) landraces from the Spanish centre of diversity revealed by genotyping-by-sequencing (GBS)
Source: Hortic Res. 2019 May 1;6:54. doi: 10.1038/s41438-019-0132-8 (PMC6491490; doi:10.1038/s41438-019-0132-8)
Supplement: Supplementary file 5 — Supplementary Data: Table 3 [file 41438_2019_132_MOESM5_ESM.pdf]

SNP Number	Genome Position
1	S1_67744
2	S1_322371
3	S1_441966
4	S1_510900
5	S1_830812
6	S1_1061430
7	S1_1523202
8	S1_1569724
9	S1_1783499
10	S1_2129165
11	S1_2218953
12	S1_2460791
13	S1_2875909
14	S1_2885988
15	S1_3126751
16	S1_3490621
17	S1_3844188
18	S1_3995509
19	S1_4052752
20	S1_4409375
21	S1_4903196
22	S1_4997173
23	S1_5039504
24	S1_5264229
25	S1_5584660
26	S1_6003748
27	S1_6114261
28	S1_6174401
29	S1_6358632
30	S1_6361710
31	S1_6689194
32	S1_7232051
33	S1_7430489
34	S1_7808546
35	S1_8152176
36	S1_8152187
37	S1_8747440
38	S1_9267867
39	S1_9479933
40	S1_9479941
41	S1_9899861
42	S1_9923206
43	S1_10081111
44	S1_10334283
45	S1_10716337
46	S1_10716486
47	S1_10824477
48	S1_10824513
49	S1_10824527
50	S1_11129658
51	S1_11618709
52	S1_12237790
53	S1_12675884
54	S1_12867266
55	S1_13634354
56	S1_13939547
57	S1_14221211
58	S1_14512036
59	S1_14645765
60	S1_14766522
61	S1_15007391
62	S1_15116339
63	S1_15641807
64	S1_16145881
65	S1_16261269
66	S1_16888381
67	S1_17821317
68	S1_17925565
69	S1_18033598
70	S1_18482217
71	S1_18737827
72	S1_19250860
73	S1_20035066
74	S1_20035067
75	S1_20071317
76	S1_20751138
77	S1_20916660
78	S1_23009774
79	S1_23063108
80	S1_23312406
81	S1_23457043
82	S1_24316770
83	S1_25820692
84	S1_26888664
85	S1_27649980
86	S1_28181343
87	S1_29072262
88	S1_29727269
89	S1_30379436
90	S1_31031498
91	S1_31031528
92	S1_31237764
93	S1_31837245
94	S1_31842946
95	S1_32953268
96	S1_32964683
97	S1_33628660
98	S1_35027364
99	S1_35097055
100	S1_35265994
101	S1_35582563
102	S1_36307173
103	S1_36887689
104	S1_37512598
105	S1_38114261
106	S1_38658261
107	S1_38764204
108	S1_39488786
109	S1_39747909
110	S1_40280985
111	S1_40741841
112	S1_41031824
113	S1_41137106
114	S1_41137948
115	S1_41137964
116	S1_41579436
117	S1_42297930
118	S1_42924247
119	S1_43084969
120	S1_43449513
121	S1_43908592
122	S1_44286768
123	S1_45996455
124	S1_46985492
125	S1_47696346
126	S1_47870968
127	S1_48229239
128	S1_48709169
129	S1_49500980
130	S1_51525547
131	S1_51871631
132	S1_52916905
133	S1_54503429
134	S1_56303442
135	S1_56303443
136	S1_56303505
137	S1_56304967
138	S1_56968000
139	S1_57301784
140	S1_58368575
141	S1_58368604
142	S1_59095085
143	S1_60425858
144	S1_61459934
145	S1_62094079
146	S1_62101310
147	S1_64234225
148	S1_64872342
149	S1_65829097
150	S1_66339020
151	S1_67803683
152	S1_69521353
153	S1_70716862
154	S1_71407741
155	S1_75491632
156	S1_77325713
157	S1_78186063
158	S1_78775880
159	S1_79804470
160	S1_80587467
161	S1_82571517
162	S1_83263467
163	S1_83263483
164	S1_83263487
165	S1_87698133
166	S1_88813862
167	S1_91298973
168	S1_92504213
169	S1_96189161
170	S1_97619618
171	S1_98120180
172	S1_98780153
173	S1_99960927
174	S1_101157765
175	S1_102134528
176	S1_103003311
177	S1_103501316
178	S1_106631081
179	S1_107494446
180	S1_109050899
181	S1_109720663
182	S1_110662138
183	S1_110719247
184	S1_111862847
185	S1_112753310
186	S1_116034262
187	S1_116034332
188	S1_116982331
189	S1_119583905
190	S1_120728260
191	S1_121006679
192	S1_121006680
193	S1_121006713
194	S1_121067683
195	S1_121067993
196	S1_121606123
197	S1_122313866
198	S1_124519005
199	S1_125663457
200	S1_126000700
201	S1_127024751
202	S1_127426465
203	S1_128287371
204	S1_128287409
205	S1_130545911
206	S1_132109795
207	S1_132879105
208	S1_133412644
209	S1_134959775
210	S1_137009850
211	S1_137659525
212	S1_138031616
213	S1_139580539
214	S1_140616567
215	S1_142585947
216	S1_143625254
217	S1_144658377
218	S1_147519798
219	S1_148311573
220	S1_148718676
221	S1_149356963
222	S1_149910878
223	S1_151829433
224	S1_152357296
225	S1_153134397
226	S1_153716188
227	S1_154541197
228	S1_156102053
229	S1_156548900
230	S1_156719227
231	S1_156727760
232	S1_157541325
233	S1_157543119
234	S1_157609546
235	S1_158333515
236	S1_158453069
237	S1_158699167
238	S1_158923421
239	S1_159134823
240	S1_159356085
241	S1_159360206
242	S1_159423133
243	S1_159493951
244	S1_159594541
245	S1_159839443
246	S1_160219850
247	S1_160637951
248	S1_160808747
249	S1_161154971
250	S1_161570542
251	S1_162107586
252	S1_162136142
253	S1_162836686
254	S1_163469274
255	S1_163469296
256	S1_163898062
257	S1_164692113
258	S1_165450174
259	S1_166023720
260	S1_166023747
261	S1_167135017
262	S1_167647527
263	S1_167949442

264	S1_168008843	317	S1_211300675	370	S1_242917637	423	S1_269182696	476	S2_15144076
265	S1_168689710	318	S1_212072799	371	S1_243760298	424	S1_269188555	477	S2_15344366
266	S1_168796839	319	S1_212933906	372	S1_244847822	425	S1_269220126	478	S2_16117552
267	S1_169419044	320	S1_214234600	373	S1_244847863	426	S1_269411921	479	S2_16123892
268	S1_169939249	321	S1_214813561	374	S1_244851476	427	S1_269722648	480	S2_17330870
269	S1_170019598	322	S1_215087957	375	S1_247524053	428	S1_269818666	481	S2_17385822
270	S1_170578782	323	S1_215662196	376	S1_248130970	429	S1_270243179	482	S2_17821684
271	S1_171358490	324	S1_217655489	377	S1_248284209	430	S1_270474230	483	S2_17821703
272	S1_171358627	325	S1_217715326	378	S1_248386156	431	S1_270882253	484	S2_18513157
273	S1_172138645	326	S1_218510409	379	S1_249530102	432	S1_270927677	485	S2_19085404
274	S1_173025041	327	S1_218983317	380	S1_250051388	433	S1_271492229	486	S2_19615774
275	S1_173696950	328	S1_218983905	381	S1_251536009	434	S1_271683580	487	S2_19655541
276	S1_174750940	329	S1_218990416	382	S1_252255907	435	S1_271687786	488	S2_21080373
277	S1_175333202	330	S1_219504206	383	S1_252256015	436	S1_271687795	489	S2_21954315
278	S1_175977293	331	S1_219553003	384	S1_252447173	437	S1_271687825	490	S2_22298018
279	S1_176223329	332	S1_219553007	385	S1_252447422	438	S1_271687826	491	S2_23477405
280	S1_177161768	333	S1_219553008	386	S1_252447497	439	S1_271826509	492	S2_23520524
281	S1_178004338	334	S1_219618009	387	S1_252447738	440	S1_272339587	493	S2_24292162
282	S1_178122122	335	S1_219618048	388	S1_252796259	441	S1_272575437	494	S2_26892547
283	S1_178495640	336	S1_219813423	389	S1_253403436	442	S1_272650516	495	S2_28301478
284	S1_179028582	337	S1_220075848	390	S1_254304670	443	S1_272664246	496	S2_28849485
285	S1_179386202	338	S1_220089478	391	S1_254478352	444	S1_272665785	497	S2_29446198
286	S1_180056328	339	S1_221633835	392	S1_254876118	445	S1_272665832	498	S2_29618099
287	S1_182245707	340	S1_221855498	393	S1_255175702	446	S1_272667425	499	S2_30053218
288	S1_183312379	341	S1_222322692	394	S1_255556484	447	S2_195576	500	S2_32019443
289	S1_185280049	342	S1_222332511	395	S1_256732795	448	S2_345089	501	S2_32972967
290	S1_187200569	343	S1_223086919	396	S1_257243412	449	S2_1373023	502	S2_34563377
291	S1_187973205	344	S1_223636984	397	S1_257546649	450	S2_2079676	503	S2_35103618
292	S1_188627207	345	S1_223645053	398	S1_257547637	451	S2_2559858	504	S2_35831117
293	S1_189493183	346	S1_223756504	399	S1_257686732	452	S2_3156871	505	S2_37698692
294	S1_190416342	347	S1_224811445	400	S1_257709832	453	S2_3156874	506	S2_39394484
295	S1_191103594	348	S1_225343636	401	S1_257767473	454	S2_3667791	507	S2_42014469
296	S1_192213592	349	S1_225495349	402	S1_257868135	455	S2_4285523	508	S2_42551200
297	S1_193262163	350	S1_226031711	403	S1_258172150	456	S2_4371872	509	S2_43074972
298	S1_193461346	351	S1_226991575	404	S1_258271226	457	S2_5135898	510	S2_44714524
299	S1_193819780	352	S1_227741519	405	S1_259286817	458	S2_5138486	511	S2_44746800
300	S1_194732636	353	S1_228944474	406	S1_259852303	459	S2_5151321	512	S2_45328713
301	S1_195266169	354	S1_228944904	407	S1_260471944	460	S2_5801777	513	S2_46403697
302	S1_197283478	355	S1_229651735	408	S1_262127007	461	S2_6562274	514	S2_46977637
303	S1_197992004	356	S1_230159701	409	S1_262630308	462	S2_8264822	515	S2_48612922
304	S1_198720990	357	S1_230661850	410	S1_262630837	463	S2_8838911	516	S2_49347453
305	S1_199915699	358	S1_230778503	411	S1_263493385	464	S2_9074653	517	S2_50742381
306	S1_200575324	359	S1_231289490	412	S1_263503567	465	S2_10273296	518	S2_51273528
307	S1_201160097	360	S1_231865698	413	S1_264168835	466	S2_10274094	519	S2_51520284
308	S1_201664629	361	S1_231865731	414	S1_264168846	467	S2_11037011	520	S2_51803087
309	S1_202897202	362	S1_233003988	415	S1_264873404	468	S2_11693099	521	S2_52375805
310	S1_205153331	363	S1_234832377	416	S1_265771958	469	S2_12134635	522	S2_52881161
311	S1_205783984	364	S1_236248143	417	S1_266933950	470	S2_12135142	523	S2_52986943
312	S1_206424544	365	S1_236812993	418	S1_267142751	471	S2_12762480	524	S2_53738000
313	S1_207984623	366	S1_239233989	419	S1_268006112	472	S2_12822767	525	S2_54604247
314	S1_208575904	367	S1_239238037	420	S1_268020752	473	S2_13411833	526	S2_55577779
315	S1_209438390	368	S1_241791382	421	S1_268535327	474	S2_14390534	527	S2_56301102
316	S1_210408671	369	S1_242399445	422	S1_269165289	475	S2_14746515	528	S2_57072711

529	S2_57622939	582	S2_103960400	635	S2_137044888	688	S2_152675830	741	S2_164784738
530	S2_58036012	583	S2_105806073	636	S2_137353153	689	S2_152802194	742	S2_165116280
531	S2_58267196	584	S2_107851740	637	S2_137523965	690	S2_153251004	743	S2_165414616
532	S2_59395284	585	S2_108088103	638	S2_137856788	691	S2_153285041	744	S2_165778091
533	S2_60417760	586	S2_109569537	639	S2_138523123	692	S2_153532280	745	S2_165919280
534	S2_60765335	587	S2_110553513	640	S2_138956247	693	S2_153802217	746	S2_166212400
535	S2_60777991	588	S2_111098040	641	S2_139107718	694	S2_153909489	747	S2_166378326
536	S2_60926020	589	S2_111745022	642	S2_139559993	695	S2_153909493	748	S2_166403558
537	S2_62882988	590	S2_112506847	643	S2_140090153	696	S2_154199398	749	S2_166511627
538	S2_63502337	591	S2_113042042	644	S2_140148416	697	S2_154479243	750	S2_166561007
539	S2_63642477	592	S2_113905677	645	S2_140958838	698	S2_155010886	751	S2_166607482
540	S2_63821312	593	S2_114511034	646	S2_141744731	699	S2_155351395	752	S2_166607508
541	S2_65569248	594	S2_115018229	647	S2_141907468	700	S2_155466279	753	S2_166607512
542	S2_66814935	595	S2_115194537	648	S2_141935315	701	S2_155610098	754	S2_166979210
543	S2_67757413	596	S2_116725640	649	S2_142460146	702	S2_155649148	755	S2_166979222
544	S2_68474897	597	S2_117243449	650	S2_143228424	703	S2_156110226	756	S2_167138001
545	S2_69678128	598	S2_117774259	651	S2_143317161	704	S2_156563281	757	S2_167455604
546	S2_70726612	599	S2_118644309	652	S2_143836249	705	S2_156612909	758	S2_167682445
547	S2_71501627	600	S2_119444467	653	S2_143883541	706	S2_156629032	759	S2_168213710
548	S2_72469829	601	S2_120523152	654	S2_144289790	707	S2_156951685	760	S2_168263784
549	S2_72778962	602	S2_120607326	655	S2_144388263	708	S2_157500874	761	S2_168544762
550	S2_73953316	603	S2_121116294	656	S2_144485520	709	S2_157654610	762	S2_168744335
551	S2_73953350	604	S2_121190182	657	S2_144966937	710	S2_158006059	763	S2_169057907
552	S2_74644693	605	S2_121195371	658	S2_145589537	711	S2_158216749	764	S2_169340707
553	S2_74644695	606	S2_122215880	659	S2_145737196	712	S2_158520007	765	S2_169664171
554	S2_76108469	607	S2_122325409	660	S2_145744597	713	S2_158833723	766	S2_169813732
555	S2_77056100	608	S2_122325412	661	S2_145863873	714	S2_158914352	767	S2_169869987
556	S2_78884285	609	S2_123258078	662	S2_146251297	715	S2_159041990	768	S2_169966102
557	S2_78894824	610	S2_123399937	663	S2_146272316	716	S2_159557150	769	S2_170071797
558	S2_78953505	611	S2_124539328	664	S2_146444763	717	S2_159947870	770	S2_170072386
559	S2_78953537	612	S2_124794888	665	S2_146863076	718	S2_160060188	771	S2_170072404
560	S2_82193126	613	S2_125176867	666	S2_147087403	719	S2_160060216	772	S2_170072415
561	S2_82899510	614	S2_127036648	667	S2_147214399	720	S2_160540698	773	S2_170264672
562	S2_85620679	615	S2_128209102	668	S2_147487098	721	S2_160564494	774	S2_170264675
563	S2_86199326	616	S2_128641070	669	S2_147687149	722	S2_160937451	775	S2_170377923
564	S2_89206072	617	S2_129017038	670	S2_147687681	723	S2_161017969	776	S2_170494116
565	S2_90832412	618	S2_129902820	671	S2_147758175	724	S2_161021094	777	S2_170861216
566	S2_92202100	619	S2_130205894	672	S2_148020176	725	S2_161099737	778	S2_170893123
567	S2_92892920	620	S2_130419718	673	S2_148684446	726	S2_161391153	779	S3_181246
568	S2_93168966	621	S2_130422280	674	S2_148834669	727	S2_161809192	780	S3_253470
569	S2_93409747	622	S2_131245465	675	S2_148922303	728	S2_162142414	781	S3_253511
570	S2_95561798	623	S2_131268806	676	S2_149014177	729	S2_162418732	782	S3_685027
571	S2_95672417	624	S2_131269452	677	S2_149228989	730	S2_162423861	783	S3_779792
572	S2_96232192	625	S2_131779956	678	S2_149917668	731	S2_162696474	784	S3_882414
573	S2_96847759	626	S2_132298457	679	S2_150046250	732	S2_162736153	785	S3_895472
574	S2_97847861	627	S2_132901816	680	S2_150749013	733	S2_163039319	786	S3_1085406
575	S2_101054001	628	S2_133863119	681	S2_150900919	734	S2_163316203	787	S3_1233486
576	S2_101896780	629	S2_134521029	682	S2_151536948	735	S2_163544203	788	S3_1261848
577	S2_102786026	630	S2_135203372	683	S2_152064602	736	S2_163556517	789	S3_1408351
578	S2_103329086	631	S2_135251651	684	S2_152108562	737	S2_163850961	790	S3_1408352
579	S2_103949868	632	S2_135747549	685	S2_152108615	738	S2_164121373	791	S3_1736898
580	S2_103953842	633	S2_136373443	686	S2_152654926	739	S2_164384991	792	S3_1840053
581	S2_103960398	634	S2_136920028	687	S2_152665303	740	S2_164437807	793	S3_2112043

794	S3_2272706	847	S3_14795646	900	S3_40062330	953	S3_88350342	1006	S3_143623891
795	S3_2284268	848	S3_15415411	901	S3_40062414	954	S3_90626409	1007	S3_145179840
796	S3_2285275	849	S3_15580035	902	S3_41303573	955	S3_93450697	1008	S3_145202570
797	S3_2435944	850	S3_16020094	903	S3_41838311	956	S3_95919817	1009	S3_145682793
798	S3_2995428	851	S3_17722351	904	S3_41838324	957	S3_96424864	1010	S3_146363044
799	S3_3008383	852	S3_18296517	905	S3_42782735	958	S3_96906582	1011	S3_148319783
800	S3_3180950	853	S3_18421240	906	S3_42967556	959	S3_96906670	1012	S3_150318458
801	S3_3533231	854	S3_18508100	907	S3_43871996	960	S3_97962806	1013	S3_153949409
802	S3_3554822	855	S3_19022621	908	S3_44281832	961	S3_97976531	1014	S3_154949045
803	S3_3856762	856	S3_19213723	909	S3_46163936	962	S3_99911924	1015	S3_156654549
804	S3_4063236	857	S3_19231902	910	S3_46164180	963	S3_101116342	1016	S3_157981680
805	S3_4223412	858	S3_19819257	911	S3_46924813	964	S3_101116391	1017	S3_163751831
806	S3_4868265	859	S3_19888893	912	S3_47793757	965	S3_103323378	1018	S3_164735489
807	S3_4868745	860	S3_20087906	913	S3_47793983	966	S3_106898541	1019	S3_166621921
808	S3_4992665	861	S3_20087931	914	S3_50467423	967	S3_106899475	1020	S3_166668219
809	S3_5134875	862	S3_20087934	915	S3_50995961	968	S3_107367979	1021	S3_166668223
810	S3_5134885	863	S3_20483493	916	S3_50995973	969	S3_107677965	1022	S3_167140464
811	S3_5134889	864	S3_21092479	917	S3_51517652	970	S3_109330186	1023	S3_168685595
812	S3_5605598	865	S3_21894985	918	S3_52022128	971	S3_110573455	1024	S3_169759070
813	S3_5607945	866	S3_21895067	919	S3_52604676	972	S3_112457315	1025	S3_170270316
814	S3_5861920	867	S3_22136610	920	S3_53951620	973	S3_115182805	1026	S3_171127281
815	S3_6170488	868	S3_22830909	921	S3_56182362	974	S3_116132336	1027	S3_173986191
816	S3_6176861	869	S3_23006848	922	S3_58285862	975	S3_116269267	1028	S3_174501798
817	S3_6184880	870	S3_23469227	923	S3_58847834	976	S3_117013629	1029	S3_175374155
818	S3_6187826	871	S3_23606909	924	S3_61148962	977	S3_117970304	1030	S3_176144664
819	S3_6242261	872	S3_23705792	925	S3_61149578	978	S3_119045091	1031	S3_176792751
820	S3_6454452	873	S3_23918680	926	S3_61576601	979	S3_119045223	1032	S3_177436516
821	S3_6691442	874	S3_24163492	927	S3_62688000	980	S3_120615715	1033	S3_177949479
822	S3_6766291	875	S3_24874530	928	S3_63136641	981	S3_120615869	1034	S3_179532716
823	S3_6798515	876	S3_24995891	929	S3_64626728	982	S3_121716261	1035	S3_180158687
824	S3_7299698	877	S3_24996490	930	S3_65276876	983	S3_122261805	1036	S3_181047024
825	S3_7504784	878	S3_26089465	931	S3_65734997	984	S3_122873658	1037	S3_181653208
826	S3_7804270	879	S3_27245035	932	S3_66899964	985	S3_123871582	1038	S3_182261964
827	S3_7891011	880	S3_28062265	933	S3_67021348	986	S3_124968415	1039	S3_183240861
828	S3_8283502	881	S3_28641583	934	S3_67530480	987	S3_125759406	1040	S3_183757962
829	S3_8633412	882	S3_28814551	935	S3_68471053	988	S3_126709279	1041	S3_185085798
830	S3_8800104	883	S3_29431251	936	S3_69502637	989	S3_128382276	1042	S3_186352356
831	S3_9252210	884	S3_30106424	937	S3_69976262	990	S3_129416102	1043	S3_186851368
832	S3_9388808	885	S3_30655790	938	S3_70078143	991	S3_130793121	1044	S3_187509859
833	S3_9466699	886	S3_30890879	939	S3_70295226	992	S3_132674050	1045	S3_187509869
834	S3_9466705	887	S3_31300639	940	S3_70295276	993	S3_133894601	1046	S3_187893913
835	S3_9703947	888	S3_31506982	941	S3_72043649	994	S3_134587124	1047	S3_187921821
836	S3_9837451	889	S3_31909388	942	S3_73742079	995	S3_135431128	1048	S3_188574932
837	S3_9974015	890	S3_32338653	943	S3_75089275	996	S3_136239486	1049	S3_188576777
838	S3_10498569	891	S3_32977376	944	S3_75888857	997	S3_138078715	1050	S3_189461725
839	S3_10880482	892	S3_33485279	945	S3_77710031	998	S3_138621998	1051	S3_190509993
840	S3_10893906	893	S3_33665302	946	S3_78673840	999	S3_139420179	1052	S3_192578506
841	S3_10976129	894	S3_34163334	947	S3_81312797	1000	S3_139938175	1053	S3_192581306
842	S3_11416627	895	S3_34605485	948	S3_82816994	1001	S3_140553369	1054	S3_192813633
843	S3_11961814	896	S3_35605722	949	S3_83540012	1002	S3_140941895	1055	S3_192813639
844	S3_12814095	897	S3_37246831	950	S3_86019876	1003	S3_140941919	1056	S3_193407954
845	S3_13345282	898	S3_37904729	951	S3_86761240	1004	S3_142059160	1057	S3_194170615
846	S3_14160923	899	S3_38784979	952	S3_86761543	1005	S3_142564171	1058	S3_196450309

1059	S3_196551154
1060	S3_196551164
1061	S3_196577152
1062	S3_196577158
1063	S3_197118488
1064	S3_197171575
1065	S3_197763841
1066	S3_198533289
1067	S3_199627466
1068	S3_199874023
1069	S3_199883267
1070	S3_200170541
1071	S3_200716439
1072	S3_201241235
1073	S3_201829085
1074	S3_202318106
1075	S3_202320713
1076	S3_202523319
1077	S3_203764676
1078	S3_203848706
1079	S3_204522738
1080	S3_204622620
1081	S3_205291114
1082	S3_205899058
1083	S3_206126014
1084	S3_206501581
1085	S3_206703676
1086	S3_207430343
1087	S3_208417634
1088	S3_209742233
1089	S3_210091024
1090	S3_210953784
1091	S3_211497614
1092	S3_211868879
1093	S3_212657847
1094	S3_212946181
1095	S3_213554104
1096	S3_213857743
1097	S3_214452791
1098	S3_214458346
1099	S3_214824552
1100	S3_215613212
1101	S3_216188692
1102	S3_216638301
1103	S3_217152315
1104	S3_217662174
1105	S3_217819786
1106	S3_218344080
1107	S3_218440816
1108	S3_218910983
1109	S3_218995039
1110	S3_219246900
1111	S3_219504567
1112	S3_219599584
1113	S3_220040222
1114	S3_220213649
1115	S3_220620277
1116	S3_220697389
1117	S3_220761155
1118	S3_221117350
1119	S3_221158835
1120	S3_221343381
1121	S3_221492679
1122	S3_221533300
1123	S3_221870681
1124	S3_221870920
1125	S3_221886871
1126	S3_222580308
1127	S3_222580309
1128	S3_222665678
1129	S3_222697733
1130	S3_222697753
1131	S3_223254348
1132	S3_223388448
1133	S3_224533781
1134	S3_224957006
1135	S3_225415233
1136	S3_225471879
1137	S3_226237418
1138	S3_226278875
1139	S3_226582881
1140	S3_226821964
1141	S3_226898122
1142	S3_227432740
1143	S3_227539131
1144	S3_228587039
1145	S3_228867924
1146	S3_228968797
1147	S3_229180498
1148	S3_229438185
1149	S3_229701989
1150	S3_230116211
1151	S3_230458147
1152	S3_230886457
1153	S3_231149163
1154	S3_231799445
1155	S3_232334895
1156	S3_232993524
1157	S3_233725050
1158	S3_234650968
1159	S3_234722727
1160	S3_235234401
1161	S3_235759223
1162	S3_235877704
1163	S3_236513323
1164	S3_236810502
1165	S3_236855978
1166	S3_237312917
1167	S3_237333808
1168	S3_237840706
1169	S3_237868197
1170	S3_238417107
1171	S3_238483803
1172	S3_238483806
1173	S3_238730522
1174	S3_239058390
1175	S3_239145023
1176	S3_239576414
1177	S3_239732912
1178	S3_240531628
1179	S3_240572490
1180	S3_241195588
1181	S3_241373244
1182	S3_241723981
1183	S3_242330803
1184	S3_242386553
1185	S3_242852517
1186	S3_243060855
1187	S3_244365056
1188	S3_244961118
1189	S3_244994329
1190	S3_245570318
1191	S3_246559127
1192	S3_247112921
1193	S3_247247734
1194	S3_247247761
1195	S3_247247788
1196	S3_247554015
1197	S3_247757144
1198	S3_247757173
1199	S3_248024018
1200	S3_248600851
1201	S3_248976087
1202	S3_248976090
1203	S3_248976097
1204	S3_249124554
1205	S3_249400650
1206	S3_249653742
1207	S3_249876702
1208	S3_250093073
1209	S3_250214478
1210	S3_250804723
1211	S3_250926630
1212	S3_250954307
1213	S3_250956039
1214	S3_251283071
1215	S3_251463653
1216	S3_251473588
1217	S3_251740352
1218	S3_252029953
1219	S3_252080858
1220	S3_252106914
1221	S3_252571379
1222	S3_252699918
1223	S3_253085322
1224	S3_253626943
1225	S3_253638948
1226	S3_253645785
1227	S3_253877279
1228	S3_254201098
1229	S3_254277203
1230	S3_254277206
1231	S3_254346936
1232	S3_254543440
1233	S3_254590991
1234	S3_254612541
1235	S3_254612570
1236	S3_254612572
1237	S3_255112839
1238	S3_255116246
1239	S3_255131991
1240	S3_255765293
1241	S3_255878563
1242	S3_255920003
1243	S3_256335786
1244	S3_256365665
1245	S3_256881022
1246	S3_256881635
1247	S3_257286942
1248	S3_257286944
1249	S3_257286945
1250	S3_257328667
1251	S3_257413682
1252	S3_257718902
1253	S4_40084
1254	S4_48216
1255	S4_236150
1256	S4_561146
1257	S4_595133
1258	S4_806383
1259	S4_1069608
1260	S4_1069611
1261	S4_1134559
1262	S4_1172827
1263	S4_1678877
1264	S4_1929498
1265	S4_2297460
1266	S4_2410298
1267	S4_3013732
1268	S4_3739982
1269	S4_3932764
1270	S4_3932765
1271	S4_4020850
1272	S4_4304120
1273	S4_4304229
1274	S4_4512367
1275	S4_5234339
1276	S4_6478602
1277	S4_8038002
1278	S4_8086617
1279	S4_8720894
1280	S4_8735579
1281	S4_9909790
1282	S4_10013430
1283	S4_10279943
1284	S4_10633911
1285	S4_10993283
1286	S4_11022903
1287	S4_11515639
1288	S4_11703769
1289	S4_11929432
1290	S4_12245592
1291	S4_12573153
1292	S4_12729066
1293	S4_13091776
1294	S4_13153775
1295	S4_13153797
1296	S4_13353659
1297	S4_13733321
1298	S4_13733335
1299	S4_13742975
1300	S4_14214804
1301	S4_14415822
1302	S4_14531156
1303	S4_14987979
1304	S4_15375816
1305	S4_15882207
1306	S4_16153860
1307	S4_16399626
1308	S4_17043072
1309	S4_17076327
1310	S4_17217623
1311	S4_17456025
1312	S4_17732203
1313	S4_18038321
1314	S4_18238469
1315	S4_18747577
1316	S4_20137301
1317	S4_21045444
1318	S4_21045480
1319	S4_21096616
1320	S4_21186559
1321	S4_21831127
1322	S4_22398741
1323	S4_23496642

1324	S4_24536152
1325	S4_25023690
1326	S4_26429583
1327	S4_27322663
1328	S4_27960373
1329	S4_30051651
1330	S4_30439652
1331	S4_31491410
1332	S4_32911457
1333	S4_34195386
1334	S4_35182288
1335	S4_40548547
1336	S4_40570344
1337	S4_40932331
1338	S4_41493300
1339	S4_43244715
1340	S4_44206932
1341	S4_45074851
1342	S4_45971308
1343	S4_46943727
1344	S4_47642028
1345	S4_48388347
1346	S4_49238488
1347	S4_50969907
1348	S4_52038355
1349	S4_52812632
1350	S4_53870285
1351	S4_55225019
1352	S4_56990692
1353	S4_58927723
1354	S4_60880036
1355	S4_61847872
1356	S4_63304164
1357	S4_64537517
1358	S4_65246321
1359	S4_67893542
1360	S4_68719137
1361	S4_70280399
1362	S4_72473058
1363	S4_75678381
1364	S4_76334753
1365	S4_77158684
1366	S4_77858121
1367	S4_79415754
1368	S4_80928953
1369	S4_83341235
1370	S4_84493253
1371	S4_85058181
1372	S4_85893597
1373	S4_88145778
1374	S4_92672655
1375	S4_93333693
1376	S4_95379836
1377	S4_96377793
1378	S4_97286404
1379	S4_97814559
1380	S4_97816332
1381	S4_99093339
1382	S4_100842847
1383	S4_104217535
1384	S4_104217536
1385	S4_104246738
1386	S4_104475908
1387	S4_104792763
1388	S4_107527791
1389	S4_109276290
1390	S4_110350406
1391	S4_111122366
1392	S4_112145158
1393	S4_113791530
1394	S4_114346234
1395	S4_116107412
1396	S4_117639539
1397	S4_117640186
1398	S4_121460143
1399	S4_123183146
1400	S4_124367652
1401	S4_124967320
1402	S4_124967332
1403	S4_128244170
1404	S4_130323571
1405	S4_132300655
1406	S4_135043773
1407	S4_137196704
1408	S4_137196713
1409	S4_137196752
1410	S4_137196765
1411	S4_137196775
1412	S4_137196823
1413	S4_137196835
1414	S4_137196844
1415	S4_137196849
1416	S4_137196858
1417	S4_137890641
1418	S4_144022885
1419	S4_144918969
1420	S4_145473655
1421	S4_146627519
1422	S4_147376471
1423	S4_153220579
1424	S4_154232130
1425	S4_159338579
1426	S4_162930457
1427	S4_162953230
1428	S4_165889264
1429	S4_166895295
1430	S4_167420595
1431	S4_168313733
1432	S4_170106836
1433	S4_170275175
1434	S4_170534705
1435	S4_170692179
1436	S4_172009980
1437	S4_172619316
1438	S4_173128854
1439	S4_173399142
1440	S4_173841357
1441	S4_173842812
1442	S4_173842897
1443	S4_173845605
1444	S4_173951595
1445	S4_173953023
1446	S4_176319872
1447	S4_178188832
1448	S4_178188885
1449	S4_178612966
1450	S4_179284604
1451	S4_179803866
1452	S4_180465800
1453	S4_180716608
1454	S4_181878451
1455	S4_183386661
1456	S4_186686209
1457	S4_187507485
1458	S4_187507531
1459	S4_187749886
1460	S4_188780350
1461	S4_189637957
1462	S4_190197820
1463	S4_190198026
1464	S4_191129933
1465	S4_192037803
1466	S4_192839554
1467	S4_193171909
1468	S4_193684446
1469	S4_194328600
1470	S4_194451957
1471	S4_195511524
1472	S4_195638202
1473	S4_195989144
1474	S4_196598571
1475	S4_196862073
1476	S4_197326248
1477	S4_197369887
1478	S4_197441369
1479	S4_198486945
1480	S4_198659668
1481	S4_199526158
1482	S4_201414410
1483	S4_201518766
1484	S4_201856023
1485	S4_202512264
1486	S4_202724437
1487	S4_203021292
1488	S4_203275189
1489	S4_203522642
1490	S4_204175636
1491	S4_205018264
1492	S4_205687456
1493	S4_206547860
1494	S4_206688914
1495	S4_207063280
1496	S4_208130239
1497	S4_208817618
1498	S4_209065056
1499	S4_209444309
1500	S4_210036992
1501	S4_210672456
1502	S4_210914991
1503	S4_211258926
1504	S4_211483880
1505	S4_211774427
1506	S4_212323230
1507	S4_212323893
1508	S4_212855437
1509	S4_212855445
1510	S4_213056243
1511	S4_213266693
1512	S4_213308992
1513	S4_213673264
1514	S4_214251303
1515	S4_214251384
1516	S4_214741864
1517	S4_214786585
1518	S4_214786599
1519	S4_214786611
1520	S4_215518594
1521	S4_215621393
1522	S4_215814389
1523	S4_215826684
1524	S4_215922485
1525	S4_216264002
1526	S4_216724975
1527	S4_216902515
1528	S4_217556647
1529	S4_217569546
1530	S4_218077811
1531	S4_218191150
1532	S4_218997001
1533	S4_219130590
1534	S4_219587261
1535	S4_219627943
1536	S4_220402156
1537	S4_220598225
1538	S4_220953358
1539	S4_220956393
1540	S4_221069972
1541	S4_221221118
1542	S4_221707508
1543	S4_221802510
1544	S4_222285121
1545	S4_222315477
1546	S5_50542
1547	S5_83922
1548	S5_431689
1549	S5_482262
1550	S5_608776
1551	S5_769009
1552	S5_769221
1553	S5_992960
1554	S5_1122819
1555	S5_1145132
1556	S5_1850090
1557	S5_1879743
1558	S5_1902471
1559	S5_1902559
1560	S5_1902560
1561	S5_2105063
1562	S5_2105086
1563	S5_2351550
1564	S5_2674576
1565	S5_2674621
1566	S5_2674622
1567	S5_2969922
1568	S5_2986983
1569	S5_3031821
1570	S5_3615744
1571	S5_3886612
1572	S5_4211087
1573	S5_4213205
1574	S5_4781348
1575	S5_5626037
1576	S5_5639739
1577	S5_6219021
1578	S5_6535912
1579	S5_7430529
1580	S5_7445992
1581	S5_7976369
1582	S5_8264487
1583	S5_8635428
1584	S5_8697950
1585	S5_8823103
1586	S5_9316109
1587	S5_9346379
1588	S5_9866294

1589	S5_10189277
1590	S5_10264906
1591	S5_10264912
1592	S5_10264918
1593	S5_10268747
1594	S5_10294829
1595	S5_10404189
1596	S5_10568044
1597	S5_10873205
1598	S5_11345386
1599	S5_11401086
1600	S5_12039305
1601	S5_12646412
1602	S5_12736568
1603	S5_12761161
1604	S5_14425241
1605	S5_14425279
1606	S5_14947693
1607	S5_15023555
1608	S5_15575306
1609	S5_16109491
1610	S5_16245350
1611	S5_16293105
1612	S5_16449582
1613	S5_16987647
1614	S5_18013813
1615	S5_19256770
1616	S5_19628862
1617	S5_20000149
1618	S5_20021770
1619	S5_20987495
1620	S5_22597333
1621	S5_22597389
1622	S5_23169374
1623	S5_24217762
1624	S5_24781155
1625	S5_26445470
1626	S5_27068301
1627	S5_27068314
1628	S5_27093992
1629	S5_27161227
1630	S5_27623318
1631	S5_27623322
1632	S5_27695629
1633	S5_28040327
1634	S5_29662949
1635	S5_30195998
1636	S5_30787082
1637	S5_32441228
1638	S5_33395831
1639	S5_33958933
1640	S5_35833239
1641	S5_35933793
1642	S5_36762256
1643	S5_36879676
1644	S5_37317419
1645	S5_37604656
1646	S5_38618956
1647	S5_39871223
1648	S5_40758415
1649	S5_43216434
1650	S5_43869751
1651	S5_44549437
1652	S5_45166646
1653	S5_45166674
1654	S5_45166843
1655	S5_49314686
1656	S5_50602973
1657	S5_51126448
1658	S5_52988522
1659	S5_55150930
1660	S5_55150992
1661	S5_55151265
1662	S5_56507201
1663	S5_58003499
1664	S5_59403389
1665	S5_59404565
1666	S5_59991012
1667	S5_62989377
1668	S5_64167904
1669	S5_65143612
1670	S5_65610463
1671	S5_66139112
1672	S5_66522423
1673	S5_67569699
1674	S5_70801772
1675	S5_71385870
1676	S5_73574446
1677	S5_75392509
1678	S5_76292718
1679	S5_80537412
1680	S5_82249820
1681	S5_86660470
1682	S5_87972278
1683	S5_88558143
1684	S5_93417011
1685	S5_93597517
1686	S5_94039299
1687	S5_95652386
1688	S5_97489439
1689	S5_98824486
1690	S5_99860723
1691	S5_101209975
1692	S5_102010651
1693	S5_104758513
1694	S5_107665441
1695	S5_108526970
1696	S5_109494781
1697	S5_110187986
1698	S5_111541672
1699	S5_113591831
1700	S5_114712581
1701	S5_115426989
1702	S5_118574634
1703	S5_120859965
1704	S5_121536575
1705	S5_123762990
1706	S5_124737570
1707	S5_126639339
1708	S5_127305377
1709	S5_131288209
1710	S5_131956214
1711	S5_131956331
1712	S5_132723874
1713	S5_133754625
1714	S5_134800121
1715	S5_135465507
1716	S5_136907208
1717	S5_137541372
1718	S5_139212881
1719	S5_140180440
1720	S5_140779561
1721	S5_141420552
1722	S5_141438504
1723	S5_142422691
1724	S5_143997083
1725	S5_144000944
1726	S5_144772588
1727	S5_145581432
1728	S5_146089067
1729	S5_147243956
1730	S5_148978516
1731	S5_149312570
1732	S5_151229127
1733	S5_151229154
1734	S5_155098307
1735	S5_157850956
1736	S5_159479373
1737	S5_160079410
1738	S5_161056592
1739	S5_163445414
1740	S5_166894147
1741	S5_167831137
1742	S5_169099805
1743	S5_170665017
1744	S5_173087338
1745	S5_173087538
1746	S5_174551313
1747	S5_176821306
1748	S5_177949380
1749	S5_177960311
1750	S5_177963537
1751	S5_178566567
1752	S5_180614512
1753	S5_181627396
1754	S5_182661638
1755	S5_183280310
1756	S5_185478792
1757	S5_186085945
1758	S5_186786121
1759	S5_186786122
1760	S5_187470397
1761	S5_188358427
1762	S5_188865759
1763	S5_190829139
1764	S5_191484028
1765	S5_192062598
1766	S5_193497873
1767	S5_195195696
1768	S5_196458409
1769	S5_197051491
1770	S5_197654069
1771	S5_198166036
1772	S5_198763487
1773	S5_198768007
1774	S5_199275772
1775	S5_200282666
1776	S5_202882438
1777	S5_203710038
1778	S5_204260272
1779	S5_205889382
1780	S5_206433194
1781	S5_208791242
1782	S5_209524493
1783	S5_210132976
1784	S5_210138982
1785	S5_211360129
1786	S5_211937547
1787	S5_212620255
1788	S5_213147224
1789	S5_213803537
1790	S5_213908541
1791	S5_213985177
1792	S5_214049068
1793	S5_214529650
1794	S5_215671490
1795	S5_215972421
1796	S5_216808528
1797	S5_216808588
1798	S5_217432338
1799	S5_217504323
1800	S5_218009705
1801	S5_218702656
1802	S5_219854887
1803	S5_220983156
1804	S5_220983158
1805	S5_220983219
1806	S5_221450387
1807	S5_221470088
1808	S5_221597228
1809	S5_221937301
1810	S5_222898091
1811	S5_223177817
1812	S5_223346293
1813	S5_223419723
1814	S5_223950207
1815	S5_224307549
1816	S5_224589685
1817	S5_225158364
1818	S5_225809150
1819	S5_225809420
1820	S5_226519432
1821	S5_226522552
1822	S5_226622808
1823	S5_226623033
1824	S5_226623146
1825	S5_227128999
1826	S5_227168425
1827	S5_227259982
1828	S5_227260043
1829	S5_227782410
1830	S5_227783289
1831	S5_227837981
1832	S5_227844713
1833	S5_227852478
1834	S5_227887193
1835	S5_228229074
1836	S5_228306428
1837	S5_228306585
1838	S5_228472435
1839	S5_228895449
1840	S5_229386213
1841	S5_229532342
1842	S5_229550743
1843	S5_229887616
1844	S5_229928831
1845	S5_229928869
1846	S5_230124872
1847	S5_230256375
1848	S5_230404119
1849	S5_230404137
1850	S5_230956228
1851	S5_231602951
1852	S5_231641146
1853	S5_232035428

1854	S5_232039113
1855	S5_232199425
1856	S5_232293477
1857	S5_232736088
1858	S5_232811709
1859	S5_232815642
1860	S5_232848007
1861	S5_232849002
1862	S5_232878003
1863	S5_232880473
1864	S5_232880532
1865	S5_233299100
1866	S6_63856
1867	S6_185156
1868	S6_185230
1869	S6_185234
1870	S6_203650
1871	S6_644196
1872	S6_799033
1873	S6_1475586
1874	S6_1807402
1875	S6_1905320
1876	S6_1906922
1877	S6_2342999
1878	S6_2491765
1879	S6_2535039
1880	S6_2535507
1881	S6_2800508
1882	S6_2878699
1883	S6_3289792
1884	S6_3289824
1885	S6_3313569
1886	S6_3354268
1887	S6_3380508
1888	S6_3416968
1889	S6_3426248
1890	S6_3426249
1891	S6_3880147
1892	S6_3979733
1893	S6_4533635
1894	S6_4533659
1895	S6_4548746
1896	S6_5075711
1897	S6_5075720
1898	S6_5475381
1899	S6_6241256
1900	S6_7032986
1901	S6_7132169
1902	S6_7204160
1903	S6_7460032
1904	S6_7559936
1905	S6_8433545
1906	S6_8523329
1907	S6_10402968
1908	S6_10577195
1909	S6_10663125
1910	S6_10930996
1911	S6_10946326
1912	S6_11594667
1913	S6_12303456
1914	S6_12621277
1915	S6_13242115
1916	S6_13573937
1917	S6_14088435
1918	S6_14939343
1919	S6_14939505
1920	S6_16502579
1921	S6_17049665
1922	S6_19003995
1923	S6_19004002
1924	S6_19291180
1925	S6_19709550
1926	S6_20292477
1927	S6_21087750
1928	S6_21129881
1929	S6_21315629
1930	S6_21868008
1931	S6_23241599
1932	S6_24071372
1933	S6_24703685
1934	S6_25251242
1935	S6_25368819
1936	S6_26635072
1937	S6_27960985
1938	S6_27960999
1939	S6_28563259
1940	S6_29658223
1941	S6_30329025
1942	S6_32518484
1943	S6_33665369
1944	S6_34513038
1945	S6_35046140
1946	S6_35648953
1947	S6_37184180
1948	S6_40151799
1949	S6_40917232
1950	S6_41881053
1951	S6_42758645
1952	S6_44720832
1953	S6_45713198
1954	S6_48549947
1955	S6_50148570
1956	S6_53730402
1957	S6_54919572
1958	S6_56684249
1959	S6_59758681
1960	S6_60344268
1961	S6_61248802
1962	S6_61756160
1963	S6_61756162
1964	S6_62389545
1965	S6_62389576
1966	S6_62976472
1967	S6_62976483
1968	S6_64238053
1969	S6_65066921
1970	S6_66273967
1971	S6_67328959
1972	S6_69691826
1973	S6_70900971
1974	S6_71482414
1975	S6_72854102
1976	S6_74204813
1977	S6_75059825
1978	S6_80159266
1979	S6_80839906
1980	S6_81454765
1981	S6_82515896
1982	S6_83260928
1983	S6_85221489
1984	S6_86856908
1985	S6_87754124
1986	S6_88545887
1987	S6_89460088
1988	S6_91055678
1989	S6_92540027
1990	S6_94529708
1991	S6_96968375
1992	S6_97717873
1993	S6_99008755
1994	S6_99508770
1995	S6_101455378
1996	S6_102220152
1997	S6_103971762
1998	S6_104837490
1999	S6_105599306
2000	S6_106281807
2001	S6_107737939
2002	S6_109320944
2003	S6_109468134
2004	S6_109468152
2005	S6_110113681
2006	S6_110832836
2007	S6_112572947
2008	S6_114355046
2009	S6_115314384
2010	S6_115314436
2011	S6_116303999
2012	S6_117502594
2013	S6_121065364
2014	S6_122735093
2015	S6_127869076
2016	S6_128522241
2017	S6_129359684
2018	S6_130113343
2019	S6_131919598
2020	S6_132536030
2021	S6_133268808
2022	S6_133512033
2023	S6_134277552
2024	S6_138630268
2025	S6_139422632
2026	S6_140445246
2027	S6_141530592
2028	S6_141785063
2029	S6_145076589
2030	S6_146214612
2031	S6_147522904
2032	S6_149194856
2033	S6_149843475
2034	S6_150364002
2035	S6_151414316
2036	S6_152694212
2037	S6_153577470
2038	S6_153577553
2039	S6_155455315
2040	S6_156920865
2041	S6_160376465
2042	S6_160937796
2043	S6_161439427
2044	S6_162239688
2045	S6_162941128
2046	S6_163504805
2047	S6_164672613
2048	S6_166179657
2049	S6_167311440
2050	S6_168108457
2051	S6_169105950
2052	S6_169107346
2053	S6_169947968
2054	S6_170478706
2055	S6_171201726
2056	S6_171251290
2057	S6_171575285
2058	S6_172698632
2059	S6_173970285
2060	S6_173970326
2061	S6_174639164
2062	S6_175594048
2063	S6_175746115
2064	S6_175879518
2065	S6_176288645
2066	S6_176821715
2067	S6_177023248
2068	S6_177790639
2069	S6_178493073
2070	S6_179285880
2071	S6_179570639
2072	S6_180436241
2073	S6_181438764
2074	S6_181726026
2075	S6_182781789
2076	S6_183320008
2077	S6_183770875
2078	S6_184522306
2079	S6_185462037
2080	S6_186130555
2081	S6_187074175
2082	S6_188128187
2083	S6_188690054
2084	S6_189702364
2085	S6_190313632
2086	S6_190851965
2087	S6_190917401
2088	S6_192004201
2089	S6_192633327
2090	S6_192687933
2091	S6_193188020
2092	S6_194236945
2093	S6_195393393
2094	S6_195531843
2095	S6_196109934
2096	S6_196770872
2097	S6_197462868
2098	S6_198589345
2099	S6_199245186
2100	S6_200038944
2101	S6_200215819
2102	S6_200755473
2103	S6_200903970
2104	S6_201367340
2105	S6_201549584
2106	S6_202147420
2107	S6_202356072
2108	S6_202768198
2109	S6_202768252
2110	S6_202800609
2111	S6_203176123
2112	S6_203713019
2113	S6_204116005
2114	S6_204520001
2115	S6_204520133
2116	S6_204520213
2117	S6_204557903
2118	S6_204652957

2119	S6_205016941	2172	S6_220424726	2225	S6_235352969	2278	S7_14015307	2331	S7_56142161
2120	S6_205018983	2173	S6_220429709	2226	S6_235664509	2279	S7_14015310	2332	S7_57257540
2121	S6_205018984	2174	S6_220903727	2227	S6_235676366	2280	S7_14015313	2333	S7_58078643
2122	S6_205095935	2175	S6_221646100	2228	S6_235676367	2281	S7_14842698	2334	S7_58195201
2123	S6_205140372	2176	S6_222309508	2229	S6_235786682	2282	S7_15456592	2335	S7_58752842
2124	S6_206001928	2177	S6_223042741	2230	S6_235889349	2283	S7_15774276	2336	S7_59300575
2125	S6_206840927	2178	S6_223658257	2231	S6_235912193	2284	S7_17225974	2337	S7_60804768
2126	S6_207390243	2179	S6_224085629	2232	S6_235912244	2285	S7_17226021	2338	S7_62163472
2127	S6_207924007	2180	S6_224161430	2233	S6_235912280	2286	S7_18508851	2339	S7_63383088
2128	S6_208448605	2181	S6_224373924	2234	S6_236435830	2287	S7_18509905	2340	S7_66177242
2129	S6_208471687	2182	S6_224376158	2235	S7_272540	2288	S7_18509912	2341	S7_66997070
2130	S6_208862137	2183	S6_224376804	2236	S7_469062	2289	S7_19678759	2342	S7_67016430
2131	S6_209007359	2184	S6_224531168	2237	S7_1075453	2290	S7_20350192	2343	S7_67018551
2132	S6_209438224	2185	S6_224714546	2238	S7_1535593	2291	S7_20398019	2344	S7_68614483
2133	S6_209570952	2186	S6_225215759	2239	S7_1776525	2292	S7_20398020	2345	S7_69365654
2134	S6_210053919	2187	S6_225735415	2240	S7_1838908	2293	S7_20784617	2346	S7_70032560
2135	S6_210072964	2188	S6_226276630	2241	S7_2577265	2294	S7_21295251	2347	S7_71746084
2136	S6_210239798	2189	S6_226508975	2242	S7_3025388	2295	S7_22066880	2348	S7_73665279
2137	S6_210245613	2190	S6_226517245	2243	S7_3025389	2296	S7_22822241	2349	S7_74559292
2138	S6_210997991	2191	S6_226886912	2244	S7_3106601	2297	S7_24029567	2350	S7_76858886
2139	S6_210998001	2192	S6_227058392	2245	S7_3164406	2298	S7_24469863	2351	S7_77364226
2140	S6_210998030	2193	S6_227058581	2246	S7_3325949	2299	S7_24470418	2352	S7_77364253
2141	S6_211130922	2194	S6_227195491	2247	S7_3595394	2300	S7_27842630	2353	S7_78511286
2142	S6_211838069	2195	S6_227562172	2248	S7_3669702	2301	S7_28959708	2354	S7_80145727
2143	S6_212068739	2196	S6_227929345	2249	S7_4241665	2302	S7_28971226	2355	S7_81876665
2144	S6_212203814	2197	S6_228435392	2250	S7_4241666	2303	S7_29830548	2356	S7_82262001
2145	S6_212531442	2198	S6_228747916	2251	S7_4545845	2304	S7_30534772	2357	S7_83526784
2146	S6_212531477	2199	S6_228752332	2252	S7_4755710	2305	S7_31847899	2358	S7_84186550
2147	S6_212725958	2200	S6_228962348	2253	S7_4763390	2306	S7_32689436	2359	S7_85193883
2148	S6_212940570	2201	S6_229092668	2254	S7_5687113	2307	S7_32691335	2360	S7_86261446
2149	S6_212966078	2202	S6_229415706	2255	S7_5880738	2308	S7_36624132	2361	S7_87267677
2150	S6_213526152	2203	S6_229609447	2256	S7_7598686	2309	S7_36879343	2362	S7_88825800
2151	S6_214408303	2204	S6_229746780	2257	S7_7598687	2310	S7_37520309	2363	S7_90272340
2152	S6_214974478	2205	S6_229946852	2258	S7_7673058	2311	S7_38229585	2364	S7_95818810
2153	S6_215956308	2206	S6_230400660	2259	S7_7837531	2312	S7_39824789	2365	S7_95938753
2154	S6_216623589	2207	S6_230783709	2260	S7_7837532	2313	S7_40360764	2366	S7_98753854
2155	S6_216653729	2208	S6_231324333	2261	S7_8194799	2314	S7_44584812	2367	S7_99557856
2156	S6_216759457	2209	S6_231387995	2262	S7_8264288	2315	S7_46102747	2368	S7_100444201
2157	S6_216858487	2210	S6_231992591	2263	S7_8527376	2316	S7_46102898	2369	S7_102716372
2158	S6_217105748	2211	S6_232445951	2264	S7_8534204	2317	S7_46962901	2370	S7_103722988
2159	S6_217274488	2212	S6_232668942	2265	S7_8534263	2318	S7_47165828	2371	S7_103723673
2160	S6_217673716	2213	S6_233197162	2266	S7_8891338	2319	S7_47728861	2372	S7_104883674
2161	S6_217826544	2214	S6_233529058	2267	S7_9035006	2320	S7_48487277	2373	S7_105689388
2162	S6_218076018	2215	S6_233682441	2268	S7_9545837	2321	S7_49221875	2374	S7_106469391
2163	S6_218438685	2216	S6_233706231	2269	S7_10100177	2322	S7_51064920	2375	S7_109562934
2164	S6_218918616	2217	S6_234211570	2270	S7_10159685	2323	S7_51908552	2376	S7_110687636
2165	S6_218953481	2218	S6_234211574	2271	S7_10665032	2324	S7_51908578	2377	S7_112133996
2166	S6_219189814	2219	S6_234223526	2272	S7_10667766	2325	S7_52746736	2378	S7_113937551
2167	S6_219473031	2220	S6_234650760	2273	S7_10824450	2326	S7_52760580	2379	S7_114111536
2168	S6_219519343	2221	S6_234808366	2274	S7_11388495	2327	S7_53905270	2380	S7_115437781
2169	S6_219735368	2222	S6_235148416	2275	S7_11457438	2328	S7_54001415	2381	S7_116656792
2170	S6_220380976	2223	S6_235163363	2276	S7_12170811	2329	S7_54001776	2382	S7_116657572
2171	S6_220424614	2224	S6_235163379	2277	S7_12327149	2330	S7_55102582	2383	S7_116657573

2384	S7_117848870
2385	S7_118554947
2386	S7_118555019
2387	S7_119466056
2388	S7_119833980
2389	S7_120268627
2390	S7_121396686
2391	S7_121723605
2392	S7_122343695
2393	S7_122721912
2394	S7_122721955
2395	S7_123004888
2396	S7_124545116
2397	S7_125445657
2398	S7_127022145
2399	S7_127523626
2400	S7_129800595
2401	S7_130539918
2402	S7_131116796
2403	S7_134345300
2404	S7_138664381
2405	S7_140310912
2406	S7_140887797
2407	S7_144906089
2408	S7_146441692
2409	S7_147345214
2410	S7_147345259
2411	S7_151202454
2412	S7_151220113
2413	S7_152110977
2414	S7_152111049
2415	S7_152273546
2416	S7_153818070
2417	S7_153818186
2418	S7_154545767
2419	S7_156382041
2420	S7_156388601
2421	S7_158463477
2422	S7_159221123
2423	S7_160390070
2424	S7_160396609
2425	S7_160396629
2426	S7_160396648
2427	S7_160396649
2428	S7_160396652
2429	S7_163800910
2430	S7_168595152
2431	S7_169544415
2432	S7_171302830
2433	S7_173710732
2434	S7_174820906
2435	S7_174930814
2436	S7_175407707
2437	S7_176729934
2438	S7_176920288
2439	S7_177593353
2440	S7_178247810
2441	S7_178923291
2442	S7_179151253
2443	S7_179180664
2444	S7_179527650
2445	S7_179979827
2446	S7_180286213
2447	S7_181002348
2448	S7_181857905
2449	S7_182420540
2450	S7_183428345
2451	S7_184814542
2452	S7_186563471
2453	S7_187398019
2454	S7_187973901
2455	S7_188563369
2456	S7_189277314
2457	S7_190883559
2458	S7_192375445
2459	S7_193028066
2460	S7_193587045
2461	S7_195334144
2462	S7_195568420
2463	S7_196094370
2464	S7_196732067
2465	S7_197365934
2466	S7_197839274
2467	S7_198460667
2468	S7_198556297
2469	S7_198682006
2470	S7_199162463
2471	S7_199800706
2472	S7_199800707
2473	S7_199800709
2474	S7_200318429
2475	S7_201659257
2476	S7_202249709
2477	S7_202774677
2478	S7_203020306
2479	S7_203774589
2480	S7_204411647
2481	S7_204609502
2482	S7_205378062
2483	S7_205679039
2484	S7_205927165
2485	S7_205949107
2486	S7_206593543
2487	S7_206593551
2488	S7_206593556
2489	S7_206593557
2490	S7_206593566
2491	S7_207070984
2492	S7_207279404
2493	S7_207663684
2494	S7_208795952
2495	S7_209010488
2496	S7_209021095
2497	S7_209681627
2498	S7_211625816
2499	S7_211682712
2500	S7_212101950
2501	S7_212488474
2502	S7_212718404
2503	S7_212725547
2504	S7_213259174
2505	S7_214094194
2506	S7_214154216
2507	S7_214197365
2508	S7_215565316
2509	S7_216081850
2510	S7_216656286
2511	S7_216783065
2512	S7_216783809
2513	S7_216783844
2514	S7_216783852
2515	S7_216783853
2516	S7_217193012
2517	S7_217785425
2518	S7_217973503
2519	S7_218021263
2520	S7_218374132
2521	S7_218735461
2522	S7_218946200
2523	S7_219728248
2524	S7_219728272
2525	S7_220392209
2526	S7_221427365
2527	S7_221493753
2528	S7_221718841
2529	S7_222303778
2530	S7_223200365
2531	S7_223207483
2532	S7_223247216
2533	S7_223247217
2534	S7_223829532
2535	S7_224337343
2536	S7_224377006
2537	S7_224926645
2538	S7_225446145
2539	S7_225446170
2540	S7_226088853
2541	S7_226206752
2542	S7_226729662
2543	S7_226819308
2544	S7_227310714
2545	S7_227589816
2546	S7_227766374
2547	S7_227868802
2548	S7_228390474
2549	S7_228436078
2550	S7_228611779
2551	S7_228917059
2552	S7_228953074
2553	S7_229192366
2554	S7_229195180
2555	S7_229289540
2556	S7_229815398
2557	S7_230276036
2558	S7_230373435
2559	S7_230963487
2560	S7_231311196
2561	S7_231526154
2562	S8_88680
2563	S8_377467
2564	S8_764933
2565	S8_783308
2566	S8_1307939
2567	S8_1376099
2568	S8_1493950
2569	S8_1611929
2570	S8_2025005
2571	S8_2066400
2572	S8_2186834
2573	S8_2660631
2574	S8_3346072
2575	S8_3410140
2576	S8_3514467
2577	S8_4023853
2578	S8_4643335
2579	S8_10377492
2580	S8_11810314
2581	S8_14451175
2582	S8_15267329
2583	S8_15532523
2584	S8_16963915
2585	S8_18941240
2586	S8_19563655
2587	S8_20806463
2588	S8_21642531
2589	S8_23067418
2590	S8_23919525
2591	S8_25263234
2592	S8_26214420
2593	S8_28917832
2594	S8_29618158
2595	S8_29863924
2596	S8_29863944
2597	S8_30387348
2598	S8_31415605
2599	S8_31982336
2600	S8_34142945
2601	S8_36053452
2602	S8_36778227
2603	S8_37569134
2604	S8_39506699
2605	S8_40165422
2606	S8_42948067
2607	S8_43891744
2608	S8_45563065
2609	S8_50185123
2610	S8_50495840
2611	S8_51590150
2612	S8_52096711
2613	S8_52496363
2614	S8_53743429
2615	S8_55046282
2616	S8_55610951
2617	S8_56210030
2618	S8_57345956
2619	S8_59808319
2620	S8_61587413
2621	S8_62314127
2622	S8_62986510
2623	S8_63810167
2624	S8_64921915
2625	S8_65908131
2626	S8_66486058
2627	S8_67980096
2628	S8_67994422
2629	S8_68791821
2630	S8_69807046
2631	S8_73343452
2632	S8_74403280
2633	S8_75305235
2634	S8_76258622
2635	S8_77716707
2636	S8_78338167
2637	S8_78338227
2638	S8_79331590
2639	S8_79543655
2640	S8_79934352
2641	S8_80102426
2642	S8_80698388
2643	S8_81585433
2644	S8_83124737
2645	S8_84727889
2646	S8_85702540
2647	S8_86420343
2648	S8_87523944

2649	S8_88122613
2650	S8_88910303
2651	S8_89679367
2652	S8_90044786
2653	S8_90820572
2654	S8_91916783
2655	S8_93625635
2656	S8_93625830
2657	S8_95483759
2658	S8_96759425
2659	S8_100386700
2660	S8_101883186
2661	S8_108169141
2662	S8_108849267
2663	S8_108849274
2664	S8_111605000
2665	S8_112468911
2666	S8_112970999
2667	S8_113846481
2668	S8_114146140
2669	S8_117672592
2670	S8_117672629
2671	S8_118176102
2672	S8_119065551
2673	S8_119917054
2674	S8_121769645
2675	S8_121949774
2676	S8_122204243
2677	S8_122536335
2678	S8_122705285
2679	S8_122785995
2680	S8_122817249
2681	S8_122912159
2682	S8_123415677
2683	S8_123747513
2684	S8_123747695
2685	S8_123881421
2686	S8_123881609
2687	S8_124036172
2688	S8_124308505
2689	S8_124400493
2690	S8_124644957
2691	S8_124759152
2692	S8_124915701
2693	S8_124915718
2694	S8_124915733
2695	S8_124917848
2696	S8_124918019
2697	S8_125456241
2698	S8_125542942
2699	S8_126004422
2700	S8_126075416
2701	S8_126519999
2702	S8_127029940
2703	S8_127101344
2704	S8_127194262
2705	S8_127234144
2706	S8_127234201
2707	S8_127242809
2708	S8_127242926
2709	S8_127630281
2710	S8_127683996
2711	S8_128125021
2712	S8_128132657
2713	S8_128507690
2714	S8_128625719
2715	S8_129446617
2716	S8_129652880
2717	S8_129781105
2718	S8_130368566
2719	S8_131205657
2720	S8_131559921
2721	S8_131607794
2722	S8_131890047
2723	S8_132027851
2724	S8_132232796
2725	S8_132558780
2726	S8_132935172
2727	S8_133033327
2728	S8_133314848
2729	S8_133808949
2730	S8_134012230
2731	S8_134152165
2732	S8_134336067
2733	S8_135327269
2734	S8_135391290
2735	S8_135480065
2736	S8_135500042
2737	S8_135773828
2738	S8_135891997
2739	S8_135930348
2740	S8_136000534
2741	S8_136418391
2742	S8_136521115
2743	S8_136814049
2744	S8_136932188
2745	S8_137070242
2746	S8_137386137
2747	S8_137596010
2748	S8_137630696
2749	S8_137630707
2750	S8_138054686
2751	S8_138144629
2752	S8_138360266
2753	S8_138859540
2754	S8_139214962
2755	S8_139214993
2756	S8_139347049
2757	S8_139507918
2758	S8_139633987
2759	S8_139733747
2760	S8_139989803
2761	S8_140480418
2762	S8_140501890
2763	S8_140646109
2764	S8_140649942
2765	S8_140650713
2766	S8_141127095
2767	S8_141273679
2768	S8_141608964
2769	S8_141813555
2770	S8_142170822
2771	S8_142333145
2772	S8_142698523
2773	S8_142734164
2774	S8_142884487
2775	S8_142959972
2776	S8_143075900
2777	S8_143087084
2778	S8_143561767
2779	S8_143633310
2780	S8_143633313
2781	S8_143803108
2782	S8_144177913
2783	S8_144234427
2784	S8_144234437
2785	S8_144489971
2786	S8_144489975
2787	S8_144489976
2788	S8_144489994
2789	S8_144490952
2790	S8_144490958
2791	S8_144785212
2792	S8_145038235
2793	S8_145038246
2794	S9_1145
2795	S9_1154
2796	S9_1178
2797	S9_682780
2798	S9_797286
2799	S9_1771455
2800	S9_1841697
2801	S9_2312895
2802	S9_2460459
2803	S9_2506229
2804	S9_2973691
2805	S9_3012230
2806	S9_3450774
2807	S9_3799368
2808	S9_4355459
2809	S9_4435976
2810	S9_4466107
2811	S9_4517642
2812	S9_4600228
2813	S9_4679970
2814	S9_4679976
2815	S9_5310836
2816	S9_5322983
2817	S9_5880547
2818	S9_6127622
2819	S9_6583515
2820	S9_7096849
2821	S9_7096860
2822	S9_7632769
2823	S9_8268141
2824	S9_8700534
2825	S9_8974203
2826	S9_9474969
2827	S9_10023524
2828	S9_10571662
2829	S9_10669708
2830	S9_11535589
2831	S9_12707262
2832	S9_13649277
2833	S9_15240804
2834	S9_16081897
2835	S9_16323341
2836	S9_16896752
2837	S9_18637661
2838	S9_19706537
2839	S9_19726089
2840	S9_20988975
2841	S9_21715226
2842	S9_22441207
2843	S9_22962335
2844	S9_23721551
2845	S9_23784851
2846	S9_24943074
2847	S9_25629279
2848	S9_28125967
2849	S9_30137802
2850	S9_30648356
2851	S9_31256608
2852	S9_32458046
2853	S9_33644646
2854	S9_34265097
2855	S9_34806701
2856	S9_35325230
2857	S9_36527039
2858	S9_37190429
2859	S9_37192984
2860	S9_37830967
2861	S9_38747009
2862	S9_39467855
2863	S9_40280544
2864	S9_41709138
2865	S9_43919254
2866	S9_47802340
2867	S9_48901881
2868	S9_49766992
2869	S9_49767256
2870	S9_50208004
2871	S9_52395592
2872	S9_52479658
2873	S9_53720860
2874	S9_54789486
2875	S9_55344695
2876	S9_56721876
2877	S9_57297876
2878	S9_57843091
2879	S9_58058408
2880	S9_58059194
2881	S9_60124973
2882	S9_60129695
2883	S9_61069827
2884	S9_61825440
2885	S9_63579790
2886	S9_64104330
2887	S9_64867584
2888	S9_64959509
2889	S9_65272987
2890	S9_65338918
2891	S9_66279850
2892	S9_67770180
2893	S9_67770183
2894	S9_68464022
2895	S9_69792863
2896	S9_70821607
2897	S9_71660159
2898	S9_71660203
2899	S9_72415924
2900	S9_72425431
2901	S9_73469091
2902	S9_73471605
2903	S9_74025757
2904	S9_75183622
2905	S9_78077421
2906	S9_79162421
2907	S9_80225294
2908	S9_80979684
2909	S9_82060050
2910	S9_84098687
2911	S9_84626087
2912	S9_86904081
2913	S9_87477293

2914	S9_88977131
2915	S9_89637882
2916	S9_90247173
2917	S9_99332270
2918	S9_100039508
2919	S9_100040477
2920	S9_100040889
2921	S9_101255087
2922	S9_101343170
2923	S9_102132744
2924	S9_103482509
2925	S9_105263301
2926	S9_106527674
2927	S9_108337664
2928	S9_109861761
2929	S9_112852113
2930	S9_113478789
2931	S9_113951205
2932	S9_119679030
2933	S9_120685580
2934	S9_122045749
2935	S9_123574995
2936	S9_125679358
2937	S9_126319033
2938	S9_126926829
2939	S9_127594958
2940	S9_128209745
2941	S9_128730072
2942	S9_129706294
2943	S9_131657574
2944	S9_131796213
2945	S9_132845912
2946	S9_133790017
2947	S9_135026181
2948	S9_135756726
2949	S9_138722454
2950	S9_139672178
2951	S9_141389123
2952	S9_142357707
2953	S9_143853484
2954	S9_144398269
2955	S9_145587538
2956	S9_146372708
2957	S9_147165007
2958	S9_147807922
2959	S9_147808293
2960	S9_147834539
2961	S9_147972664
2962	S9_148402666
2963	S9_149257713
2964	S9_149258551
2965	S9_150780570
2966	S9_151946596
2967	S9_153476143
2968	S9_153476146
2969	S9_153476150
2970	S9_153476194
2971	S9_154196841
2972	S9_154622761
2973	S9_155363145
2974	S9_155370602
2975	S9_156023571
2976	S9_157232161
2977	S9_162392308
2978	S9_164542711
2979	S9_165169794
2980	S9_167451209
2981	S9_168764880
2982	S9_169435017
2983	S9_169847017
2984	S9_171229640
2985	S9_172121707
2986	S9_173450106
2987	S9_174852881
2988	S9_175683004
2989	S9_176453293
2990	S9_177545041
2991	S9_178041461
2992	S9_179363535
2993	S9_180337354
2994	S9_181200232
2995	S9_181608026
2996	S9_183177173
2997	S9_183227293
2998	S9_183738973
2999	S9_183850198
3000	S9_183850253
3001	S9_184409778
3002	S9_185432681
3003	S9_189241921
3004	S9_190108087
3005	S9_192118012
3006	S9_192669138
3007	S9_195133896
3008	S9_196426332
3009	S9_199629332
3010	S9_200141296
3011	S9_202355643
3012	S9_206095109
3013	S9_207224549
3014	S9_207224555
3015	S9_207732117
3016	S9_208926342
3017	S9_210106083
3018	S9_212241056
3019	S9_214366390
3020	S9_215633145
3021	S9_217798252
3022	S9_218718255
3023	S9_219745252
3024	S9_219745258
3025	S9_219745261
3026	S9_219745263
3027	S9_220344982
3028	S9_220486503
3029	S9_220883534
3030	S9_220886294
3031	S9_223477724
3032	S9_224243754
3033	S9_224243772
3034	S9_225319908
3035	S9_228141695
3036	S9_228316687
3037	S9_228316688
3038	S9_231677201
3039	S9_231839292
3040	S9_231867235
3041	S9_233015433
3042	S9_233927071
3043	S9_234284337
3044	S9_234613510
3045	S9_235175328
3046	S9_236667740
3047	S9_237502597
3048	S9_237969439
3049	S9_238239014
3050	S9_238239034
3051	S9_239403872
3052	S9_239882981
3053	S9_240259758
3054	S9_240931547
3055	S9_241196182
3056	S9_241555800
3057	S9_241612448
3058	S9_242080656
3059	S9_242709604
3060	S9_243000839
3061	S9_243436326
3062	S9_243476487
3063	S9_244139073
3064	S9_245307037
3065	S9_245889175
3066	S9_246398797
3067	S9_246439293
3068	S9_246990858
3069	S9_246997423
3070	S9_247376642
3071	S9_247879245
3072	S9_248463251
3073	S9_248559079
3074	S9_248704920
3075	S9_249095364
3076	S9_249276357
3077	S9_249623035
3078	S9_249688835
3079	S9_250224139
3080	S9_250237861
3081	S9_250347649
3082	S9_250430196
3083	S9_250453605
3084	S9_250631180
3085	S9_250837759
3086	S9_250892099
3087	S9_250994503
3088	S9_251179493
3089	S9_251355046
3090	S9_251392910
3091	S9_251560412
3092	S9_251562788
3093	S9_251922242
3094	S9_252125215
3095	S9_252201450
3096	S9_252458493
3097	S9_252458596
3098	S10_171784
3099	S10_171803
3100	S10_180750
3101	S10_243910
3102	S10_742208
3103	S10_773413
3104	S10_1287569
3105	S10_1300848
3106	S10_1393618
3107	S10_1680064
3108	S10_1728523
3109	S10_1829571
3110	S10_2376684
3111	S10_2429100
3112	S10_3102381
3113	S10_3142129
3114	S10_3198143
3115	S10_3837795
3116	S10_3899418
3117	S10_3942557
3118	S10_4021018
3119	S10_4021182
3120	S10_4611252
3121	S10_5095608
3122	S10_5128462
3123	S10_5746440
3124	S10_6190070
3125	S10_7795516
3126	S10_7881667
3127	S10_8057495
3128	S10_8068831
3129	S10_9018165
3130	S10_9996933
3131	S10_10555789
3132	S10_10750084
3133	S10_11049757
3134	S10_11093400
3135	S10_11093401
3136	S10_11189476
3137	S10_11649573
3138	S10_12193494
3139	S10_12775134
3140	S10_12779440
3141	S10_13943221
3142	S10_14819448
3143	S10_15208941
3144	S10_15208979
3145	S10_15846307
3146	S10_17759983
3147	S10_17877722
3148	S10_18201660
3149	S10_18572053
3150	S10_19149714
3151	S10_19904984
3152	S10_20739754
3153	S10_21418423
3154	S10_22128621
3155	S10_23771292
3156	S10_26058571
3157	S10_28424451
3158	S10_29299300
3159	S10_29299301
3160	S10_29299306
3161	S10_29356559
3162	S10_29474331
3163	S10_30100818
3164	S10_31676332
3165	S10_31676350
3166	S10_32307896
3167	S10_33534094
3168	S10_34178212
3169	S10_35879195
3170	S10_37570873
3171	S10_38075796
3172	S10_40849601
3173	S10_43250558
3174	S10_44993809
3175	S10_45840174
3176	S10_46710175
3177	S10_46886392
3178	S10_48272746

3179	S10_49542685
3180	S10_51672762
3181	S10_52388410
3182	S10_52555153
3183	S10_52658079
3184	S10_53697532
3185	S10_55629912
3186	S10_56681009
3187	S10_57598156
3188	S10_58129483
3189	S10_58879898
3190	S10_61013457
3191	S10_61503732
3192	S10_61503734
3193	S10_62476182
3194	S10_63317339
3195	S10_64109708
3196	S10_67898251
3197	S10_70863131
3198	S10_71404230
3199	S10_73399065
3200	S10_74424671
3201	S10_74829512
3202	S10_75309117
3203	S10_76624285
3204	S10_80888235
3205	S10_84000147
3206	S10_87219403
3207	S10_89411348
3208	S10_90287438
3209	S10_91461967
3210	S10_93767269
3211	S10_95650352
3212	S10_96247722
3213	S10_97106085
3214	S10_98672973
3215	S10_100334730
3216	S10_101232253
3217	S10_101951111
3218	S10_102573387
3219	S10_103531909
3220	S10_104734730
3221	S10_106905862
3222	S10_109626627
3223	S10_111113293
3224	S10_112474756
3225	S10_116922261
3226	S10_117785399
3227	S10_120679326
3228	S10_120737310
3229	S10_121649494
3230	S10_125422832
3231	S10_127063128
3232	S10_127309446
3233	S10_128970565
3234	S10_131357488
3235	S10_132150144
3236	S10_132403038
3237	S10_133893513
3238	S10_135020751
3239	S10_136244179
3240	S10_140739801
3241	S10_140739862
3242	S10_141521070
3243	S10_143097955
3244	S10_143646682
3245	S10_144036441
3246	S10_145852091
3247	S10_146422764
3248	S10_147183693
3249	S10_147791028
3250	S10_149150243
3251	S10_150688061
3252	S10_153671714
3253	S10_158390582
3254	S10_161111286
3255	S10_161797275
3256	S10_163611079
3257	S10_164801236
3258	S10_166166726
3259	S10_166801032
3260	S10_167142223
3261	S10_167158624
3262	S10_168621280
3263	S10_169474771
3264	S10_172447427
3265	S10_173132894
3266	S10_174292649
3267	S10_175557537
3268	S10_176373267
3269	S10_177148594
3270	S10_178118007
3271	S10_178723463
3272	S10_179461737
3273	S10_179803258
3274	S10_179803287
3275	S10_181354759
3276	S10_181391360
3277	S10_181984276
3278	S10_182485522
3279	S10_184484317
3280	S10_185217541
3281	S10_185434124
3282	S10_185995661
3283	S10_186095022
3284	S10_187583317
3285	S10_187992495
3286	S10_188322973
3287	S10_188534046
3288	S10_189165081
3289	S10_189885653
3290	S10_189885713
3291	S10_190488062
3292	S10_191296845
3293	S10_192358088
3294	S10_192695297
3295	S10_192992160
3296	S10_193756775
3297	S10_194070751
3298	S10_194095274
3299	S10_194269682
3300	S10_194554915
3301	S10_194568906
3302	S10_194876265
3303	S10_194919081
3304	S10_194919099
3305	S10_195586187
3306	S10_196196656
3307	S10_196324449
3308	S10_196851898
3309	S10_197491323
3310	S10_199702712
3311	S10_200479413
3312	S10_200507494
3313	S10_201082266
3314	S10_201405234
3315	S10_202402286
3316	S10_202402390
3317	S10_203005200
3318	S10_203547801
3319	S10_204079966
3320	S10_204655935
3321	S10_205347549
3322	S10_205813014
3323	S10_205965701
3324	S10_205965848
3325	S10_207527394
3326	S10_208361180
3327	S10_208434279
3328	S10_209444944
3329	S10_209947771
3330	S10_209948043
3331	S10_210601644
3332	S10_210820058
3333	S10_211391239
3334	S10_212571516
3335	S10_213570626
3336	S10_213572331
3337	S10_214398103
3338	S10_215238239
3339	S10_215888920
3340	S10_216218911
3341	S10_216675026
3342	S10_217213966
3343	S10_217280297
3344	S10_217429868
3345	S10_217874066
3346	S10_217933086
3347	S10_218091065
3348	S10_218145664
3349	S10_219362689
3350	S10_219620068
3351	S10_219969313
3352	S10_220539735
3353	S10_220704372
3354	S10_220849011
3355	S10_221465316
3356	S10_221954268
3357	S10_221955948
3358	S10_222531571
3359	S10_222531848
3360	S10_222531919
3361	S10_223170766
3362	S10_223644170
3363	S10_223994418
3364	S10_224041371
3365	S10_224638149
3366	S10_224812801
3367	S10_225291680
3368	S10_225291755
3369	S10_225912363
3370	S10_226647137
3371	S10_227150402
3372	S10_227264380
3373	S10_227485319
3374	S10_227940683
3375	S10_228130183
3376	S10_228131774
3377	S10_228246554
3378	S10_228543087
3379	S10_229163944
3380	S10_229225480
3381	S10_229575810
3382	S10_229708832
3383	S10_229825698
3384	S10_229869201
3385	S10_229971366
3386	S10_230265319
3387	S10_230486293
3388	S10_230829317
3389	S10_231318842
3390	S10_231323330
3391	S10_231323348
3392	S10_231409473
3393	S10_231501629
3394	S10_231928645
3395	S10_232016614
3396	S10_232103809
3397	S10_232420839
3398	S10_232420857
3399	S10_232430604
3400	S10_232558912
3401	S10_232958931
3402	S10_232985480
3403	S11_328881
3404	S11_725943
3405	S11_725959
3406	S11_1203526
3407	S11_1279225
3408	S11_1900191
3409	S11_3208507
3410	S11_3229946
3411	S11_3944409
3412	S11_4563882
3413	S11_4995525
3414	S11_5659704
3415	S11_6356824
3416	S11_6714213
3417	S11_7106856
3418	S11_7237636
3419	S11_7792344
3420	S11_8341614
3421	S11_8442912
3422	S11_9074102
3423	S11_9566249
3424	S11_9566282
3425	S11_9743942
3426	S11_9744034
3427	S11_10114057
3428	S11_10268715
3429	S11_10388370
3430	S11_10610781
3431	S11_10715785
3432	S11_10717003
3433	S11_11393904
3434	S11_11567377
3435	S11_11567379
3436	S11_11567380
3437	S11_11567394
3438	S11_12323091
3439	S11_12924924
3440	S11_13272278
3441	S11_13432204
3442	S11_14337574
3443	S11_14659305

3444	S11_14659326	3497	S11_49453534	3550	S11_115786878	3603	S11_182811253	3656	S11_224324370
3445	S11_14659347	3498	S11_50164821	3551	S11_116359838	3604	S11_183871486	3657	S11_226051062
3446	S11_14690235	3499	S11_50861601	3552	S11_117209054	3605	S11_185057220	3658	S11_227856780
3447	S11_14690440	3500	S11_51800589	3553	S11_117209060	3606	S11_185835398	3659	S11_228718147
3448	S11_14898039	3501	S11_52571848	3554	S11_117274200	3607	S11_186432698	3660	S11_229521214
3449	S11_15123105	3502	S11_53195862	3555	S11_119073181	3608	S11_187732926	3661	S11_229620377
3450	S11_15697712	3503	S11_54008870	3556	S11_120619095	3609	S11_188241965	3662	S11_229638528
3451	S11_16563298	3504	S11_55626893	3557	S11_122202421	3610	S11_189182804	3663	S11_232287643
3452	S11_16563312	3505	S11_57615030	3558	S11_122555391	3611	S11_190304442	3664	S11_233355845
3453	S11_17136754	3506	S11_58310298	3559	S11_126263804	3612	S11_191812436	3665	S11_234259382
3454	S11_17699774	3507	S11_58585926	3560	S11_128237240	3613	S11_191817849	3666	S11_234829790
3455	S11_17748058	3508	S11_58882739	3561	S11_130126075	3614	S11_191918228	3667	S11_235383884
3456	S11_19323926	3509	S11_59684076	3562	S11_131691043	3615	S11_193269672	3668	S11_235913551
3457	S11_19908724	3510	S11_61164052	3563	S11_132523238	3616	S11_194405376	3669	S11_236713224
3458	S11_20238041	3511	S11_62162037	3564	S11_133026551	3617	S11_194405414	3670	S11_236875980
3459	S11_20805644	3512	S11_62854383	3565	S11_133588503	3618	S11_194405639	3671	S11_237447257
3460	S11_21322890	3513	S11_63722883	3566	S11_133643187	3619	S11_194931129	3672	S11_237635491
3461	S11_21323908	3514	S11_65513064	3567	S11_134620652	3620	S11_195537124	3673	S11_238659122
3462	S11_22030945	3515	S11_66072576	3568	S11_135350284	3621	S11_195537242	3674	S11_239983996
3463	S11_22591487	3516	S11_66444884	3569	S11_136211524	3622	S11_195537251	3675	S11_240848511
3464	S11_24864770	3517	S11_67247690	3570	S11_139412411	3623	S11_196830979	3676	S11_242085137
3465	S11_25925761	3518	S11_67249603	3571	S11_139412852	3624	S11_197653682	3677	S11_242666416
3466	S11_26787264	3519	S11_67249784	3572	S11_142769346	3625	S11_198962168	3678	S11_243169605
3467	S11_27518636	3520	S11_69417454	3573	S11_144254771	3626	S11_198962216	3679	S11_243672897
3468	S11_27761130	3521	S11_73300172	3574	S11_146193829	3627	S11_199702611	3680	S11_244058859
3469	S11_28614206	3522	S11_75532146	3575	S11_146748980	3628	S11_203400830	3681	S11_244229643
3470	S11_29771356	3523	S11_76120918	3576	S11_147578286	3629	S11_203961181	3682	S11_244230867
3471	S11_30399356	3524	S11_76859674	3577	S11_149930700	3630	S11_204795034	3683	S11_244279024
3472	S11_31323995	3525	S11_77975790	3578	S11_152975717	3631	S11_206874321	3684	S11_244338911
3473	S11_32937563	3526	S11_78710932	3579	S11_153489937	3632	S11_207570332	3685	S11_244998845
3474	S11_34178116	3527	S11_79352326	3580	S11_154887023	3633	S11_208525627	3686	S11_245078220
3475	S11_34287619	3528	S11_81820322	3581	S11_156235654	3634	S11_208639228	3687	S11_245766574
3476	S11_34758133	3529	S11_82949092	3582	S11_159448608	3635	S11_209319381	3688	S11_246440996
3477	S11_34878301	3530	S11_83592462	3583	S11_160576264	3636	S11_211309293	3689	S11_246476981
3478	S11_34918138	3531	S11_84950337	3584	S11_164200080	3637	S11_211919191	3690	S11_246715528
3479	S11_35899254	3532	S11_85284937	3585	S11_164944294	3638	S11_212811494	3691	S11_246730323
3480	S11_36015173	3533	S11_92395068	3586	S11_165011973	3639	S11_212812944	3692	S11_246904841
3481	S11_36524515	3534	S11_92751430	3587	S11_165011978	3640	S11_213082656	3693	S11_246904866
3482	S11_37545773	3535	S11_94175198	3588	S11_166591326	3641	S11_213332102	3694	S11_247547926
3483	S11_38138077	3536	S11_95981310	3589	S11_166591445	3642	S11_216573789	3695	S11_248078404
3484	S11_39215000	3537	S11_96567180	3590	S11_169682333	3643	S11_217313085	3696	S11_248179242
3485	S11_40122458	3538	S11_96567188	3591	S11_170134275	3644	S11_217851084	3697	S11_248179259
3486	S11_40128945	3539	S11_103275947	3592	S11_170531489	3645	S11_217851085	3698	S11_248441702
3487	S11_41927757	3540	S11_105821501	3593	S11_170531512	3646	S11_217851095	3699	S11_248687427
3488	S11_42077538	3541	S11_107086282	3594	S11_171711477	3647	S11_217851103	3700	S11_248696246
3489	S11_43156510	3542	S11_107908730	3595	S11_173613476	3648	S11_218551647	3701	S11_248809688
3490	S11_43248560	3543	S11_107908805	3596	S11_174438071	3649	S11_219500910	3702	S11_249430902
3491	S11_44908982	3544	S11_109523873	3597	S11_174667996	3650	S11_219501091	3703	S11_249694260
3492	S11_45470452	3545	S11_109549811	3598	S11_176329533	3651	S11_220213588	3704	S11_249757813
3493	S11_45516660	3546	S11_110437456	3599	S11_179043541	3652	S11_220932237	3705	S11_249828534
3494	S11_45516825	3547	S11_111436012	3600	S11_179174271	3653	S11_222613746	3706	S11_250089855
3495	S11_46291552	3548	S11_114380982	3601	S11_179958711	3654	S11_223041461	3707	S11_250389266
3496	S11_48018210	3549	S11_115166226	3602	S11_180568639	3655	S11_223554543	3708	S11_250959491

3709	S11_251501782
3710	S11_251598058
3711	S11_251812245
3712	S11_251850109
3713	S11_252095723
3714	S11_252665257
3715	S11_252665795
3716	S11_252798834
3717	S11_253249265
3718	S11_253249295
3719	S11_253730251
3720	S11_253779338
3721	S11_254611455
3722	S11_255298592
3723	S11_255912944
3724	S11_255988322
3725	S11_256285335
3726	S11_257047107
3727	S11_257158898
3728	S11_257174908
3729	S11_257653511
3730	S11_257687575
3731	S11_257903218
3732	S11_257903879
3733	S11_258260851
3734	S11_258295348
3735	S11_258853958
3736	S12_381125
3737	S12_412783
3738	S12_545631
3739	S12_1563725
3740	S12_2126233
3741	S12_2273213
3742	S12_2776021
3743	S12_3119394
3744	S12_3407700
3745	S12_3407773
3746	S12_3409794
3747	S12_3452650
3748	S12_3844726
3749	S12_3961099
3750	S12_3961126
3751	S12_4582600
3752	S12_4691483
3753	S12_4691486
3754	S12_4761165
3755	S12_4974290
3756	S12_5306711
3757	S12_5491364
3758	S12_5757901
3759	S12_5900256
3760	S12_6340823
3761	S12_6342563
3762	S12_6387912
3763	S12_6387915
3764	S12_6426034
3765	S12_6525010
3766	S12_6661180
3767	S12_6931350
3768	S12_6995753
3769	S12_7037727
3770	S12_7432812
3771	S12_7436796
3772	S12_7618700
3773	S12_7950816
3774	S12_8222161
3775	S12_8402500
3776	S12_9134525
3777	S12_9394124
3778	S12_9771381
3779	S12_10117958
3780	S12_10140864
3781	S12_10760297
3782	S12_10820282
3783	S12_11021887
3784	S12_11396642
3785	S12_11417428
3786	S12_11478132
3787	S12_11478245
3788	S12_11827387
3789	S12_11864700
3790	S12_12260394
3791	S12_12260488
3792	S12_12311854
3793	S12_13128484
3794	S12_13846215
3795	S12_14354006
3796	S12_15040360
3797	S12_15588285
3798	S12_16118854
3799	S12_16987250
3800	S12_17747884
3801	S12_18101908
3802	S12_18691940
3803	S12_18830407
3804	S12_19362694
3805	S12_20069779
3806	S12_20274418
3807	S12_20469924
3808	S12_20809991
3809	S12_22200015
3810	S12_22834295
3811	S12_23114637
3812	S12_23207861
3813	S12_23781581
3814	S12_23861916
3815	S12_23986010
3816	S12_24399334
3817	S12_24951819
3818	S12_26592114
3819	S12_27856033
3820	S12_29370856
3821	S12_29743707
3822	S12_30874391
3823	S12_32189654
3824	S12_32635084
3825	S12_33464558
3826	S12_34816110
3827	S12_34910460
3828	S12_34910465
3829	S12_35332798
3830	S12_35864251
3831	S12_35914651
3832	S12_37068053
3833	S12_37498542
3834	S12_37590240
3835	S12_38159084
3836	S12_38990322
3837	S12_39614790
3838	S12_40129974
3839	S12_42277153
3840	S12_42395578
3841	S12_42803550
3842	S12_43074312
3843	S12_44300709
3844	S12_44307621
3845	S12_44363141
3846	S12_44844564
3847	S12_45707121
3848	S12_47060797
3849	S12_47994250
3850	S12_48844736
3851	S12_49058693
3852	S12_49994162
3853	S12_50365719
3854	S12_50608360
3855	S12_51442974
3856	S12_52077647
3857	S12_53835614
3858	S12_54487953
3859	S12_55715022
3860	S12_55728740
3861	S12_56702257
3862	S12_57472358
3863	S12_58068130
3864	S12_59153948
3865	S12_60353524
3866	S12_63963356
3867	S12_65418196
3868	S12_66058184
3869	S12_67085061
3870	S12_67984590
3871	S12_69753133
3872	S12_70536812
3873	S12_71300728
3874	S12_75146026
3875	S12_77563686
3876	S12_78455778
3877	S12_79845855
3878	S12_81844614
3879	S12_82385247
3880	S12_83269602
3881	S12_84119946
3882	S12_86107871
3883	S12_87938477
3884	S12_89469404
3885	S12_90777989
3886	S12_92106316
3887	S12_93733539
3888	S12_94744987
3889	S12_94919595
3890	S12_94946118
3891	S12_95871807
3892	S12_98679907
3893	S12_100576867
3894	S12_102775249
3895	S12_102776780
3896	S12_104485636
3897	S12_105217582
3898	S12_105775913
3899	S12_105780999
3900	S12_106949922
3901	S12_107575130
3902	S12_108134851
3903	S12_108796635
3904	S12_110247005
3905	S12_112421444
3906	S12_113165076
3907	S12_114287856
3908	S12_115135272
3909	S12_116579280
3910	S12_117499282
3911	S12_118764007
3912	S12_120178735
3913	S12_121004752
3914	S12_122009585
3915	S12_124642905
3916	S12_125930858
3917	S12_130787923
3918	S12_131327502
3919	S12_131845614
3920	S12_135261625
3921	S12_136375416
3922	S12_137942647
3923	S12_138461734
3924	S12_140953325
3925	S12_143851093
3926	S12_145575105
3927	S12_146233037
3928	S12_147882737
3929	S12_147882749
3930	S12_147892082
3931	S12_147897201
3932	S12_149009104
3933	S12_149625298
3934	S12_151089906
3935	S12_152156128
3936	S12_153503430
3937	S12_155962622
3938	S12_157376835
3939	S12_157722771
3940	S12_158542626
3941	S12_159405845
3942	S12_160734396
3943	S12_162152808
3944	S12_163135566
3945	S12_164786789
3946	S12_166315394
3947	S12_167505462
3948	S12_168273094
3949	S12_168989003
3950	S12_170336792
3951	S12_171666164
3952	S12_172615786
3953	S12_174325266
3954	S12_175040624
3955	S12_175738951
3956	S12_176246852
3957	S12_178003886
3958	S12_179091507
3959	S12_179763516
3960	S12_180639009
3961	S12_180797100
3962	S12_180836832
3963	S12_181298730
3964	S12_182584891
3965	S12_183698580
3966	S12_184988186
3967	S12_185657816
3968	S12_191226512
3969	S12_191955052
3970	S12_194637563
3971	S12_195915304
3972	S12_196309444
3973	S12_196498993

3974	S12_196883708
3975	S12_197692559
3976	S12_198523417
3977	S12_199052675
3978	S12_199857480
3979	S12_200047408
3980	S12_200047462
3981	S12_200368163
3982	S12_200784100
3983	S12_201544460
3984	S12_201545169
3985	S12_203983726
3986	S12_205330324
3987	S12_206003761
3988	S12_206080742
3989	S12_206581174
3990	S12_207504616
3991	S12_207504890
3992	S12_208197301
3993	S12_209360988
3994	S12_209834094
3995	S12_210086839
3996	S12_211031110
3997	S12_214166392
3998	S12_214166514
3999	S12_214805467
4000	S12_215221059
4001	S12_215702153
4002	S12_216346859
4003	S12_216482451
4004	S12_216482465
4005	S12_216482469
4006	S12_216482472
4007	S12_216482493
4008	S12_216688243
4009	S12_217201143
4010	S12_217764602
4011	S12_217764718
4012	S12_217851965
4013	S12_218418440
4014	S12_218970894
4015	S12_219200395
4016	S12_220041747
4017	S12_220081187
4018	S12_220819010
4019	S12_220926920
4020	S12_221372492
4021	S12_221459037
4022	S12_221747349
4023	S12_222326338
4024	S12_223095752
4025	S12_224821152
4026	S12_224956340
4027	S12_224956341
4028	S12_224965826
4029	S12_225448621
4030	S12_226015817
4031	S12_226041586
4032	S12_226217278
4033	S12_226342682
4034	S12_226343462
4035	S12_226434628
4036	S12_226434630
4037	S12_226524770
4038	S12_227020565
4039	S12_227321672
4040	S12_227321679
4041	S12_227321680
4042	S12_227518858
4043	S12_227780636
4044	S12_227789034
4045	S12_228170640
4046	S12_228631581
4047	S12_229067475
4048	S12_229109540
4049	S12_229171093
4050	S12_229386457
4051	S12_229534703
4052	S12_229776281
4053	S12_229901362
4054	S12_230025750
4055	S12_230697161
4056	S12_230719878
4057	S12_230719883
4058	S12_230840374
4059	S12_231322364
4060	S12_231467159
4061	S12_231968491
4062	S12_232064444
4063	S12_232638258
4064	S12_232880901
4065	S12_233004672
4066	S12_233155168
4067	S12_233553532
4068	S12_233730308
4069	S12_234034009
4070	S12_234130982
4071	S12_234132232
4072	S12_234212307
4073	S12_234212316
4074	S12_234255325
4075	S12_234342318
4076	S12_234342625
4077	S12_234589234
4078	S12_234786277
4079	S12_234793863
4080	S12_235058958
4081	S12_235431103
4082	S12_235565159
4083	S12_235587286
