# Supplementary material for: Genetic diversity, population structure, and relationships in a collection of pepper (Capsicum spp.) landraces from the Spanish centre of diversity revealed by genotyping-by-sequencing (GBS)
Source: Hortic Res. 2019 May 1;6:54. doi: 10.1038/s41438-019-0132-8 (PMC6491490; doi:10.1038/s41438-019-0132-8)
Supplement: Supplementary file 6 — Supplementary Data: Table 4 [file 41438_2019_132_MOESM6_ESM.pdf]

For all 148 accessions

Accession	Assigned Cluster
fra_tendre	1
ita_senise	1
ser_at	1
sp_00057	1
sp_00060	1
sp_00637	1
sp_01844	1
sp_04036	1
sp_04322	1
sp_04331	1
sp_04335	1
sp_05041	1
sp_05057	1
sp_05103	1
sp_05114	1
sp_05121	1
sp_05126	1
sp_10186	1
sp_10368	1
sp_10451	1
sp_10540	1
sp_10582	1
sp_10600	1
sp_11038	1
sp_11205	1
sp_11500	1
sp_11558	1
sp_11881	1
sp_bola	1
sp_cala	1
sp_guer	1
sp_inf	1
sp_ital	1
sp_mojo	1
sp_naj	1
sp_pad	1
sp_piq	1
sp_reus	1
sp_vlc	1
bul_kap	2
bul_siv	2
fra_dll	2
fra_prb	2
ind_torp	2
mex_96d	2
mex_ancho	2
mex_mulato	2
mex_pasilla	2

Assigned Cluster	Number Individuals
Cluster 1	39
Cluster 2	22
Cluster 3	14
Cluster 4	44
Cluster 5	9
Cluster 6	3
Cluster 7	17

K = 1 to K = 20	BIC
K=1	917,00
K=2	843,38
K=3	833,60
K=4	822,37
K=5	821,18
K=6	820,10
**K=7**	**819,63**
K=8	819,79
K=9	820,43
K=10	820,86
K=11	822,36
K=12	824,10
K=13	825,80
K=14	827,46
K=15	829,44
K=16	831,57
K=17	833,67
K=18	835,62
K=19	838,10
K=20	840,69

mex_puya	2
mu_chile	2
sp_11531	2
sp_ibarra	2
sp_pic	2
tur_aci	2
usa_13293	2
usa_64	2
usa_chima	2
usa_chimayo	2
usa_conq	2
usa_jim	2
usa_num	2
mex_arb	3
mex_scm	3
mex_serra	3
mex_serrano	3
mex_v1196	3
mu_espin	3
mu_jal	3
sp_jal	3
sp_pascual	3
sp_rsw	3
sri_ka	3
usa_cand	3
usa_jap	3
usa_lrc	3
bul_rat	4
fra_petit	4
ita_carg	4
ita_carr	4
ita_cuneo	4
ita_giallo	4
ita_top	4
sp_00614	4
sp_01319	4
sp_01814	4
sp_01834	4
sp_01862	4
sp_04329	4
sp_04348	4
sp_04349	4
sp_04507	4
sp_05030	4
sp_05083	4
sp_05109	4
sp_05113	4
sp_10183	4
sp_10185	4
sp_10447	4
sp_10599	4
sp_10946	4
sp_11092	4
sp_11213	4

sp_11267	4
sp_11528	4
sp_11630	4
sp_11751	4
sp_11814	4
sp_13004	4
sp_13009	4
sp_13636	4
sp_13638	4
sp_arnoia	4
sp_bier	4
sp_cat	4
sp_cwr	4
sp_cwy	4
sp_fresno	4
sp_lamr	4
sp_lamy	4
bol_037	5
bol_039	5
bol_103	5
bol_120	5
bol_174	5
bol_175	5
bol_178	5
bol_ari	5
bol_toro	5
mex_q1078	6
mex_s1120	6
usa_a1003	6
bol_144	7
bol_198	7
eq_973	7
eq_994	7
mex_c1333	7
mex_n1411	7
mex_o1430	7
peru_cabra	7
peru_cer	7
peru_char	7
peru_limo	7
peru_mis	7
peru_moche	7
usa_hab	7
usa_pi	7
ven_chi	7
ven_dulce	7

For 122 accessions only

Accession	Assigned Cluster
mex_q1078	1
mex_s1120	1
usa_a1003	1
bul_kap	2
bul_siv	2
fra_dll	2
fra_prb	2
ind_torp	2
mex_96d	2
mex_ancho	2
mex_mulato	2
mex_pasilla	2
mex_puya	2
mu_chile	2
sp_11531	2
sp_ibarra	2
sp_pic	2
tur_aci	2
usa_13293	2
usa_64	2
usa_chima	2
usa_chimayo	2
usa_conq	2
usa_jim	2
usa_num	2
mex_arb	3
mex_scm	3
mex_serra	3
mex_serrano	3
mex_v1196	3
mu_espin	3
mu_jal	3
sp_jal	3
sp_pascual	3
sp_rsw	3
sri_ka	3
usa_cand	3
usa_jap	3
usa_lrc	3
bul_rat	4
fra_petit	4
ita_carg	4
ita_carr	4
ita_cuneo	4
ita_giallo	4
ita_top	4
sp_00614	4
sp_01319	4

Assigned Cluster	Number Individuals
Cluster 1	3
Cluster 2	22
Cluster 3	14
Cluster 4	44
Cluster 5	39

K = 1 to K = 20	BIC
K=1	691,06
K=2	681,02
K=3	680,14
K=4	679,31
**K=5**	**679,02**
K=6	679,29
K=7	680,00
K=8	681,60
K=9	683,24
K=10	685,23
K=11	687,04
K=12	688,86
K=13	690,93
K=14	693,08
K=15	695,22
K=16	697,63
K=17	700,06
K=18	702,12
K=19	704,83
K=20	707,07

sp_01814	4
sp_01834	4
sp_01862	4
sp_04329	4
sp_04348	4
sp_04349	4
sp_04507	4
sp_05030	4
sp_05083	4
sp_05109	4
sp_05113	4
sp_10183	4
sp_10185	4
sp_10447	4
sp_10599	4
sp_10946	4
sp_11092	4
sp_11213	4
sp_11267	4
sp_11528	4
sp_11630	4
sp_11751	4
sp_11814	4
sp_13004	4
sp_13009	4
sp_13636	4
sp_13638	4
sp_arnoia	4
sp_bier	4
sp_cat	4
sp_cwr	4
sp_cwy	4
sp_fresno	4
sp_lamr	4
sp_lamy	4
fra_tendre	5
ita_senise	5
ser_at	5
sp_00057	5
sp_00060	5
sp_00637	5
sp_01844	5
sp_04036	5
sp_04322	5
sp_04331	5
sp_04335	5
sp_05041	5
sp_05057	5
sp_05103	5
sp_05114	5
sp_05121	5
sp_05126	5
sp_10186	5
sp_10368	5

sp_10451	5
sp_10540	5
sp_10582	5
sp_10600	5
sp_11038	5
sp_11205	5
sp_11500	5
sp_11558	5
sp_11881	5
sp_bola	5
sp_cala	5
sp_guer	5
sp_inf	5
sp_ital	5
sp_mojo	5
sp_naj	5
sp_pad	5
sp_piq	5
sp_reus	5
sp_vlc	5
